# Real-time PCR-based serotyping of *Streptococcus agalactiae*

**DOI:** 10.1038/srep38523

**Published:** 2016-12-02

**Authors:** Kathleen M. Breeding, Bhavana Ragipani, Kun-Uk David Lee, Martin Malik, Tara M. Randis, Adam J. Ratner

**Affiliations:** 1 College of Physicians & Surgeons, Columbia University, New York, NY USA; 2 Department of Pediatrics, New York University School of Medicine, New York, NY USA; 3 Department of Microbiology, New York University School of Medicine, New York, NY USA

## Abstract

Group B *Streptococcus* (GBS) is an encapsulated, gram-positive pathogen that is an important cause of neonatal invasive infections, including sepsis and meningitis. There are ten known GBS serotypes based on distinct capsule compositions (Ia, Ib, II-IX), and current candidate capsular polysaccharide conjugate vaccines target only a subset of these. Serotyping of GBS isolates is important for understanding local epidemiology and for monitoring for serotype replacement or capsular switching. However, serotyping generally requires either latex agglutination, multiplex PCR with analysis of band sizes, or analysis of whole genome sequences–all techniques that are either expensive or not widely available. Here we report the development of a robust real-time PCR assay for determining GBS serotypes. Using both a diverse reference set of strains encompassing all ten serotypes and a collection of clinical isolates, we demonstrate concordance between real-time PCR serotyping and latex agglutination. We propose that real-time PCR serotyping represents an attractive alternative to current serotyping methods and may allow for improved acquisition of GBS serotype data.

*Streptococcus agalactiae* (Group B *Streptococcus* [GBS]) is a major cause of neonatal morbidity and mortality worldwide^1^,^2^. While the incidence of GBS early-onset sepsis has decreased substantially in the United States following the implementation of universal screening and intrapartum antimicrobial prophylaxis^3^,^4^, these represent resource-intensive policies that are subject to missed opportunities for prevention^5^,^6^. Because of these limitations, improved methods for prevention of GBS disease are urgently needed. Immunization with capsular polysaccharide (CPS)-protein conjugates can induce serotype-specific immunity^7^, and candidate GBS conjugate vaccines are currently being tested in clinical trials^8^.

The serotype of a GBS strain is determined by the genes in the *cps* locus, and 10 distinct serotypes (Ia, Ib, II-IX) have been described to date^9^,^10^. Understanding the serotype distribution of GBS in both disease and colonization states is crucial to inform rational vaccine policies and to detect events such as capsular switching^11^,^12^ and serotype replacement. Capsular typing of GBS is generally performed using latex agglutination with type-specific antibodies, though both multiplex PCR^13^,^14^,^15^ and flow cytometry^16^ strategies have been described. More recently, whole-genome sequences have been used to determine GBS serotypes^17^. Here we describe a novel TaqMan-based real-time PCR strategy for serotyping GBS based on detection of specific *cps* locus sequences and demonstrate its applicability using both a defined set of strains from a reference laboratory and a set of primary clinical isolates.

## Materials and Methods

### Bacterial strains and growth conditions

A total of 68 clinical GBS isolates were included in this study. Of these, 47 *S. agalactiae* serotype reference stains were provided by the *Streptococcus* laboratory at the Respiratory Diseases Branch of the Centers for Disease Control and Prevention (CDC). For some isolates, multilocus sequence type (MLST) determination was determined by the *Streptococcus* reference laboratory using established protocols (http://pubmlst.org/sagalactiae/). The remaining 21 isolates were deidentified sterile site isolates collected between 2010 and 2015 in New York City. All specimens were cultured on trypticase soy (TS) or 5% sheep blood agar plates at 37 °C and then grown overnight in TS media at 37 °C.

### Serotyping by latex agglutination

All GBS isolates were serotyped by latex agglutination with the Immulex Latex Agglutination Streptococcus B kit (Staten Serum Institute; Copenhagen, Denmark) according to the manufacturer’s instructions. Briefly, 10 μl of the latex reagent was added to a single colony of GBS suspended in 10 μl of saline. The reaction was rotated and interpreted as positive if agglutination was visible after 30 seconds.

### TaqMan real-time PCR serotyping

Primers and probes were designed to amplify unique regions of the polysaccharide capsular genes of each of the serotypes of *S. agalactiae* (Table 1). In order to generate these primer/probe sets, DNA sequences from specific capsular polysaccharide (cps) operons were obtained from National Center for Biotechnology Information databases (Table 1). Trimmed predicted *cpsE*-*cpsL* regions were aligned with using the CLUSTALW algorithm (Supplemental File 1). Specific primer and probe sequences and target regions were hand-selected from this multiple alignment of all 10 cps operons and are listed in Table 1. Oligonucleotides and probes were obtained from Integrated DNA Technologies (Coralville, IA). Oligonucleotides were unmodified and desalted, and probes were labeled with a fluorescent probe (5′ 6-carboxyfluorescein (6-FAM)) and two quenchers (internal ZEN^TM^, and 3′ Iowa Black® FQ) and purified by high-pressure liquid chromatography.

Genomic DNA was extracted from overnight cultures of GBS using the Qiagen DNeasy Blood and Tissue Kit according to the manufacturer’s instructions. PCR reactions were performed in a final volume of 20 μl and consisted of 10 μl Taqman Universal Mastermix (Applied Biosystems), 7.4 μl sterile water, 0.2 μl forward primer (100 μM stock), 0.2 μl reverse primer (100 μM stock), 0.2 μl probe (100 μM stock), and 2 μl GBS DNA (25 ng/μl, unless otherwise indicated in the text.) Triplicate reactions were performed on a StepOne Plus thermal cycler (Applied Biosystems) and analyzed using StepOne software. Reaction parameters were as follows: initial incubation at 50 °C for 2 minutes; initial denaturation at 95 °C for 10 minutes; 35 cycles of PCR at 95 °C for 15 seconds and 60 °C for 1 min. Positive reactions were defined as a cycle threshold (C_T_) < 30 for 50 ng DNA template/reaction. Negative control reactions (no DNA template) were included with every run. Results were compared to latex agglutination.

### Reaction sensitivity and interference by non-targeted strains

Sensitivity was evaluated by determining the cycle threshold on 10-fold serial dilutions of bacterial DNA from 5 ng to 50 pg per reaction. Potential interference was assessed by real-time PCR using each primer/probe set under the conditions above with a mixture of chromosomal DNA from all ten GBS serotypes (strains AR959 - AR968) at a final amount of 25 ng for each strain (250 ng total DNA) per reaction as template.

## Results

### Accuracy of real-time PCR serotyping using a validation set

We used a set of 47 validation strains (≥3 per serotype) from the CDC *Streptococcus* laboratory to determine whether the primer/probe combinations listed in Table 1 could be used for accurate serotyping. In all cases, there was detection of the predicted sequence using the real-time PCR protocol (Table 2) and no positive reactions with any of the other serotype primer/probe combinations (not shown). We confirmed the serotype of each of the 47 strains by latex agglutination, and there was 100% concordance between the PCR-based and latex agglutination methods for the validation set.

### Serotyping of clinical isolates

We used the real-time PCR serotyping assay on a collection of 21 clinical sterile-site GBS isolates (Table 3). In 21/21 (100%) of cases, a single serotype was detected at C_T_ < 30, and in all of those, the result was confirmed by latex agglutination.

### Sensitivity of real-time PCR serotyping and potential interference

**Figure 1:**
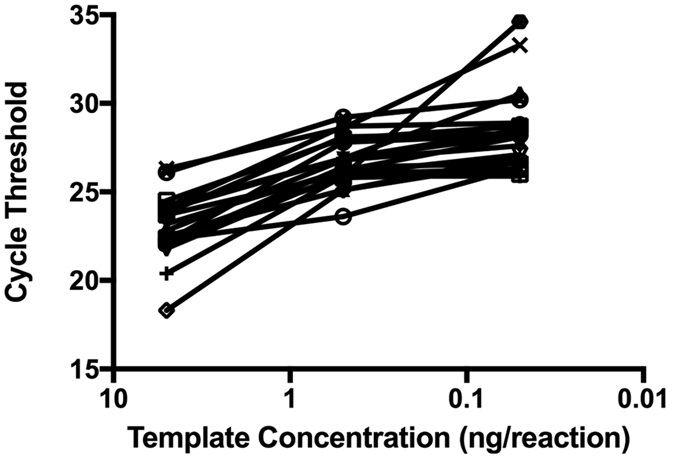
Serial dilution of GBS genomic DNA template demonstrates sensitivity of real-time PCR serotyping. Serotyping reactions were performed on 20 validation strains using 5 ng, 500 pg, and 50 pg of template per reaction. Cycle threshold increased with decreasing template amounts but remained detectable for all strains even at the lowest concentration tested.

In order to determine the sensitivity of the real-time PCR protocol, we performed serial dilutions of the extracted DNA from 20 of the validation strains, from 5 ng to 50 pg per reaction. As predicted, C_T_ increased as the total amount of bacterial DNA template in the reaction decreased (Fig. 1), though the majority of strains remained under the 30 cycle threshold, even at 50 pg per reaction. For all primer sets, detection of target sequences (C_T_ < 30) was robust to the inclusion of an excess of non-targeted GBS DNA.

## Discussion

Clinical microbiology laboratories routinely identify GBS but do not typically report serotype data. However, as the development of vaccines that target a subset of the ten known GBS serotypes proceeds, it will become increasingly important to understand the distribution of vaccine and non-vaccine serotypes and to monitor for capsular switching and serotype replacement. Non-nucleic acid-based laboratory methods for GBS serotyping techniques can be labor intensive and expensive, may require high-titer serotype-specific antisera and can create an added barrier for performing GBS serotype prevalence studies. Latex agglutination kits may be used for GBS serotyping in reference laboratories but are costly and not routinely available.

Molecular capsular typing techniques, including multiplex PCR^13^,^14^ and targeted analysis of whole genome sequences^17^, may be advantageous because of adaptability to newly available sequence information and relative ease of performance. In one prior study, real-time PCR was reported to distinguish among serotypes Ia, Ib, and III only^18^. We designed custom primer-probe sets for detection of all ten GBS serotypes. Unlike many prior methods, serotyping by real-time PCR is not based on operator interpretation of biochemical reactions and allows for specific identification of a GBS serotype despite interference from other bacterial DNA. Although the current assay is likely too technically demanding for routine laboratory adoption, it may be useful in the context of epidemiologic studies as an alternative to existing multiplex PCR assays. With further development, this novel method may allow for detection of GBS serotypes directly from clinical specimens without the need for culture. Limitations of this real-time PCR-based serotyping include an inability to identify new serotypes directly (as specific primer-probe sets are required for detection) and dependence on conservation of the targeted genetic regions within a particular serotype. Further validation of these primer-probe sets on a globally representative set of isolates is underway, as all of the tested strains originated in North America. Studies of larger, more diverse strain collections will be required to ensure that the primer-probe sets are sensitive and specific across an appropriate range of isolates.

## Additional Information

**How to cite this article**: Breeding, K. M. *et al*. Real-time PCR-based serotyping of *Streptococcus agalactiae*. *Sci. Rep.* **6**, 38523; doi: 10.1038/srep38523 (2016).

**Publisher's note:** Springer Nature remains neutral with regard to jurisdictional claims in published maps and institutional affiliations.

## Supplementary Material

Supplementary Information S1

## Figures and Tables

**Table 1:** Target genes, primer and probe sequences for GBS serotyping by real-time PCR.

	Sequence (5′-3′)	Target Gene	Amplicon Size (bp)	NCBI Accession
Ia-F	GTTTAAAAATCCTGATTTTGATAGAATTTTAGCAGCTTTTAAC	*cpsH*	207	CP000114.1
Ia-R	CTGATATTTTGAATATTATTATGCAAACAATAATAATATGTTCCCCCTA			
Ia-P	6-FAM-TCGTTGATT/ZEN/ATCGGTATAGTATCATTG GCT-IAbFQ			
Ib-F	GTATTAAATTCGTTATTTAGAAGTCCAGAATTTCATAGAGTCATTGC	*cpsH*	195	FO393392.1
Ib-R	GGCATAATAATATAGAAATCCTAAACAAGACAAAATAATTGCATTAAAC			
Ib-P	6-FAM-TGC ATT CAA/ZEN/TTCACTGGCAGTAGGG- IAbFQ			
II-F	CACATATATATTAAAGTTCACCCTAGAGATAACATTGACTACTCTAATC	*cpsK*	151	AAJO01000077.1
II-R	CTAATGCCGTGGAAAAATATGTAATCCCAACATCAAATT			
II-P	6-FAM-AATGCAACA/ZEN/GTAATACAAAGGAACATC CCT- IAbFQ			
III-F	GGAATTGTTCTTTATTTTTCTGCCT	*cpsI*	170	AL766849.1
III-R	ACTATACCAAAAGTTGAGAATAATAATACAATACTCCAATGA			
III-P	6-FAM-ATGTTACAC /ZEN/GCTCTTTGAGGAAATAGATCC- IAbFQ			
IV-F	GAAGAAAATATATATTTGCCATACAGTATATCATCTCCTTATTACAATTATC	*cpsK*	159	AF355776.1
IV-R	CATAGAATACCTTCTTTATTGGTACGTTTACATAAATCATCAATATTAAC			
IV-P	6-FAM-AGGGAACAG /ZEN/AGGAGATCAATAATTATATTGGC- IAbFQ			
V-F	CAAAATTCAATGAGAGAATGTTGTATTTTTTTGAGGCAATTC	*cpsO*	153	AE009948.1
V-R	CAATCATCTTCCCACATATATCTATTCCACCAAATACTTC			
V-P	6-FAM-ATTTTCCAC /ZEN/ATAATACATCTTTAATCTCTGCTG T- IAbFQ			
VI-F	GACAGTCTATTACGAAAGTATAAGAGCGATT	*cpsH*	219	HF952106.1
VI-R	AGCTTGTAGATTATCCTGTTTTGTTTGATAGCTTCTCTATATAG			
VI-P	6-FAM-CCCTCCAGT/ZEN/GTGGGAATATTTTTAGGTTCAC- IAbFQ			
VII-F	GAGGGCTTACCTCACGACAGGAGAAGTAAAAAATATAAAG	*cpsK*	160	AY376403.1
VII-R	GCTGCGTTAATAACAATACTGACTTTGGAGC			
VII-P	6-FAM-AGTCTTACC/ZEN/CAAGAACAAAAGTCTCTGATT- IAbFQ			
VIII-F	GACTAATGGTTAAGTATGCTAACTTGCTAATTTGTGATAGTAA	*cpsR*	152	AY375363
VIII-R	CTTGTCCTTAAAATTGTGTTTTGACTTTGTCAGATCAGTC			
VIII-P	6-FAM-ATGCTCCTA/ZEN/AAACAACCTACATCGCCTATG- IAbFQ			
IX-F	CATTGAGCAAAGAGAAAACAGTATATGTCAAAGGGC	*cpsO*	128	CGBY01000002
IX-R	ATGTTCAAGGATAAAATCTCTATTATGTTGCATTGCTTCA			
IX-P	6-FAM-AGTACTACC/ZEN/AGACAGTCATACAAAGAGAAT- IAbFQ			
		Sequences are presented 5' to 3' with probe modifications as indicated (6-carboxyfluorescein [6-FAM] fluorescent probe, internal ZEN^TM^ and Iowa Black^®^ FQ [IAbFQ] quenchers).		

**Table 2:** Cycle thresholds for real-time PCR serotyping of CDC validation strains.

Strain	Serotype	MLST	C_T_
20154637	Ia	ST1	19.40 (17.76–22.18)
20155889	Ia	ST23	17.61 (16.61–18.17)
20155226	Ia	ST19	17.56 (17.49–17.60)
2008232728	Ia		18.22 (17.98–18.65)
2014210282	Ia		18.55 (18.46–18.61)
20153631	Ib	ST1	16.24 (15.77–16.60)
20153550	Ib	ST8	17.62 (17.49–17.76)
20155787	Ib	ST12	16.75 (16.52–17.12)
2008232729	Ib		17.21 (17.08–17.41)
2014210284	Ib		19.54 (19.17–19.93)
20154198	II	ST1	16.83 (16.82–16.86)
20155757	II	ST22	16.90 (16.68–17.09)
20155810	II	ST28	16.33 (16.23–16.44)
2008232738	II		19.76 (19.25–20.54)
2014210280	II		17.54 (17.42–17.68)
20154686	III	ST17	19.53 (18.99–20.60)
20154526	III	ST19	18.23 (17.71–18.82)
20155871	III	ST335	17.54 (16.34–18.23)
2008232582	III		18.99 (18.34–19.67)
2014210283	III		19.93 (19.87–20.29)
20151912	IV	ST452	17.30 (16.53–17.93)
20155826	IV	ST459	17.41 (16.95–18.10)
20152623	IV	ST468	17.69 (17.55–17.85)
2011201884	IV		19.93 (19.90–19.98)
2014210293	IV		18.71 (18.66–18.78)
20155749	V	ST1	15.45 (15.19–15.89)
20155753	V	ST19	15.91 (15.63–16.22)
20155859	V	ST26	15.25 (15.13–15.31)
2008232731	V		18.45 (17.89–18.92)
2014210268	V		18.13 (18.09–18.17)
20156225	VI	ST1	18.66 (18.09–19.10)
20155762	VI	ST1	18.16 (17.60–18.68)
20154691	VI	ST1	17.51 (16.84–17.85)
2010228816	VI		20.98 (20.55–21.29)
2013225975	VI		21.42 (21.09–21.97)
20154086	VII	ST1	16.55 (15.88–16.96)
20154176	VII	ST1	17.11 (16.51–17.56)
4832-06	VII		19.91 (19.50–20.49)
2014201718	VII		19.45 (19.29–19.66)
20140487	VIII		16.05 (15.13–17.01)
20150287	VIII		16.06 (15.25–17.65)
2014207299	VIII		14.80 (13.89–15.56)
5030-08	VIII		18.24 (18.09–18.35)
2013226269	VIII		23.08 (22.97–23.29)
20155073	IX	ST130	16.73 (15.57–18.06)
7509-07	IX		18.36 (18.34–18.39)
2014203375	IX		17.77 (17.41–17.97)

Reported C_T_ is the mean of three technical replicates, with range given in parentheses. Serotype as determined by latex agglutination and MLST (when available) are indicated.

**Table 3:** Real-time PCR serotyping of clinical GBS isolates is concordant with latex agglutination.

Strain	Real-time PCR	Latex Agglutination
AR611	IV	IV
AR618	III	III
AR624	II	II
AR626	III	III
AR629	IV	IV
AR630	Ib	Ib
AR631	III	III
AR634	III	III
AR667	III	III
AR880	III	III
AR905	Ia	Ia
AR1019	Ia	Ia
AR1021	III	III
AR1036	Ia	Ia
AR1037	IV	IV
AR1046	III	III
AR1049	Ia	Ia
AR1053	Ia	Ia
AR1054	III	III
AR1055	III	III
AR1056	III	III

A collection of 21 clinical isolates of unknown serotypes was tested by real-time PCR and by latex agglutination. All strains gave a single serotype by real-time PCR (C_T_ < 30; range 15–23 for 50 ng template/reaction).
