# Supplementary material for: Real-time PCR-based serotyping of Streptococcus agalactiae
Source: Sci Rep. 2016 Dec 2;6:38523. doi: 10.1038/srep38523 (PMC5133537; doi:10.1038/srep38523)
Supplement: Supplementary Information S1 [file srep38523-s1.pdf]

Supplemental File
Real-time PCR-based serotyping of *Streptococcus agalactiae*
Kathleen M. Breeding, Bhavana Ragipani, Kun-Uk David Lee, Martin Malik, Tara M. Randis, and Adam J. Ratner

Multiple sequence alignment

```
10 Sequences Aligned          Alignment Score = 0
Gaps Inserted = 2958          Conserved Identities = 1416

1a-CP000114         1 TTGAAAGAAAAAGAAAATATACAAAAGATTATTATAGCGATGATTCAAACAGTTGTGGTT    60
1b-FO393392         1 TTGAAAGAAAAAGAAAATATACAAAAGATTATTATAGCGATGATTCAAACAGTTGTGGTT    60
2-AAJO01000077      1                                                                0
3-AL766849          1 TTGAAAGAAAAAGAAAATATACAAAAGATTATTATAGCGATGATTCAAACAGTTGTAGTT    60
4-AF355776          1                                        ATGATTCAAACCGTTGTGGTT    21
5-AE009948          1 TTGAAAGAAAAAGAAAATATACAAAAGATTATTATAGCGATGATTCAAACAGTTGTGGTT    60
6-HF952106          1 TTGAAAGAAAAAGAAAATATACAAAAGATTATTATAGCGATGATTCAAACAGTTGTGGTT    60
7-AY376403          1                                        ATGATTCAAACAGTTGTGGTT    21
8-AY375363          1 ATGTACAATCCAGATTCCTCAAAAAAGATT-TATTATTTGTTAT-CAGATA-TCATTGCT    57
9-CGBY01000002      1  TGAAAGAAAAAGAAAATATACAAAAGATTATTATAGCGATGATTCAAACAGTTGTAGTT    59


1a-CP000114        61 TATTTTTCTGCAAGTTTGACATTAACA----TTAATTACT--CCCAATTTTAAAAGCAAT   114
1b-FO393392        61 TATTTTTCTGCAAGTTTGACATTAACA----TTAATTACT--CCCAATTTTAAAAGCAAT   114
2-AAJO01000077      1                                                                0
3-AL766849         61 TATTTTTCTGCAAGTTTGACATTAACA----TTAATTACT--CCCAATTTTAAAAGCAAT   114
4-AF355776         22 TATTTTTCTGCAAGTTTGACATTAACA----TTAATTACT--CCCAACTTTAAAAGCAAT    75
5-AE009948         61 TATTTTTCTGCAAGTTTGACATTAACA----TTAATTACT--CCCAATTTTAAAAGCAAT   114
6-HF952106         61 TATTTTTCTGCAAGTTTGACATTAACA----TTAATTACT--CCCAATTTTAAAAGCAAT   114
7-AY376403         22 TATTTTTCTGCAAGTTTGACATTAACA----TTAATTACT--CCCAATTTTAAAAGCAAT    75
8-AY375363         58 AATTTACTAACGTATGTATTATTAGCAATTTTTTATCATTATTCTTTTTTTGAAAG---T   114
9-CGBY01000002     60 TATTTTTCTGCAAGTTTGACATTAACA----TTAATTACT--CCCAATTTTAAAAGCAAT   113


1a-CP000114       115 AAAGATTTATTGTTTGTTCTATTGATAC--ATTATATTGTCTTTTATCTTTCTGATT---   169
1b-FO393392       115 AAAGATTTATTGTTTGTTCTATTGATAC--ATTATATTGTCTTCTATCTTTCTGATT---   169
2-AAJO01000077      1                                                                0
3-AL766849        115 AAAGATTTATTGTTTGTTCTATTGATAC--ATTATATTGTCTTTTATCTTTCTGATT---   169
4-AF355776         76 AAAGATTTATTGTTTGTTCTATTGATAC--ATTATATTGTCTTTTATCTTTCTGATT---   130
5-AE009948        115 AAAGATTTATTGTTTGTTCTATTGATAC--ATTATATTGTCTTTTATCTTTCTGATT---   169
6-HF952106        115 AAAGATTTATTGTTTGTTCTATTGATAC--ATTATATTGTCTTTTATCTTTCTGATT---   169
7-AY376403         76 AAAGATTTATTGTTTGTTCTATTGATAC--ATTATATTGTCTTTTATCTTTCTGATT---   130
8-AY375363        115 TCATATTTCTTAATTATTTTTATAACACTCATTGTTGTATCATCTGTTTTTATGGATGAA   174
9-CGBY01000002    114 AAAGATTTATTGTTTGTTCTATTGATAC--ATTATATTGTCTTTTATCTTTCTGATT---   168
```

```
1a-CP000114       170 TTTACAGAGACTTTTGGAGTCGTGGCTATCTTGAAGAGTTTAAAATGGTATTGAAATACA   229
1b-FO393392       170 TTTACAGAGACTTTTGGAGTCGTGGCTATCTTGAAGAGTTTAAAATGGTATTGAAATACA   229
2-AAJO01000077      1                                                                0
3-AL766849        170 TTTACAGAGACTTTTGGAGTCGTGGCTATCTTGAAGAGTTTAAAATGGTATTGAAATACA   229
4-AF355776        131 TTTACAGAGACTTTTGGAGTCGTGGCTATCTTGAAGAGTTTAAAATGGTATTGAAATACA   190
5-AE009948        170 TTTACAGAGACTTTTGGAGTCGTGGCTATCTTGAAGAGTTTAAAATGGTATTGAAATACA   229
6-HF952106        170 TTTATAGAGACTTTTGGAGTCGTGGCTATCTTGAAGAGTTTAAAATGGTATTGAAATACA   229
7-AY376403        131 TTTACAGAGACTTTTGGAGTCGTGGCTATCTTGAAGAGTTTAAAATGGTATTGAAATACA   190
8-AY375363        175 TATACTTATATTACTAGAA--GAGGTTATTTAAAAGAACTTAAATCATCTATTATCTATG   232
9-CGBY01000002    169 TTTACAGAGATTTTTGGAGTCGTGGCTATCTTGAAGAGTTTAAAATGGTATTGAAATACA   228


1a-CP000114       230 GCTTTTACTATATTTTCATATCAAGTTCATTATTTTTTATTTTTAAAAACTCA----TTT   285
1b-FO393392       230 GCTTTTACTATATTTTCATATCAAGTTCATTATTTTTTATTTTTAAAAACTCT----TTT   285
2-AAJO01000077      1                                                                0
3-AL766849        230 GCTTTTACTATATTTTCATATCAAGTTCATTATTTTTTATTTTTAAAAACTCT----TTT   285
4-AF355776        191 GCTTTTACTATATTTTCATATCAAGTTCATTATTTTTTATTTTTAAAAACTCT----TTT   246
5-AE009948        230 GCTTTTACTATATTTTCATATCAAGTTCATTATTTTTTATTTTTAAAAACTCT----TTT   285
6-HF952106        230 GCTTTTACTATATTTTCATATCAAGTTCATTATTTTTTATTTTTAAAAACTCT----TTT   285
7-AY376403        191 GCTTTTACTATATTTTCATATCAAGTTCGTTATTTTTTATTTTTAAAAACTCA----TTT   246
8-AY375363        233 GGTTAAAGTGTATTATTTTCTT---TTCATTCGTACTTAGCCTTGGGAAATTTAGGTTTT   289
9-CGBY01000002    229 GCTTTTACTATATTTTCATATCAAGTTCATTATTTTTTATTTTTAAAAACTCT----TTT   284


1a-CP000114       286 ACAACGACACGACTTTCCTTTTTTTCTTTTA-TTGCTATGAATTCGATTTTATTGTATCT   344
1b-FO393392       286 ACAACGACACGACTTTCCTTTTTTACTTTTA-TTGCTATGAATTCGATTTTATTATATCT   344
2-AAJO01000077      1                                                                0
3-AL766849        286 ACAACGACACGACTTTCCTTTTTTACTTTTA-TTGCTATGAATTCGATTTTATTGTATCT   344
4-AF355776        247 ACAACGACACGACTTTCCTTTTTTACTTTTA-TTACTATGAATTCGATTTTATTATATCT   305
5-AE009948        286 ACAACGACACGACTTTCCTTTTTTACTTTTA-TTGCTATGAATTCGATTTTATTATATCT   344
6-HF952106        286 ACAACGACACGACTTTCCTTTTTTACTTTTA-TTGCTATGAATTCGATTTTATTATATCT   344
7-AY376403        247 ACAATGACACGACTTTCCTTTTTTCCTTTTA-TTGCTATGAATTCGATTTTATTATATCT   305
8-AY375363        290 TAAGTGATATCGCCGTTTTATCTTATTTTTTCTTATTATTATTCTTTCTTCTCAATAGTT   349
9-CGBY01000002    285 ACAACGACACGACTTTCCTTTTTTACTTTTA-TTGCTATGAATTCGATTTTATTATATCT   343


1a-CP000114       345 ATTGAATTCATTTTTAAAATATTAT---CGAAAATATTCTTACGCTAAGTTTTCACGAGA   401
1b-FO393392       345 ATTGAATTCATTTTTAAAATATTGT---CGAAAATATTCTTACGTTAAGTTTTCACGAGA   401
2-AAJO01000077      1                                                                0
3-AL766849        345 ATTGAATTCATTTTTAAAATATTAT---CGAAAATATTCTTACGCTAAGTTTTCACGAGA   401
4-AF355776        306 ATTGAATTCATTTTTAAAATATTAT---CGAAAATATTCTTACGCTAAGTTTTCACGAGA   362
5-AE009948        345 ATTGAATTCATTTTTAAAATATTAT---CGAAAATATTCTTACGCTAAGTTTTCACGAGA   401
6-HF952106        345 ATTGAATTCATTTTTAAAATATTAT---CGAAAATATTCTTACGCTAAGTTTTCACGAGA   401
```

```
7-AY376403        306 ATTGAATTCATTTTTAAAATATTAT---CGAAAATATTCTTACGCTAAGTTTTCACGAGA   362
8-AY375363        350 TATTAGTTTATACTGGTCGTATTATGCTTAAATCTATTATGAGAGGGAGAGAGGATAAGA   409
9-CGBY01000002    344 ATTGAATTCATTTTTAAAATATTAT---CGAAAATATTCTTACGCTAAGTTTTCACGAGA   400


1a-CP000114       402 TACCAAAG-TTGTTTTGATAACGAATAAGGATTCTTTATCAAAAATGACCTTTAGGAATA   460
1b-FO393392       402 TACCAAAG-TTGTTTTGATAACGAATAAGGATTCTTTATCAAAAATGACCTTTAGGAATA   460
2-AAJO01000077      1                       GAATAAGGATTCTTTATCAAAAATGACCTTTAAGAATA    38
3-AL766849        402 TACCAAAG-TTGTTTTGATAACGAATAAGGATTCTTTATCAAAAATGACCTTTAGGAATA   460
4-AF355776        363 TACCAAAG-TTGTTTTGATAACGAATAAGGATTCTTTATCAAAAATGACCTTTAGGAATA   421
5-AE009948        402 TACCAAAG-TTGTTTTGATAACGAATAAGGATTCTTTATCAAAAATGACCTTTAGGAATA   460
6-HF952106        402 TACCAAAG-TTGTTTTGATAACGAATAAGGATTCTTTATCAAAAATGACCTTTAAGAATA   460
7-AY376403        363 TACCAAAG-TTGTTTTGATAACGAATAAGGATTCTTTATCAAAAATGACCTTTAAGAATA   421
8-AY375363        410 TAAAAAAAATTCTATTGTTAACTAAT--------TTTACAGATAACAAATCTTTTGAAAA   461
9-CGBY01000002    401 TATCAAAG-TTGTTTTGATAACGAATAAGGATTCTTTATCAAAAATGACCTTTAGGAATA   459
                                             ***        ****   * **  *   **  *** *

1a-CP000114       461 AATACGACCATAATTATATTGCTGTCTGCATCTTGGACTCCTCTG---AAAAGGATTGTT   517
1b-FO393392       461 AATACGACCATAATTATATCGCTGTCTGTATCTTGGACTCCTCTG---AAAAGGATTGTT   517
2-AAJO01000077     39 AATACGACCATAATTATATCGCTGTCTGTATCTTGGACTCCTCTG---AAAAGGATTGTT    95
3-AL766849        461 AATACGACCATAATTATATCGCTGTCTGTATCTTGGACTCCTCTG---AAAAGGATTGTT   517
4-AF355776        422 AATACGACCATAATTATATCGCTGTCTGTATCTTGGATTCCTCTG---AAAAGGATTGTT   478
5-AE009948        461 AATACGACCATAATTATATCGCTGTCTGTATCTTGGATTCCTCTG---AAAAGGATTGTT   517
6-HF952106        461 AATACGACCATAATTATATCGCTGTCTGTATCTTGGACTCCTCTG---AAAAGGATTGTT   517
7-AY376403        422 AATACGACCATAATTATATCGCTGTCTGTATCTTGGACTCCTCTG---AAAAGGATTGTT   478
8-AY375363        462 AGCATTACTTCAAC---ATCATTATCAAGTTGTTGGATATGTTAGTTCAAAAG--TTGTT   516
9-CGBY01000002    460 AATACGACCATAATTATATCGCTGTCTGTATCTTGGACTCCTCTG---AAAAGGATTGTT   516
                      *  *  **   **    **   * **    * *****    *  *   *****  *****

1a-CP000114       518 ATGATT-TGAAACATAACT--CGTTAAGGA-------TAATAAACAAAGATGCTCTTACT   567
1b-FO393392       518 ATGATT-TGAAACATAACT--CATTAAGGA-------TAATAAACAAAGATGCTCTGACT   567
2-AAJO01000077     96 ATGATT-TGAAACATAACT--CGTTAAGGA-------TAATAAACAAAGATGCTCTTACT   145
3-AL766849        518 ATGATT-TGAAACATAACT--CGTTAAGGA-------TAATAAACAAAGATGCTCTTACT   567
4-AF355776        479 ATGATT-TGAAACATAACT--CGTTAAGGA-------TAATAAACAAAGATGCTCTTACT   528
5-AE009948        518 ATGATT-TGAAACATAACT--CGTTAAGGA-------TAATAAACAAAGATGCTCTTACT   567
6-HF952106        518 ATGATT-TGAAACATAACT--CGTTAAGGA-------TAATAAACAAAGATGCTCTTACT   567
7-AY376403        479 ATGATT-TGAAACATAACT--CGTTAAGGA-------TAATAAACAAAGATGCTCTTACT   528
8-AY375363        517 --GATTATAGAGTAGTACCAGTATTAAGGAATGTCTCTGATATACGAAA----TTTTATT   570
9-CGBY01000002    517 ATGATT-TGAAACATAACT--CGTTAAGGA-------TAATAAACAAAGATGCTCTTACT   566
                        **** *  *  *  **     *******       * *** ** **     * * * *

1a-CP000114       568 TCAGAGTTAACCTGCTTAACTGTTGATCAAGCTTTTATTAACATACCCATTGAATTATTT   627
1b-FO393392       568 TCAGAGTTAACCTGCTTAACTGTTGATCAAGCTTTTATTAACATACCCATTGAATTATTT   627
2-AAJO01000077    146 TCAGAGTTAACCTGCTTAACTGTTGATCAAGCTTTTATTAACATACCCATTGAATTATTT   205
```

```
3-AL766849        568 TCAGAGTTAACCTGCTTAACTGTTGATCAAGCTTTTATTAACATACCCATTGAATTATTT   627
4-AF355776        529 TCAGAGTTAACCTGCTTAACTGTTGATCAAGCTTTTATTAACATACCCATTGAATTATTT   588
5-AE009948        568 TCAGAGTTAACCTGCTTAACTGTTGATCAAGCTTTTATTAACATACCCATTGAATTATTT   627
6-HF952106        568 TCAGAGTTAACCTGCTTAACTGTTGATCAAGCTTTTATTAACATACCCATTGAATTATTT   627
7-AY376403        529 TCAGAGTTAACCTGCTTAACTGTTGATCAAGCTTTTATTAACATACCCATTGAATTATTT   588
8-AY375363        571 GCTAA--TAATCAG-------GTAGATGAGATTTTTGT--AGATATTGAT--GCTTATTC   617
9-CGBY01000002    567 TCAGAGTTAACCTGCTTAACTGTTGATCAAGCTTTTATTAACATACCCATTGAATTATTT   626
                       *  *  *** * *       ** *** *   **** *  * ***   **    *****

1a-CP000114       628 GGTAAATACCAAAT--ACAAGATATTATTAATGACATTGAAGCAATGGGAGTGATTGTCA   685
1b-FO393392       628 GGTAAATACCAAAT--ACAAGATATTATTAATGACATTGAAGCAATGGGAGTGATTGTCA   685
2-AAJO01000077    206 GGTAAATACCAAAT--ACAAGATATTATTAATGACATTGAAGCAATGGGAGTGATTGTCA   263
3-AL766849        628 GGTAAATACCAAAT--ACAAGATATTATTAATGACATTGAAGCAATGGGAGTGATTGTCA   685
4-AF355776        589 GGTAAATACCAAAT--ACAAGATATTATTAATGACATTGAAGCAATGGGAGTGATTGTCA   646
5-AE009948        628 GGTAAATACCAAAT--ACAAGATATTATTAATGACATTGAAGCAATGGGAGTGATTGTCA   685
6-HF952106        628 GGTAAATACCAAAT--ACAAGATATTATTAATGACATTGAAGCAATGGGAGTGATTGTCA   685
7-AY376403        589 GGTAAATACCAAAT--ACAAGATATTATTAATGACATTGAAGCAATGGGAGTGATTGTCA   646
8-AY375363        618 TGATTATATTGAGGCAGCACGATATTTTAAACTAC-TTGG---AATTCCGACAACTATAA   673
9-CGBY01000002    627 GGTAAATACCAAAT--ACAAGATATTATTAATGACATTGAAGCAATGGGAGTGATTGTCA   684
                       *   ***   *     ** ****** * **  ** ***    ***       * * * *

1a-CP000114       686 ATGT--TAATGTAGAGGCACT----TAGCTTTGATAATATAGGAGAAAAGCGAATCCAAA   739
1b-FO393392       686 ATGT--TAATGTAGAGGCACT----TAGCTTTGATAATATAGGAGAAAAGCTAATCCAAA   739
2-AAJO01000077    264 ATGT--TAATGTAGAGGCACT----TAGCTTTGATAATATAGGAGAAAAGCGAATCCAAA   317
3-AL766849        686 ATGT--TAATGTAGAGGCACT----TAGCTTTGATAATATAGGAGAAAAGCGAATCCAAA   739
4-AF355776        647 ATGT--TAATGTAGAGGCACT----TAGCTTTGATAATATAGGAGAAAAGCGAATCCAAA   700
5-AE009948        686 ATGT--TAATGTAGAGGCACT----TAGCTTTGATAATATAGGAGAAAAGCGAATCCAAA   739
6-HF952106        686 ATGT--TAATGTAGAGGCACT----TAGCTTTGATAATATAGGAGAAAAGCGAATCCAAA   739
7-AY376403        647 ATGT--TAATGTAGAGGCACT----TAGCTTTGATAATATAGGAGAAAAGCGAATCCAAA   700
8-AY375363        674 ATATAACAAATTATAATAACTATTATATTTATAATAGTATTTTAAAAAAAATAG------   727
9-CGBY01000002    685 ATGT--TAATGTAGAGGCACT----TAGCTTTGATAATATAGGAGAAAAGCGAATCCAAA   738
                      ** *   **  ** *   ***    **  * * *** ***   * ****   *

1a-CP000114       740 CTTTTGAAGGATATAGTGTTATTACATATTCTATGAAATTCTATAAATATAGTCACCTTA   799
1b-FO393392       740 CTTTTGAAGGATATAGTGTTATTACATATTCTATGAAATTCTATAAATGTAGTCACCTTA   799
2-AAJO01000077    318 CTTTTGAAGGATATAGTGTTATTACATATTCTATGAAATTCTATAAATATAGTCACCTTA   377
3-AL766849        740 CTTTTGAAGGATATAGTGTTATTACATATTCTATGAAATTCTATAAATATAGTCACCTTA   799
4-AF355776        701 CTTTTGAAGGATATAGTGTTATTACATATTCTATGAAATTCTATAAATATAGTCACCTTA   760
5-AE009948        740 CTTTTGAAGGATATAGTGTTATTACATATTCTATGAAATTCTATAAATATAGTCACCTTA   799
6-HF952106        740 CTTTTGAAGGATATAGTGTTATTACATATTCTATGAAATTCTATAAATATAGTCACCTTA   799
7-AY376403        701 CTTTTGAAGGATATAGTGTTATTACATATTCTATGAAATTCTATAAATATAGTCACCTTA   760
8-AY375363        728 -----GTAATATGTCAT-TTGTGACAACAGCAATTAATATCGTTAGTTTAGACAATTGT    781
9-CGBY01000002    739 CTTTTGAAGGATATAGTGTTATTACATATTCTATGAAATTCTATAAATATAGTCACCTTA   798
                           * *  ** *  * ** * ***    * ** **  **  **  * *** **  *
```

```
1a-CP000114       800 TAGCAAAACGATTTTTGGATATCACGGGTGCTATTATAGGTTTGCTCATATGTGGCATTG   859
1b-FO393392       800 TAGCAAAACGATTTTTGGATATCACAGGTGCTATTATAGGTTTGCTCATATGTGGCATTG   859
2-AAJO01000077    378 TAGCAAAACGATTTTTGGATATCATGGGTGCTATTATAGGTTTGCTCATATGTGGCATTG   437
3-AL766849        800 TAGCAAAACGATTTTTGGATATCACGGGTGCTATTATAGGTTTGCTCATATGTGGCATTG   859
4-AF355776        761 TAGCAAAACGATTTTTGGATATCACGGGTGCTATTATAGGTTTGCTCATATGTGGCATTG   820
5-AE009948        800 TAGCAAAACGATTTTTGGATATCACGGGTGCTATTATAGGTTTGCTCATATGTGGCATTG   859
6-HF952106        800 TAGCAAAACGATTTTTGGATATCACGGGTGCTATTATAGGTTTGCTCATATGTGGCATTG   859
7-AY376403        761 TAGCAAAACGATTTTTGGATATCATGGGTGCTATTATAGGTTTGCTCATATGTGGCATTG   820
8-AY375363        782 TTTTAAAGAGAATAATAGATATTTTTATAGCAGTTATTGGTATTTTTATAACAGGAATTG   841
9-CGBY01000002    799 TAGCAAAACGATTTTTGGATATCACGGGTGCTATTATAGGTTTGCTCATATGTGGCATTG   858
                      *   ***  ** *  * *****       **  **** *** *  * ***   ** ****

1a-CP000114       860 TGGCAATTTTTCTAGTTCCACAAATCAGAAAAG-ATGGT-----GGACCGGCTATCTTTT   913
1b-FO393392       860 TGGCAATTTTCCTAGTTCCGCAAATCAGAAAAG-ATGGT-----GGACCGGCTATCTTTT   913
2-AAJO01000077    438 TGGCAATTTTTCTAGTTCCGCAAATCAGAAAAG-ATGGT-----GGACCGGCTATCTTTT   491
3-AL766849        860 TGGCAATTTTTCTAGTTCCACAAATCAGAAAAG-ATGGT-----GGACCGGCTATCTTTT   913
4-AF355776        821 TGGCAATTTTTCTAGTTCCACAAATCAGAAAAG-ATGGT-----GGACCGGCTATCTTTT   874
5-AE009948        860 TGGCAATTTTTCTAGTTCCACAAATCAGAAAAG-ATGGT-----GGACCGGCTATCTTTT   913
6-HF952106        860 TGGCAATTTTTCTAGTTCCGCAAATCAGAAAAG-ATGGT-----GGACCGGCTATCTTTT   913
7-AY376403        821 TGGCAATTTTTCTAGTTCCGCAAATCAGAAAAG-ATGGT-----GGACCGGCTATCTTTT   874
8-AY375363        842 TTGCA---TTATTAATTTTTCCTATCATAAAAATACAATCACCAGGACCACTTTTTTTCA   898
9-CGBY01000002    859 TGGCAATTTTATAGTTCCGCAAATCAGAAAAG-ATGGT-----GGACCGGCTATCTTTT   912
                      * ***   **  ** **   *  **** ****  *   *     *****   * * **

1a-CP000114       914 CTCAAAATAGAGTAGGTCGTAATGGTAGGATTTTTAGATTCTATAAATTCAGATCAATGC   973
1b-FO393392       914 CTCAAAATAGAGTAGGTCGTAATGGTAGGATTTTTAGATTCTATAAATTCAGATCAATGC   973
2-AAJO01000077    492 CTCAAAATAGAGTAGGTCGTAATGGTAGGATTTTTAGATTCTATAAATTCAGATCAATGC   551
3-AL766849        914 CTCAAAATAGAGTAGGTCGTAATGGTAGGATTTTTAGATTCTATAAATTCAGATCAATGC   973
4-AF355776        875 CTCAAAATAGAGTAGGTCGTAATGGTAGGATTTTTAGATTCTATAAATTCAGATCAATGC   934
5-AE009948        914 CTCAAAATAGAGTAGGTCGTAATGGTAGGATTTTTAGATTCTATAAATTCAGATCAATGC   973
6-HF952106        914 CTCAAAATAGAGTAGGTCGTAATGGTAGGGTTTTTAGATTTTATAAATTCAGATCAATGC   973
7-AY376403        875 CTCAAAATAGAGTAGGTCGTAATGGTAGGATTTTTAGATTCTATAAATTCAGATCAATGC   934
8-AY375363        899 CGCAGAAACGTGTTGGAAAGAACGGAAAAGTATTTGAAATCTATAAATTTAGAAGTATGT   958
9-CGBY01000002    913 CTCAAAATAGAGTAGGTCGTAATGGTAGGATTTTTAGATTCTATAAATTCAGATCAATGC   972
                      * ** **  * ** **    ** ** *   * ***  * * ******** ***   ***

1a-CP000114       974 GAGTAGATGCAGAACAAATTAAGAAAGATTTATTAGTTCACAATCA----AATGACAGGG  1029
1b-FO393392       974 GAGTAGATGCAGAACAAATTAAGAAAGATTTATTAGTTCACAATCA----AATGACAGGG  1029
2-AAJO01000077    552 GAGTAGATGCAGAACAAATTAAGAAAGATTTATTAGTTCACAATCA----AATGACAGGG   607
3-AL766849        974 GAGTAGATGCAGAACAAATTAAGAAAGATTTATTAGTTCACAATCA----AATGACAGGG  1029
4-AF355776        935 GAGTAGATGCAGAACAAATTAAGAAAGATTTATTAGTTCACAATCA----AATGACAGGG   990
5-AE009948        974 GAGTAGATGCAGAACAAATTAAGAAAGATTTATTAGTTCACAATCA----AATGACAGGG  1029
6-HF952106        974 GAGTAGATGCAGAACAAATTAAGAAAGATTTATTAGTTCACAATCA----AATGACAGGG  1029
```

```
7-AY376403        935 GAGTAGATGCAGAACAAATTAAGAAAGATTTATTAGTTCACAATCA----AATGACAGGG   990
8-AY375363        959 ATATAGATGCAGAGCAGCGAAAAAAAGAATTATTATCTAAAAATAATCTTAAT-ACGAAT  1017
9-CGBY01000002    973 GAATAGATGCAGAACAAATCAAGAAAGATTTATTAGTTCACAATCA----AATGACAGGG  1028
                         ********** **    ** ***** ******  * * *** *    *** **

1a-CP000114      1030 CTAATGTTTAAGTTAGACGATGATCCTAGAATTACTAAAATAGGAAAATTTATTCGAAAA  1089
1b-FO393392      1030 CTAATGTTTAAGTTAGAAGATGATCCTAGAATTACTAAAATAGGAAAATTTATTCGAAAA  1089
2-AAJO01000077    608 CTAATGTTTAAGTTAGACGATGATCCTAGAATTACTAAAATAGGAAAATTTATTCGAAAA   667
3-AL766849       1030 CTAATGTTTAAGTTAGAAGATGATCCTAGAATTACTAAAATAGGAAAATTTATTCGAAAA  1089
4-AF355776        991 CTAATGTTTAAGTTAGAAGATGATCCTAGAATTACTAAAATAGGAAAATTTATTCGAAAA  1050
5-AE009948       1030 CTAATGTTTAAGTTAGACGATGATCCTAGAATTACTAAAATAGGAAAATTTATTCGAAAA  1089
6-HF952106       1030 CTAATGTTTAAGTTAGACGATGATCCTAGAATTACTAAAATAGGAAAATTTATTCGAAAA  1089
7-AY376403        991 CTAATGTTTAAGTTAGACGATGATCCTAGAATTACTAAAATAGGAAAATTTATTCGAAAA  1050
8-AY375363       1018 TTGATGTTTAAAATGGAAAATGATCCAAGAATTTTCCATTTGGACAAAAAATTAGAAAT  1077
9-CGBY01000002   1029 CTAATGTTTAAGTTAGAAGATGATCCTAGAATTACTAAAATAGGAAAATTTATTCGAAAA  1088
                       * ********  * **  ******* ******  *  * * *** **   *** ****

1a-CP000114      1090 ACAAGCATAGATGAGTTGCCTCAATTCTATAATGTTTTAAAAGGTGATATGAGTTTAGTA  1149
1b-FO393392      1090 ACAAGCATAGATGAGTTGCCTCAATTCTATAATGTTTTAAAAGGTGATATGAGTTTAGTA  1149
2-AAJO01000077    668 ACAAGCATAGATGAGTTGCCTCAATTCTATAATGTTTTAAAAGGTGATATGAGTTTAGTA   727
3-AL766849       1090 ACAAGCATAGATGAGTTGCCTCAATTCTATAATGTTTTAAAAGGTGATATGAGTTTAGTA  1149
4-AF355776       1051 ACAAGCATAGATGAGTTGCCTCAATTCTATAATGTTTTAAAAGGTGATATGAGTTTAGTA  1110
5-AE009948       1090 ACAAGCATAGATGAGTTGCCTCAATTCTATAATGTTTTAAAGGGTGATATGAGTTTAGTA  1149
6-HF952106       1090 ACAAGCATAGATGAGTTGCCACAATTCTATAATGTTTTAAAAGGTGATATGAGTTTAGTA  1149
7-AY376403       1051 ACAAGCATAGATGAGTTGCCTCAATTCTATAATGTTTTAAAAGGTGATATGAGTTTAGTA  1110
8-AY375363       1078 TGGTCTATTGATGAACTTCCTCAATTTTTAAACGTTTTAAAAGGAGATATGTCTGTGGTA  1137
9-CGBY01000002   1089 ACAAGCATAGATGAGTTGCCTCAATTCTATAATGTTTTAAAAGGTGATATGAGTTTAGTA  1148
                            ** *****  * ** ***** *  ** ******** ** ******  * * ***

1a-CP000114      1150 GGAACACGCCCTCCCACAGTTGATGAATATGAAAAGTATAATTCAACGCAG---AAGCGA  1206
1b-FO393392      1150 GGAACACGCCCTCCCACAGTTGATGAATATGAAAAGTATAATTCAACGCAG---AAGCGA  1206
2-AAJO01000077    728 GGAACACGCCCTCCCACAGTTGATGAATATGAAAAGTATAATTCAACGCAG---AAGCGA   784
3-AL766849       1150 GGAACACGCCCTCCCACAGTTGATGAATATGAAAAGTATAATTCAACGCAG---AAGCGA  1206
4-AF355776       1111 GGAACACGCCCTCCCACAGTTGATGAATATGAAAAGTATAATTCAACGCAG---AAGCGA  1167
5-AE009948       1150 GGAACACGCCCTCCCACAGTTGATGAATATGAAAAGTATAATTCAACGCAG---AAGCGA  1206
6-HF952106       1150 GGAACACGCCCTCCCACAGTTGATGAATATGAAAAGTATAATTCAACGCAG---AAGCGA  1206
7-AY376403       1111 GGAACACGCCCTCCCACAGTTGATGAATATGAAAAGTATAATTCAACGCAG---AAGCGA  1167
8-AY375363       1138 GGTACTCGTCCTCCGACCCTTGAGGAATATAAACAATATGAGTTACATCATTTTAAACGA  1197
9-CGBY01000002   1149 GGAACACGCCCTCCCACAGTTGATGAATATGAAAAGTATAATTCAACGCAG---AAGCGA  1205
                      ** ** ** ***** **  **** ****** ** * *** * * *  **     ** ***

1a-CP000114      1207 CGCCTTAGTT-TTAAGCCAGGAATCACTGGTTTGTGGCAAATATCTGGTAGAAATAATAT  1265
1b-FO393392      1207 CGCCTTAGTT-TTAAGCCAGGAATCACTGGTTTGTGGCAAATATCTGGTAGAAATAATAT  1265
2-AAJO01000077    785 CGCCTTAGTT-TTAAGCCAGGAATCACTGGTTTGTGGCAAATATCTGGTAGAAATAATAT   843
```

```
3-AL766849      1207 CGCCTTAGTT-TTAAGCCAGGAATCACTGGTTTGTGGCAAATATCTGGTAGAAATAATAT  1265
4-AF355776      1168 CGCCTTAGTT-TTAAGCCAGGAATCACTGGTTTGTGGCAAATATCTGGTAGAAATAATAT  1226
5-AE009948      1207 CGCCTTAGTT-TTAAGCCAGGAATCACTGGTTTGTGGCAAATATCTGGTAGAAATAATAT  1265
6-HF952106      1207 CGCCTTAGTT-TTAAGCCAGGAATCACTGGTTTGTGGCAAATATCTGGTAGAAATAATAT  1265
7-AY376403      1168 CGCCTTAGTT-TTAAGCCAGGAATCACTGGTTTGTGGCAAATATCTGGTAGAAATAATAT  1226
8-AY375363      1198 ----TTAGCTGCTAAACCAGGAATTACTGGTTTATGGCAAGTTAGTGGACGAAGTGCCAT  1253
9-CGBY01000002  1206 CGCCTTAGTT-TTAAACCAGGAATCACTGGTTTGTGGCAAATATCTGGTAGAAATAATAT  1264
                         **** *  *** ******** ******** ****** *   ***  *** *   **

1a-CP000114     1266 CACTGATTTTGATGAAATCGTAAAGTTAGATGTTCAATATATCAATGAATGGTCTATTTG  1325
1b-FO393392     1266 TACTGATTTTGATGAAATCGTAAAGTTAGATGTTCAATATATCAATGAATGGTCTGTTTG  1325
2-AAJO01000077   844 TACTGATTTTGATGAAATCGTAAAGTTAGATGTTCAATATATCAATGAATGGTCTATTTG   903
3-AL766849      1266 CACTGATTTTGATGAAATCGTAAAGTTAGATGTTCAATATATCAATGAATGGTCTATTTG  1325
4-AF355776      1227 TACTGATTTTGATGAAATCGTAAAGTTAGATGTTCAATATATCAATGAATGGTCTATTTG  1286
5-AE009948      1266 TACTGATTTTGATGAAATCGTAAAGTTAGATGTTCAATATATCAATGAATGGTCTATTTG  1325
6-HF952106      1266 TACTGATTTTGATGAAATCGTAAAGTTAGATGTTCAATATATCAATGAATGGTCTATTTG  1325
7-AY376403      1227 TACTGATTTTGATGAAATCGTAAAGTTAGATGTTCAATATATCAATGAATGGTCTATTTG  1286
8-AY375363      1254 TACTGACTTTGAGGAAGTTGTTTCATTGGATATGAAATATATTCAAAATTGGTCATTGTC  1313
9-CGBY01000002  1265 TACTGATTTTGATGAAATCGTAAAGTTAGATGTTCAATATATCAATGAATGGTCTATTTG  1324
                      ***** ***** *** * **    ** *** *  *******  *  * *****  * *

1a-CP000114     1326 GTCAGATATTAAGATTATTCTCCTAACACTAAAGGTAGTCTTACTTGGGACAGGTGCTAA  1385
1b-FO393392     1326 GTCAGATATTAAGATTATTCTCCTAACACTAAAGGTAGTCTTACTTGGGACAGGAGCTAA  1385
2-AAJO01000077   904 GTCAGATATTAAGATTATTCTCCTAACATTAAAGGTAGTCTTACTTGGGACAGGAGCTAA   963
3-AL766849      1326 GTCAGATATTAAGATTATTCTCCTAACACTAAAGGTAGTCTTACTTGGGACAGGTGCTAA  1385
4-AF355776      1287 GTCAGATATTAAGATTATTCTCCTAACACTAAAGGTAGTCTTACTTGGGACAGGTGCTAA  1346
5-AE009948      1326 GTCAGATATTAAGATTATTCTCCTAACACTAAAGGTAGTTTTACTCGGGACAGGAGCTAA  1385
6-HF952106      1326 GTCAGATATTAAGATTATTCTCCTAACACTAAAGGTAGTTTTACTCGGGACAGGAGCTAA  1385
7-AY376403      1287 GTCAGATATTAAGATTATTCTCCTAACATTAAAGGTAGTCTTACTTGGGACAGGAGCTAA  1346
8-AY375363      1314 AGAAGATATTAAGATTATTGTAAGAACATTTGTAGTAATTTTTAAAAAAGAAGGAAGTAG  1373
9-CGBY01000002  1325 GTCAGATATTAAGATTATTCTCCTAACACTAAAGGTAGTCTTACTTGGGACAGGTGCTAA  1384
                        **************** *   **** *    *** * **         ***   **

1a-CP000114     1386 GTAAAGGTAAGGTTTGAAAG---GAATATAATGAAAATTT----GTCTGGTTGGTTCAAG  1438
1b-FO393392     1386 GTAAAGGTAAGGTTTGAAAG---GAATATAATGAAAATTT----GTCTGGTTGGTTCAAG  1438
2-AAJO01000077   964 GTAAAGGTAAGGTTTGAAAG---GAATATAATGAAAATTT----GTCTGGTTGGTTCAAG  1016
3-AL766849      1386 GTAAAGGTAAGGTTTGAAAG---GAATATAATGAAAATTT----GTCTGGTTGGTTCAAG  1438
4-AF355776      1347 GTAAAGGTAAGGTTTGAAAG---GAATATAATGAAAATTT----GTCTGGTTGGTTCAAG  1399
5-AE009948      1386 GTAAAGGTAAGGTTTGAAAG---GAATATAATGAAAATTT----GTCTGGTTGGTTCAAG  1438
6-HF952106      1386 GTAAAGGTAAGGTTTGAAAG---GAATATAATGAAAATTT----GTCTGGTTGGTTCAAG  1438
7-AY376403      1347 GTAAAGGTAAGGTTTGAAAG---GAATATAATGAAAATTT----GTCTGGTTGGTTCAAG  1399
8-AY375363      1374 ATAGTGGAATTATTTACTCTCTTAAATTTTAT-AAAATTTAAGAGAATGGAGGAGTTATG  1432
9-CGBY01000002  1385 GTAAAGGTAAGGTTTGAAAG---GAATATAATGAAAATTT----GTCTGGTTGGTTCAAG  1437
                      **  ** *   ***         *** * ** *******    *  ***  *  * * *
```

```
1a-CP000114      1439 TGGTGGTCATCTAGCACACTTGAACCTTTTGAAACCCATTTGGGAAAAAGAAGATAGGTT  1498
1b-FO393392      1439 TGGTGGTCATCTAGCACACTTGAACCTTTTGAAACCCATTTTGGAAAAAGAAGATAGGTT  1498
2-AAJO01000077   1017 TGGTGGTCATCTAGCACACTTGAACCTTTTGAAACCCATTTGGGAAAAAGAAGATAGGTT  1076
3-AL766849       1439 TGGTGGTCATCTAGCACACTTGAACCTTTTGAAACCCATTTGGGAAAAAGAAGATAGGTT  1498
4-AF355776       1400 TGGTGGTCATCTAGCACACTTGAACCTTTTGAAACCCATTTTGGAAAAAGAAGATAGGTT  1459
5-AE009948       1439 TGGTGGTCATCTAGCACACTTGAACCTTTTGAAACCCATTTGGGAAAAAGAAGATAGGTT  1498
6-HF952106       1439 TGGTGGTCATCTAGCACACTTGAACCTTTTGAAACCCATTTGGGAAAAAGAAGATAGGTT  1498
7-AY376403       1400 TGGTGGTCATCTAGCACACTTGAACTTTTTGAAATCCATTTGGGAAAAAGAAGATAGGTT  1459
8-AY375363       1433 AGAAGGACA----GTATATATTATTGGTTCAAAAGGTATTCCGGCAAGATATGGTGGATT  1488
9-CGBY01000002   1438 TGGTGGTCATCTAGCACACTTGAACCTTTTGAAACCCATTTTGGAAAAAGAAGATAGGTT  1497
                       *  ** **    * * *  * *    **  ***   ***  ** ** * * * * * **

1a-CP000114      1499 TTGGGTAACCTTTGATAAAGAAGATGCTAGGAGTATTCTAAGAGAAGAGATTGTATATC-  1557
1b-FO393392      1499 TTGGGTAACCTTTGATAAAGAAGATGCTAGGAGTATTCTAAGAGAAGAGATTGTATATC-  1557
2-AAJO01000077   1077 TTGGGTAACCTTTGATAAAGAAGATGCTAGGAGTATTCTAAGAGAAGAGATTGTATATC-  1135
3-AL766849       1499 TTGGGTAACCTTTGATAAAGAAGATGCTAGGAGTATTCTAAGAGAAGAGATTGTATATC-  1557
4-AF355776       1460 TTGGGTAACCTTTGATAAAGAAGATGCTAGGAGTATTCTAAGAGAAGAGATTGTATATC-  1518
5-AE009948       1499 TTGGGTAACCTTTGATAAAGAAGATGCTAGGAGTATTCTAAGAGAAGAGATTGTATATC-  1557
6-HF952106       1499 TTGGGTAACCTTTGATAAAGAAGATGCTAGGAGTATTCTAAGAGAAGAGATTGTATATC-  1557
7-AY376403       1460 TTGGGTAACCTTTGATAAAGAAGATGCTAGGAGTATTCTAAGAGAAGAGATTGTATATC-  1518
8-AY375363       1489 T---GAAACGTTTGTTGAAAAACTTACAGAAAAGCATAAAAATAAAGACATCAAATATTT  1545
9-CGBY01000002   1498 TTGGGTAACCTTTGATAAAGAAGATGCTAGGAGTATTCTAAGAGAAGAGATTGTATATC-  1556
                      *   * *** **** * ** **  * *    *    *  **   **** **   ****

1a-CP000114      1558 -ATTGCTTCTTTCCAACAAA---CCGTAATGTCAAAAACTTGGTAAAAAATACTATTCTA  1613
1b-FO393392      1558 -ATTGCTTCTTTCCAACAAA---CCGTAATGTCAAAAACTTGGTAAAAAATACTATTCTA  1613
2-AAJO01000077   1136 -ATTGCTTCTTTCCAACAAA---CCGTAATGTCAAAAACTTGGTAAAAAATACTATTCTA  1191
3-AL766849       1558 -ATTGCTTCTTTCCAACAAA---CCGTAATGTCAAAAACTTGGTAAAAAATACTATTCTA  1613
4-AF355776       1519 -ATTGCTTCTTTCCAACAAA---CCGTAATGTCAAAAACTTGGTAAAAAATACTATTCTA  1574
5-AE009948       1558 -ATTGCTTCTTTCCAACAAA---CCGTAATGTCAAAAACTTGGTAAAAAATACTATTCTA  1613
6-HF952106       1558 -ATTGCTTCTTTCCAACAAA---CCGTAATGTCAAAAACTTGGTAAAAAATACTATTCTA  1613
7-AY376403       1519 -ATTGCTTCTTTCCAACAAA---CCGTAATGTCAAAAACTTGGTAAAAAATACTATTCTA  1574
8-AY375363       1546 TGTAGCTTGTACTAGAGAGAATTCTATAAAATCTAATATAT--CAGAAAATAT--TTTTG  1601
9-CGBY01000002   1557 -ATTGCTTCTTTCCAACAAA---CCGTAATGTCAAAAACTTGGTAAAAAATACTATTCTA  1612
                        * **** *     * * *   *  ***  ** ** *  *   * ******   ** *

1a-CP000114      1614 GCTTTTAAGGTCCTTAGAAAAGAAAGACCAGATGTTATCATATCATCTGGTGCCGCTGTA  1673
1b-FO393392      1614 GCTTTTAAGGTCCTTAGAAAAGAAAGACCAGATGTTATCATATCATCTGGTGCCGCTGTA  1673
2-AAJO01000077   1192 GCTTTTAAGGTCCTTAGAAAAGAAAGACCAGATGTTATCATATCATCTGGTGCCGCTGTA  1251
3-AL766849       1614 GCTTTTAAGGTCCTTAGAAAAGAAAGACCAGATGTTATCATATCATCTGGTGCCGCTGTA  1673
4-AF355776       1575 GCTTTTAAGGTCCTTAGAAAAGAAAGACCAGATGTTATCATATCATCTGGTGCCGCTGTA  1634
5-AE009948       1614 GCTTTTAAGGTCCTTAGAAAAGAAAGACCAGATGTTATCATATCATCTGGTGCCGCTGTA  1673
6-HF952106       1614 GCTTTTAAGGTCCTTAGAAAAGAAAGACCAGATGTTATCATATCATCTGGTGCCGCTGTA  1673
```

```
7-AY376403        1575 GCTTTTAAGGTCCTTAGAAAAGAAAGACCAGATGTTATCATATCATCTGGTGCCGCTGTA  1634
8-AY375363        1602 AATATAATGGTGCTACTTGCTTTAATGTT-GATGTT-CCAGAT-AT-TGGTTCAGCTAAA  1657
9-CGBY01000002    1613 GCTTTTAAGGTCCTTAGAAAAGAAAGACCAGATGTTATCATATCATCTGGTGCCGCTGTA  1672
                         * * * *** **         **     ******  ** ** ** **** * ***  *

1a-CP000114       1674 GCAGTACCATTCTTTTATATTGG---TAAGTTATTTGGTTGTAAGACCGTTTATATAGAG  1730
1b-FO393392       1674 GCAGTACCATTCTTTTATATTGG---TAAGTTATTTGGTTGTAAGACCGTTTATATAGAG  1730
2-AAJO01000077    1252 GCAGTACCATTCTTTTATATTGG---TAAGTTATTTGGTTGTAAGACCGTTTATATAGAG  1308
3-AL766849        1674 GCAGTACCATTCTTTTATATTGG---TAAGTTATTTGGTTGTAAGACCGTTTATATAGAG  1730
4-AF355776        1635 GCAGTACCATTTTTTTATATTGG---TAAGTTATTTGGTTGTAAGACCGTTTATATAGAG  1691
5-AE009948        1674 GCAGTACCATTCTTTTATATTGG---TAAGTTATTTGGTTGTAAGACCGTTTACATAGAG  1730
6-HF952106        1674 GCAGTACCATTCTTTTATATTGG---TAAGTTATTTGGTTGTAAGACCGTTTATATAGAG  1730
7-AY376403        1635 GCAGTACCATTCTTTTATATTGG---TAAGTTATTTGGTTGTAAGACCATTTATATAGAG  1691
8-AY375363        1658 GCGGTGCTGTA-TGATATTCTGGCTTTAAGAAAGGCGATTG-----CTATTTCTAAAAAA  1711
9-CGBY01000002    1673 GCAGTACCATTCTTTTATATTGG---TAAGTTATTTGGTTGTAAGACCGTTTATATAGAG  1729
                       ** ** *  *  *  ***  ***   ****  *   * ***     *  ***  * * *

1a-CP000114       1731 GTTTTCGACAG-GATAGATAAACCAACTTTGACAGGAAAATTAGTGTATCCTGTAACAGA  1789
1b-FO393392       1731 GTTTTCGACAG-GATAGATAAACCAACTTTGACAGGAAAATTAGTGTATCCTGTAACAGA  1789
2-AAJO01000077    1309 GTTTTCGACAG-GATAGATAAACCAACTTTGACAGGAAAATTAGTGTATCCTGTAACAGA  1367
3-AL766849        1731 GTTTTCGACAG-GATAGATAAACCAACTTTGACAGGAAAATTAGTGTATCCTGTAACAGA  1789
4-AF355776        1692 GTTTTCGACAG-GATAGATAAACCAACTTTGACAGGAAAATTAGTGTATCCTGTAACAGA  1750
5-AE009948        1731 GTTTTCGACAG-GATGGATAAACCAACTTTGACAGGAAAATTAGTGTATCCTGTAACAGA  1789
6-HF952106        1731 GTTTTCGACAG-GATGGATAAACCAACTTTGACAGGAAAATTAGTGTATCCTGTAACAGA  1789
7-AY376403        1692 GTTTTCGACAG-GATAGATAAACCAACTTTGACAGGAAAATTAGTGTATCCTGTAACAGA  1750
8-AY375363        1712 A------ACAATGATGAAAATCCCATATTTTACA----TTTTAGCTTGTCGTAT---AGG  1758
9-CGBY01000002    1730 GTTTTCGACAG-GATAGATAAACCAACTTTGACAGGAAAATTAGTGTATCCTGTAACAGA  1788
                              ***  ***  * *  ***  *** ***      ****  * ** * *   **

1a-CP000114       1790 TAAATTTATTGTTCAGTGGGAAGAAATGAAAAAAGTTTATCCTAAGGCAATTAATTTAGG  1849
1b-FO393392       1790 TAAATTTATTGTTCAGTGGGAAGAAATGAAAAAAGTTTATCCTAAGGCAATTAATTTAGG  1849
2-AAJO01000077    1368 TAAATTTATTGTTCAGTGGGAAGAAATGAAAAAAGTTTATCCTAAGGCAATTAATTTAGG  1427
3-AL766849        1790 TAAATTTATTGTTCAGTGGGAAGAAATGAAAAAAGTTTATCCTAAGGCAATTAATTTAGG  1849
4-AF355776        1751 TAAATTTATTGTTCAGTGGGAAGAAATGAAAAAAGTTTATCCTAAGGCAATTAATTTAGG  1810
5-AE009948        1790 TAAATTTATTGTTCAGTGGGAAGAAATGAAAAAAGTTTATCCTAAGGCAATTAATTTAGG  1849
6-HF952106        1790 TAAATTTATTGTTCAGTGGGAAGAAATGAAAAAAGTTTATCCTAAGGCAATTAATTTAGG  1849
7-AY376403        1751 TAAATTTATTGTTCAGTGGGAAGAAATGAAAAAAGTTTATCCTAAGGCAATTAATTTAGG  1810
8-AY375363        1759 TCCTTTTATATCTAAATACGAT------AAAAAAATTCGTC-------AAATAG---GGG  1802
9-CGBY01000002    1789 TAAATTTATTGTTCAGTGGGAAGAAATGAAAAAAGTTTATCCTAAGGCAATTAATTTAGG  1848
                       *   *****   * * *  **       ****** **  **       ** **     **

1a-CP000114       1850 AGGAATTTTTTAATGATTT---TTGTCAC-AGTGGGGACACATGAAC---AGCAGTTCAA  1902
1b-FO393392       1850 AGGAATTTTTTAATGATTT---TTGTCAC-AGTGGGGACACATGAAC---AGCAGTTCAA  1902
2-AAJO01000077    1428 AGGAATTTTTTAATGATTT---TTGTCAC-AGTGGGGACACATGAAC---AGCAGTTCAA  1480
```

```
3-AL766849       1850 AGGAATTTTTTAATGATTT---TTGTCAC-AGTGGGGACACATGAAC---AGCAGTTCAA  1902
4-AF355776       1811 AGGAATTTTTTAATGATTT---TTGTCAC-AGTGGGGACACATGAAC---AGCAGTTCAA  1863
5-AE009948       1850 AGGAATTTTTTAATGATTT---TTGTCAC-AGTGGGGACACATGAAC---AGCAGTTCAA  1902
6-HF952106       1850 AGGAATTTTTTAATGATTT---TTGTCAC-AGTGGGGACACATGAAC---AGCAGTTCAA  1902
7-AY376403       1811 AGGAATTTTTTAATGATTT---TTGTCAC-AGTGGGGACACATGAAC---AGCAGTTCAA  1863
8-AY375363       1803 GACAACTTTTCGTTAATCCAGATGGGCATGAGTGGTTACGTGAAAAATGGAGTATTCCTG  1862
9-CGBY01000002   1849 AGGAATTTTTTAATGATTT---TTGTCAC-AGTGGGGACACATGAAC---AGCAGTTCAA  1901
                         ** ****   * **     * * **  *****  **     **    ** * * *

1a-CP000114      1903 C-CGTC-TTATTAAAGAAGTT--GATAGATTAAAAGGGACAGGTGCTATTGATCAAGAAG  1958
1b-FO393392      1903 C-CGTC-TTATTAAAGAAGTT--GATAGATTAAAAGGGACAGGTGCTATTGATCAAGAAG  1958
2-AAJO01000077   1481 C-CGTC-TTATTAAAGAAGTT--GATAGATTAAAAGGGACAGGTGCTATTGATCAAGAAG  1536
3-AL766849       1903 C-CGTC-TTATTAAAGAAGTT--GATAGATTAAAAGGGACAGGTGCTATTGATCAAGAAG  1958
4-AF355776       1864 C-CGTC-TTATTAAAGAAGTT--GATAGATTAAAAGGGACAGATGCTATTGATCAAGAAG  1919
5-AE009948       1903 C-CGTC-TTATTAAAGAAGTT--GATAGATTAAAAGGGACAGGTGCTATTGATCAAGAAG  1958
6-HF952106       1903 C-CGTC-TTATTAAAGAAGTT--GATAGATTAAAAGGGACAGGTGCTATCGATCAAGAAG  1958
7-AY376403       1864 C-CGTC-TTATTAAAGAAGTT--GATAGATTAAAAGGGACAGGTGCTATTGATCAAGAAG  1919
8-AY375363       1863 TACGTCGCTATTGGAGAATTTCAGAAAGACTAATGGTTAAGTATGCTA---------ACT  1913
9-CGBY01000002   1902 C-CGTC-TTATTAAAGAAGTT--GATAGATTAAAAGGGACAGGTGCTATTGATCAAGAAG  1957
                        ****  ****  **** **  ** *** ***  *  *    *****         *

1a-CP000114      1959 TGTTCATTCAAACGGGTTACTCAGACTTTGAACCTCAGAATTGTCAGTGGTCAAAATTTC  2018
1b-FO393392      1959 TGTTCATTCAAACGGGTTACTCAGACTTTGAACCTCAGAATTGTCAGTGGTCAAAATTTC  2018
2-AAJO01000077   1537 TGTTCATTCAAACGGGTTACTCAGACTTTGAACCTCAGAATTGTCAGTGGTCAAAATTTC  1596
3-AL766849       1959 TGTTCATTCAAACGGGTTACTCAGACTTTGAACCTCAGAATTGTCAGTGGTCAAAATTTC  2018
4-AF355776       1920 TGTTCATTCAAACGGGTTACTCAGACTTTGAACCTCAGAATTGTCAGTGGTCAAAATTTC  1979
5-AE009948       1959 TGTTCATTCAAACGGGTTACTCAGACTTTGAACCTCAGAATTGTCAGTGGTCAAAATTTC  2018
6-HF952106       1959 TGTTCATTCAAACGGGTTACTCAGACTTTGAACCTCAGAATTGTCAGTGGTCAAAATTTC  2018
7-AY376403       1920 TGTTCATTCAAACGGGTTACTCAGACTTTGAACCTCAGAATTGTCAGTGGTCAAAATTTC  1979
8-AY375363       1914 TGCTAATTTGTGATAGTAA---AAATATTGAA------AAGTAT----ATTCAAAAAGAC  1960
9-CGBY01000002   1958 TGTTCATTCAAACGGGTTACTCAGACTTTGAACCTCAGAATTGTCAGTGGTCAAAATTTC  2017
                      ** * ***       ** *   * *  *****      ** * *      ******   *

1a-CP000114      2019 TCTCATATGATGATATGAACTCTTACATGAAAGAAGCTGAGATTGTTATCACACATGG--  2076
1b-FO393392      2019 TCTCATATGATGATATGAACTCTTACATGAAAGAAGCTGAGATTGTTATCACACATGG--  2076
2-AAJO01000077   1597 TCTCATATGATGATATGAACTCTTACATGAAAGAAGCTGAGATTGTTATCACACATGG--  1654
3-AL766849       2019 TCTCATATGATGATATGAACTCTTACATGAAAGAAGCTGAGATTGTTATCACACATGG--  2076
4-AF355776       1980 TCTCATATGATGATATGAACTCTTACATGAAAGAAGCTGAGATTGTTATCACACATGG--  2037
5-AE009948       2019 TCTCATATGATGATATGAACTCTTACATGAAAGAAGCTGAGATTGTTATCACACATGG--  2076
6-HF952106       2019 TCTCATATGATGATATGAACTCTTACATGAAAGAAGCTGAGATTGTTATCACACATGG--  2076
7-AY376403       1980 TCTCATATGATGATATGAACTGTTACATGAGAGAAGCTGAAATCGTTATTACACATGG--  2037
8-AY375363       1961 TATAGTA----AATATG--CTCCTA----AAACAACCTA-------CATCGCCTATGGGA  2003
9-CGBY01000002   2018 TCTCATATGATGATATGAACTCTTACATGAAAGAAGCTGAGATTGTTATCACACATGG--  2075
                      * *  **     *****  **  **    * * ** **         **  *  ****
```

```
1a-CP000114       2077 -CGGTCCAGCGACGTTTATGAATGCAGTTTCTAAAGGGAAAAAAACTATTGTGGTTCCTA  2135
1b-FO393392       2077 -CGGCCCAGCGACGTTTATGAATGCAGTTTCTAAAGGGAAAAAAACTATTGTGGTTCCTA  2135
2-AAJO01000077    1655 -CGGCCCAGCGATGTTTATGAATGCAGTTTCTAAAGGAAAAAAAACTATTGTGGCTCCTA  1713
3-AL766849        2077 -CGGTCCAGCGACGTTTATGTCAGTTATTTCTTTAGGGAAATTACCAGTCGTTGTTCCTA  2135
4-AF355776        2038 -CGGTCCAGCGACGTTTATGAATGCAGTTTCTAAAGGGAAAAAAACTATTGTGGTTCCTA  2096
5-AE009948        2077 -CGGCCCAGCGACGTTTATGAATGCAGTTTCTAAAGGAAAAAAAACTATTGTGGTTCCTA  2135
6-HF952106        2077 -CGGTCCAGCGACGTTTATGTCAGTTATTTCTTTAGGGAAATTACCAGTTGTTGTTCCCA  2135
7-AY376403        2038 -CGGTCCAGCGACGTTTATGAATGCAGTTTCTAAGGGGAAAAAAACCATAGTAGTTCCCA  2096
8-AY375363        2004 CTGATCTGACAAAGTCAA--AACACAATTTTAAGGACAAGGTTGTTCGAAGTTGGTTTGA  2061
9-CGBY01000002    2076 -CGGTCCAGCGACGTTTATGAATGCAGTTTCTAAAGAGAAAAAAACTATTGTGGTTCCTA  2134
                         *  *   * * **  *         ***        *           ** * *   *

1a-CP000114       2136 -GACAAGAACAGTTTGGAGAGCA--TGTGAATAATCATCAGGTG-GATT-TTTTGA----  2186
1b-FO393392       2136 -GACAAGAACAGTTTGGAGAGCA--TGTGAATAATCATCAGGTG-GATT-TTTTGA----  2186
2-AAJO01000077    1714 -GACAAGAACAGTTTGGAGAGCA--TGTGAATAATCATCAGGTGGATTT-CGTTAATAAG  1769
3-AL766849        2136 -GGAGAAAGCAGTTTGGTGAACA--TATCAATGATCATCAAATACAATT-TTTTAA----  2187
4-AF355776        2097 -GACAAGAACAGTTTGGAGAGCA--TGTGAATAATCATCAGGTGGATTT-TGTTAATAAG  2152
5-AE009948        2136 -GACAAGAACAGTTTGGAGAGCA--TGTGAATAATCATCAGGTGGACTT-TGTTAATAAG  2191
6-HF952106        2136 -GGAGAAAGCAGTTTGGTGAACA--TATCAATGATCATCAAATACAATT-T-TTAA----  2186
7-AY376403        2097 -GACAAGAACAGTTTGGAGAGCA--TGTGAATAATCATCAGGTAGATTT-TGTTAATAAG  2152
8-AY375363        2062 TGATAAAAAAATTTCAGAAAATAATTATTATTTAGTAGTTGGTAGATTTGTGCCCG----  2117
9-CGBY01000002    2135 -GACAAGAACAGTTTGGAGAGCA--TGTGAATAATCATCAAGTGGATTT-TGTTAATAAG  2190
                        *   * *  * **  *  *  *  * * * * *  *     *    **

1a-CP000114       2187 ----AAGAGTTATTC--TTGAAATATGAAT-TAGAT--TATA---TTT-TGAATATCA--  2231
1b-FO393392       2187 ----AAGAGTTATTC--TTGAAATATGAAT-TAGAT--TATA---TTT-TGAATATCA--  2231
2-AAJO01000077    1770 GTAAAAACAATGTATAATTTTGATATCGTTGTAGATATTGAAAGGTTACAAAATGTAGTC  1829
3-AL766849        2188 ----AAAAATTGCCCACCTG---TATCCCT-TGGCT--TGGA---TTG-AAGATGTAG--  2231
4-AF355776        2153 GTAAAAACAATGTATAATTTTGATATCGTTGTAGATATTGAAAGGTTACAAAATGTAGTC  2212
5-AE009948        2192 GTAAAAACAATGTATAATTTTGATATCGTTGTAGATATTGAAAGGTTACAAAATGTAGTC  2251
6-HF952106        2187 ---AAAAAATTGCCCACCT---GTATCCCT-TGGCT--TGGA---TTG-AAGATGTAG--  2231
7-AY376403        2153 GTCAAGTTGATCTATAATTTTGATATTATTATAGACATTGAAGGGTTACAAAATCTAGTT  2212
8-AY375363        2118 ---AAAATAAT-TATGAGTCAATGAT--TCGCGAAT---------TTATGAAATCTAAAT  2162
9-CGBY01000002    2191 GTAAAAACAATGTATAATTTTGATATCGTTGTAGATATTGAAAGGTTACAAAATGTAGTC  2250
                           *     *       *     **                   **     ** *

1a-CP000114       2232 -GTGA----------------------------ATTAGAGAATATTA------TTAAGG-  2255
1b-FO393392       2232 -GTGA----------------------------ATTAGAGAATATTA------TTAAGG-  2255
2-AAJO01000077    1830 TATGAGGGGACGATGAATCGTCCGTTTTTAGAAACTAACAGA-AGTAATTTTATTGAAGA  1888
3-AL766849        2232 -ATGG----------------------------ACTTGCGGA-AGTG------TTGA---  2252
4-AF355776        2213 TATGAGGGGACGATGAATCGTCCGTTTTTAGAAACTAACAGA-AGTAATTTTATTGAAGA  2271
5-AE009948        2252 TATGAGGGAATGATGAATCGTCCGTTTTTAGAAACTAATAGT-AGTAATTTTATTGAAGA  2310
6-HF952106        2232 -ATGG----------------------------ACTTGCGGA-AGCG------TTGAA--  2253
```

```
7-AY376403       2213 TATGAAGGAGCGATGAATCGTCCGTTTTTAGAAACCAACAAT-AGTAATTTTATTAAAGA  2271
8-AY375363       2163 CAAAAAGAGAT-----------------------CTTGTGTTGATTACTAATATTGAGC-  2198
9-CGBY01000002   2251 TATGAGGGGACGATGAATCGTCCGTTTTTAGAAACTAACAGA-AGTAATTTTATTGAAGA  2309
                                                                 *         ** *

1a-CP000114      2256 ----AAAAAAATAT------ATCTACTAGTAAAGTAATATCAC-A-AAACAA--------  2295
1b-FO393392      2256 ----AAAAAAATAT------ATCTATTAGTAAAGTAATATCAC-A-AAACAA--------  2295
2-AAJO01000077   1889 ATTTAAGGTAATATTAAAGGAGTTGTGT---GATGAAAATCAATAAAAATAAAAATTATT  1945
3-AL766849       2253 ----AAAGGAATAT------AGCTAC----AGAAAAATATCAG-GGAAATAA--------  2289
4-AF355776       2272 ATTTAAGGTAATATTAAAGGAGTTGTGT---GATGAAAATCAATAAAAACTC------TT  2322
5-AE009948       2311 ATTTAAGGTAATATTAAAGGAGTTGTGC---GATGAAAATCAATAAAAACTC------TT  2361
6-HF952106       2254 ----AAGG-AATAT------AGCTACA----GAAAAATATCAG-GGAAATAA--------  2289
7-AY376403       2272 ATTTAAGAAGATATTAAAGGAGTTGTGT---GATGAAAATCAATAAAAACTC------TT  2322
8-AY375363       2199 ----AAAATGTTTT--------TTATG-------AGAAATTAAAAAAAGAAA--------  2231
9-CGBY01000002   2310 ATTTAAGGTAATATTAAAGGAGTTGTGC---GATGAAAATCAATAAAAACTC------TT  2360
                          **     * *         *            * ** *    **

1a-CP000114      2296 ----TGATTTTTGTTCCTCTTTCAAAAATGAACTTTCTAAACTATTTGAAT---------  2342
1b-FO393392      2296 ----TGATTTTTGTTCCTCTTTCAAGAATGAACTTTCTAAACTATTTGAAT---------  2342
2-AAJO01000077   1946 TTTTTGAAGTAA---TTTTTATCATACTT---TTTTCTCTATTA---GCTC---------  1987
3-AL766849       2290 ----TGATATGT-----TTTGTCA-------------TAAATTA---GAAA---------  2315
4-AF355776       2323 TATTTTATATTGCAATATTTTTAGTTAAT---TTTTTTAAATCACTAGGTTTAGGAGAGG  2379
5-AE009948       2362 TATTTTATATTGCAATATTTTTAGTTAAT---TTTTTTAAATCACTGGGTTTAGGCGAGG  2418
6-HF952106       2290 ----TGATATG-----TTTTGTCATAA-------------ATTA---GAAA---------  2315
7-AY376403       2323 TATTTTATATTGCAATATTTTTAGTTAAT---TTTTTTAAATCACTAGGTTTAGGAGAGG  2379
8-AY375363       2232 ----CAAAAT-------TTGATAA--------------AGATAATCGAATT---------  2257
9-CGBY01000002   2361 TATTTTATATTGCAATATTTTTAGTTAAT---TTTTTTAAATCACTAGGTTTAGGAGAGG  2417
                            *  *       *   *                  *  *

1a-CP000114      2343 -AAATATATTTTGTTG---------------------------GAG---AA---------  2362
1b-FO393392      2343 -AAATATATTTTGTTG---------------------------GAG---AA---------  2362
2-AAJO01000077   1988 -TATA------TACAG---------------------------GTGTTTAC---------  2004
3-AL766849       2316 -AAAT------TATAG---------------------------GTG---AA---------  2329
4-AF355776       2380 GGAACTCAACTTACAAAATAGTGATGTTTGTTGCAATCTTCTTGTGTGGAATAAAATTTT  2439
5-AE009948       2419 GAAACTCAGCTTACAAAATAGTGATGTTAGTTGCAATTTTACTGTGTGGAATAAAATTTT  2478
6-HF952106       2316 -AAAT------TATAG---------------------------GTG---AA---------  2329
7-AY376403       2380 AAAATTCAACTTACAAAATAGTAATGTTAGTTGCAATCTTATTGTGTGCAATAAAATTTT  2439
8-AY375363       2258 -AAAT-----TTGTTG-----------------------------GAACAGT--------  2274
9-CGBY01000002   2418 GGAACTCAACTTACAAAATAGTGATGTTAGTTGCAATCTTCTTGTGTGGAATAAAATTTT  2477
                        *        *                                 *   *

1a-CP000114      2363 ---------------------AAAAATTGAAATT-------AACTATC-------AATCC  2387
1b-FO393392      2363 ---------------------AAAAATTGAAATT-------AACTATC-------AATCC  2387
2-AAJO01000077   2005 ---------------------AGGGACGGAAATT-------ATCTT---------TATGA  2027
```

```
3-AL766849       2330 ---------------------ATATGAGGAAAT--------ATCTA---------GATTT  2351
4-AF355776       2440 TATTAGATAGCCTTTATTTTGAAAGAAGAAAACTCGTTATCATCTTTTTATTATTTATTG  2499
5-AE009948       2479 TATTAGATAGCCTTTATTTTGAAAGAAGAAAACTCGTGATCATCTTTTTATTATTTATCG  2538
6-HF952106       2330 ---------------------ATATGAGGAAAT--------ATCTA---------GATTT  2351
7-AY376403       2440 TACTCGATAGCCTTTATTTTGAAAGAAGAAAACTCGTTACTATCTTCTTGTTATTTGTCG  2499
8-AY375363       2275 -------------------TTACAATCAGCAACT--------TCTGAGCTATATTCGTGA  2307
9-CGBY01000002   2478 TATTAGATAGCCTTTATTTTGAAAGAAGAAAACTCGTTATCATCTTTTTATTATTTATCG  2537
                                           *        **           **            *

1a-CP000114      2388 AAAGTATTTG----TTAATAGGAGGAATTTTCG----------------------CTTTA  2421
1b-FO393392      2388 AAAGTATTTG----TTAATAGGAGGAATTTTCG----------------------CTTTA  2421
2-AAJO01000077   2028 CTATCTTTTT----TTACTTTATGCTTTTTATGCATTTCTGAGGTCAATCACAATTACTT  2083
3-AL766849       2352 AGATTATTCT----TTATTTTATGCTCTTTGGG----------------------TACTT  2385
4-AF355776       2500 CGACCATTTTGAATTTATTCTTTGTTCATAAGGTTACTTTTATATTAACTTTAA-TTTTT  2558
5-AE009948       2539 CGACCATTTTGAATTTATTCTTTGTTCATAAGGTTACTTTTATATTAACTTTAA-TTTTT  2597
6-HF952106       2352 AGATTATTCT----TTATTTTATGCTCTTTGGG----------------------TACTT  2385
7-AY376403       2500 CGACTATTTTGAATTTATTTTTGTACATAAGGTTACTTTTATATTAACTTTAA-TTTTT  2558
8-AY375363       2308 AAATGCCTTTGCA-TACCTTCATGGTCATGAAGT---------------TGGAGGGACTA  2351
9-CGBY01000002   2538 CGACTATTTTGAATTTATTCTTTGTTCATAAAGTTACTTTTATATTAACTTTAC-TTTTT  2596
                        *    *      *   *    *    *   *                         *

1a-CP000114      2422 ACCC--TATTTTCAAAGCCAATG----------------CAACTTTTGTT----------  2453
1b-FO393392      2422 ACCC--TATTTTCAAAGTCAATG----------------CAACTTTTGTT----------  2453
2-AAJO01000077   2084 ATTC--TAGAAAAGAA-ATAATAATTTTAATAAGTGTAACAATTCTTTTTGTTCTA----  2136
3-AL766849       2386 ATTT--TAGTACCAAA-CCAATGG------------TATCAGTTTTA------------  2418
4-AF355776       2559 TTTC--TAGCATTAAAGGATATCTCTCTA------AAAAAAGCTTTCTCTATAATAATAG  2610
5-AE009948       2598 TTTC--TAGCATTAAAGGACATCTCTCTA------AAAAAAGCTTTCTCTATAATAATAG  2649
6-HF952106       2386 ATTT--TAGTACCAAA-CCAATG------------GTATCAGTTTTT-------------  2417
7-AY376403       2559 TCCC--TAGCATTGAAGGATATCTCTCTA------AAAAAAGCTTTCTCTATAATAATAG  2610
8-AY375363       2352 ACCCATCGTTGTTAGAAGCACTGGCTTCAACGA--GTATAAATTT---------------  2394
9-CGBY01000002   2597 TTTC--TAGCATTAAAGGATATCTCTCTA------AAAAAAGCTTTCTCTATAATAATAG  2648
                                     *     *                  *  *

1a-CP000114      2454 -ACTTTTAGCATT--------AATAGTT------TTACTTA-------TTTGTAGTAGTT  2491
1b-FO393392      2454 -ACTTTTAGCATT--------AATAGTT------TTACTTA-------TTTGTAGTAGTT  2491
2-AAJO01000077   2137 AATAGTTTTTTTTCAAAAGGTAATAGTTGGCTTTCTATTT----------TGATAATATT  2186
3-AL766849       2419 -ATTATTACTATT--------A-TAGTT------CTATT------------------ATT  2444
4-AF355776       2611 GATCGCGTATTTT--------GGGAGTT------CTATTAAATCAAATTTTTGTGAAATT  2656
5-AE009948       2650 GATCGCGTATTTT--------GGGAGTT------CTATTAAATCAAATTTTTGTGAAATT  2695
6-HF952106       2418 AATTATTACCATT---------ATAGTT------CTATT------------------ATT  2444
7-AY376403       2611 GATCGCGTATATT--------GGGAGTT------CTATTAAATCAAATTTTTGTGAAATT  2656
8-AY375363       2395 -ATTATTAGATGT----------CAATT------TTAATC------------GAGAAGTT  2425
9-CGBY01000002   2649 GATCGCGTATTTT--------GGGAGTT------CTATTAAATCAAATTTTTGTGAAATT  2694
                       *          *           * **       ** *                   **
```

```
1a-CP000114      2492 A--TAATGAAAAAATGAAAT-----TTTTAAATATGGCTGAAAT---TTTTTTCATTG--  2539
1b-FO393392      2492 A--TAATGAAAAAATGAAAT-----TTTTAAATATGGCTGAAAT---TTTTTTCATTG--  2539
2-AAJO01000077   2187 A--TTAGGGAGTAGGAAGATAGCTATTGATAGGAT-TATAGATA---GTTTATTAATATG  2240
3-AL766849       2445 A--CTTTGGAAGAGTGAG-------TTTAGAATATCTATAAGCA---ATTCTTCAATAC-  2491
4-AF355776       2657 AGATTTAATAGAAATTAAATA-----TATCAATTTTTATAGGGATGGACAATTTATTCTG  2711
5-AE009948       2696 AGATTTAATAGAAATTAAGTA-----TGTCAATTTTTATAGGGATGGACAATTTATTCTG  2750
6-HF952106       2445 A--CTTTGGAAGAGTGAG-------TTTAGAATATCTATAAGCA---ATTCTTCAATAC-  2491
7-AY376403       2657 AGATTTTGTAGAAATAAAGTA-----TGTCAATTTTTATAGAGATGGACATTTCATTCTG  2711
8-AY375363       2426 G------GAGGAAATGG----------TGCATTATATTGGAAAAAAGATACTTTAAACGA  2469
9-CGBY01000002   2695 AGATTTAATAGAAATTAAGTA-----TGTCAATTTTTATAGGGATGGACAATTTATTCTG  2749
                                  *                 *   *                 * *

1a-CP000114      2540 ------TATTT-TATATTATTTATTT----AACT-TC--------AATATT---------  2570
1b-FO393392      2540 ------TATTT-TATATGGTTTATTT----AG-TATC--------AATAGT---------  2570
2-AAJO01000077   2241 TAAGATTATTTCATTTATAGGCGTTTTAACATTTGTAAACCTTCATATTTTAGAAAATAA  2300
3-AL766849       2492 ------TATTTCTGCTTTGGTTATTT----ATTTATT--------TAT-TT---------  2523
4-AF355776       2712 AGAAGTGACTTAGGTTTTGGTCATCCTAA-CTTTATTCAT-----AATTTTTT------T  2759
5-AE009948       2751 AGAAGTGACTTAGGTTTTGGTCATCCTAA-CTTTATTCAT-----AATTTTTT----TGC  2800
6-HF952106       2492 ------TATTTCTGCTTTGGTTATTT----ATTTATT--------TATTT----------  2523
7-AY376403       2712 AGAAGTGACTTAGGTTTTGGGCATCCCAA-CTTTATTCAT-----AATTTTTT----TGC  2761
8-AY375363       2470 AG----TAATTAAGCGTTGTGAACTG------TTGTC-----------------------  2496
9-CGBY01000002   2750 AGAAGTGACTTAGGTTTTGGCCATCCTAA-CTTTATTCAT-----AATTTTTT----TGC  2799
                             * **                      * *

1a-CP000114      2571 GCTA-CATTCTTTGTTTAAA-------------------AATCCTGATTTTGATAGAA--  2608
1b-FO393392      2571 ATTA-AATTCGTTATTTAGA-------------------AGTCCAGAATTTCATAGAG--  2608
2-AAJO01000077   2301 GCAAGTTCTCACA-TATAGATATGGTGAAGTAGTAGCAAGGTATGCATATGGATTTAATC  2359
3-AL766849       2524 GCAA-TACTC----ATTAGA-------------------GGTACTCAAGAGGATATAA-C  2558
4-AF355776       2760 GCAGTAACTGTTTTTTTATAT-------------------GTA-ACACTTTTTTATAGAA  2799
5-AE009948       2801 TCTAACTATTTTCTTGTATAT------------------TGTACTCAAT----TATAAAC  2838
6-HF952106       2524 GCAA-TACTCAT----TAGA-------------------GGTACTCAAGAGGATATAA-C  2558
7-AY376403       2762 TCTAACCTTTTTTTTGTATAT------------------TGTACTCAAT----TATAAAC  2799
8-AY375363       2497 -TAGAGATTCT--ATTGAGA-------------------AGTTAGCAAAACAATCGAAAA  2534
9-CGBY01000002   2800 TCTAACCATTTTCTTGTATAT------------------TGTACTCAAT----TATAAAC  2837
                              *        * *                     *    *      *  *

1a-CP000114      2609 -TTTTA----------------------------------GCAGCTTTT-----------  2622
1b-FO393392      2609 -TCATT----------------------------------GCTGCATTC-----------  2622
2-AAJO01000077   2360 ATCCCAATACCTTACATGCCTTTTTCTTTATAATAATAATGCTATTTATCTACCGTTTCT  2419
3-AL766849       2559 GTTTCA----------------------------------GCGATTTAT-----------  2573
4-AF355776       2800 AACTAAG---------------------------------ATTAATAAC-----------  2815
5-AE009948       2839 GACTAAA---------------------------------GCCTGTTGT-----------  2854
6-HF952106       2559 GTTTCA----------------------------------GCGATTTAT-----------  2573
```

```
7-AY376403       2800 GACTGAA---------------------------------GCTTATTGT-----------  2815
8-AY375363       2535 A---------------------------------------ACAGGTAAGA----------  2545
9-CGBY01000002   2838 GACTAAA---------------------------------GCTTGTTGT-----------  2853


1a-CP000114      2623 -AACT----CG------------TTGA---------TTATCGGTA---TAGTAT------  2647
1b-FO393392      2623 -AATT----CA------------CTGG-------------CAGTA---GGGGTTGTGT--  2647
2-AAJO01000077   2420 TTACTAAATTAAAGTATCTTCATTTAGTAATCATTTTAATTATTAA-TCAGTATATTTAT  2478
3-AL766849       2574 -AGCTGAGCTA------------TTAAAA------CTAATTAGTA---CAGGATATG---  2608
4-AF355776       2816 -TATTGCTTTTA---------TTTTAACT-CTAAATTACTTCTTGTATCAGTATACTTAT  2864
5-AE009948       2855 -GATGGTTTTA---------TTTTTAACA-TTAAATTATTTATTGTACCAATATACTTTT  2903
6-HF952106       2574 -TGCTGAGCTA------------TTAAAA------CTAATTAGTA---CAGGATAT----  2607
7-AY376403       2816 -AATAGTTTTA---------TTTTTAACA-TTAAATTATTTACTGTATCAATATACTTTT  2864
8-AY375363       2546 -AAATATTTTAG-----------TTGGGA---------CTTAATA-------ATA-----  2572
9-CGBY01000002   2854 -GATGGTTTTA---------TTTTTAACA-TTAAATTATTTACTGTACCAATATACTTTT  2902
                                              *                  *         *

1a-CP000114      2648 -------------CA--------TTGGCTTTTTATCAT------------TATTATA---  2671
1b-FO393392      2648 -------------CC--------TTATTATTTTACCAT------------TACTATA---  2671
2-AAJO01000077   2479 TCTATTTCAGTTGCTAGAACAGGTTATTTTTTAGTAATATTTGCAGTAG-TATTTTACAT  2537
3-AL766849       2609 -------------CT--------TTATTTTTTTATAAT------------TATTATA---  2632
4-AF355776       2865 T------------CAAGAACTGGATATTATATAGTACTCTTATTTATACTTATTATA---  2909
5-AE009948       2904 T------------CAAGGACAGGGTATTATATCGTAATTTTATT------TATTGTACTC  2945
6-HF952106       2608 ------------GCT--------TTATTTTTTTATAAT------------TATTATA---  2632
7-AY376403       2865 T------------CAAGAACAGGGTATTATATTGTAATTTTATT------TATTGTACTC  2906
8-AY375363       2573 ----------------------GATGCTTATGAGAAGT------------TATTCTA---  2595
9-CGBY01000002   2903 T------------CAAGAACAGGGTATTATATTGTAATTTTATT------TATTGTACTC  2944
                                              *            *            ** * **

1a-CP000114      2672 --------AG--AA--------------TAC---AACTTTG-GAGTTAGATAAAA-TATT  2702
1b-FO393392      2672 --------GG--AA--------------TAC---TAATATT-GAATTAACAAAAT-TGCT  2702
2-AAJO01000077   2538 TATTCTACGA--AATAATTTTTTGATACAGCAAGTAACTTTTAAAATAGCACCGTATGTA  2595
3-AL766849       2633 --------GA--AA--------------AGC---TGATTTT-AA--TAGTTCAGT-TGTA  2661
4-AF355776       2910 ---TATGTTACAAAGAATAACCTGATAAGGAAAATTTTTATGATAGTTGCTCCGTACATA  2966
5-AE009948       2946 ATTTATGTGACAAAGAATAGCTTAATAAAAAGAGTATTTATGAAATTAGCACCCTATGTA  3005
6-HF952106       2633 --------GA--AA--------------AGC---TGATTTT---AATAGTTCAGT-TGTA  2661
7-AY376403       2907 ATTTATCTAACAAAGAATAGTCTAATAAAAAGAGTATTTATGAAATTAGCACCATATATA  2966
8-AY375363       2596 -------TGACATA--------------GATAGTTATTTTT-----TAGCAAAAGGTAAA  2629
9-CGBY01000002   2945 ATTTATGTGACAAAGAATAATTTAGTAAAAAGAGTATTTATGAAATTAGCACCATATATA  3004
                                   *                                *

1a-CP000114      2703 AAAAGCATTTTT---AT--TTA-------ATGGGTTAATCCTATTTTTTTTAGG---GGG  2747
1b-FO393392      2703 AAAATCATTTTT---GT--TTA-------ATGCAATTATTTTGTCTTGTTTAGG---ATT  2747
2-AAJO01000077   2596 CAGTTTATTGCG---ATGTTTAGTTTGTTATTATTTTCTTTATTTTTCTTTAAT---ACT  2649
```

```
3-AL766849       2662 AGGAATGTGGTA---AAGGTTA-------ACTATTTTGTGTTGTTTCTTATAAC---AGT  2708
4-AF355776       2967 CAACTGTTCTTGTTAGCATTTACTTT---TCTTTGCTCTACTATTTTTTTCAACTCAAAT  3023
5-AE009948       3006 CAATTTTTTTTATTAGTATTTACCTT---TTTGAGTTCTACAATTTTTTTTAATTCAAAT  3062
6-HF952106       2662 AGGAATGTGGTA---AAGGTTA-------ACTATTTTGTGTTGTTTCTTATAAC---AGT  2708
7-AY376403       2967 CAGTTTTTTTTATTAGCATTTACTTT---TTTGAGTTCTACAATTTTTTTCAATTCAAAT  3023
8-AY375363       2630 CATTAGACTCTAAAAATCAATAATTT---ATTTTTTTCTTCATTGTCTTTTATGGGGATT  2686
9-CGBY01000002   3005 CAACTTTTTTTATTAGTATTTACCTT---TTTGAGTTCTACATTTTTTTTCAATTCAAAT  3061
                                          **                *    * *  *  *

1a-CP000114      2748 AACAT---------ATTA--TTATTGTTTGCATAATAATATTCAAAATATCAGTATTT--  2794
1b-FO393392      2748 TCTAT---------ATTA--TTATGCCATATATTTTGATGTAGAGAATGTAAGTCTTT--  2794
2-AAJO01000077   2650 CCTATTGTTTCAAAATTAGATAACCTTTTAAGTGGTAGAATTTATTACGCGAAGCTTA--  2707
3-AL766849       2709 TTTAT---------ATT----TATTTTTTCC-------TATGCTGAAGCCAACTTTAT--  2746
4-AF355776       3024 TTTGT---TCAAAAATTAGATAGCCTTTTGACAGGTAGGTTAAACTATGCTCATTTACAG  3080
5-AE009948       3063 TTTGT---TCAAAAATTAGATGTTCTTTTAACAGGTAGATTACACTATGCTCATTTACAA  3119
6-HF952106       2709 TTTAT-ATTT----ATT------TTTTCCAAATG----AATTTACTACAT-----TCC--  2746
7-AY376403       3024 TTTGT---TCAAAAATTAGATAAGCTTTTGACAGGTAGGTTAAACTATGCTCATTTACAA  3080
8-AY375363       2687 GTTTT-----AGGAGGTTCCCAAGTTTTCAAT-----AATTTTGCTATTGATAGTT----  2732
9-CGBY01000002   3062 TTTGT---TCAAAAATTAGATGGTCTTTTGACAGGTAGATTAAACTATGCTCATTTACAA  3118
                          *           *                       *     *        *

1a-CP000114      2795 -TTGGTAGAGATTTGATT---GGGTCAG--ACTGGATTAA------------TGGTATGC  2836
1b-FO393392      2795 -TTGGAAGAAATTTAATT---GGATCAG--ATTGGATAAA------------TGGGATGC  2836
2-AAJO01000077   2708 -TCTTAACAGATTCACTTAATTTATTTGGAAATGAAATCAAC--------TATTTTATTG  2758
3-AL766849       2747 -TTGGAAGAGAATTGTTT---TCAATAG--AGTGGTTTCC------------ACATATGA  2788
4-AF355776       3081 CTTGTAGACGGCTTAACT---CTTTTTGGAAATAGTTTTAAGGAGACGAGTGTCCTATTT  3137
5-AE009948       3120 CTTGTAGATGGTTTAACT---CCTTTTGGAAATAGTTTTAAGGAAACAAGTGTCCTATTT  3176
6-HF952106       2747 -TAGGAAGAGATTTATTT---TCAATTG--AATGGATTCC------------TTCTATGA  2788
7-AY376403       3081 CTTGTAGACGGTTTGACT---CTTTTTGGAAATAGTTTTAAGGAAACAAGTGTCCTGTTT  3137
8-AY375363       2733 -CTCTAAAGTATTTATCT----TATTTCATCTTATTTTTA----------TGTATTATTG  2777
9-CGBY01000002   3119 CTTGTAGACGGCTTAACT---CTTTTTGGAAATAGTTTTAAGGAGACAAGTGTCCTATTT  3175
                                  *    *              *                        *

1a-CP000114      2837 ATACTCAAAGAGCAATGGGATTTT-TTGA--ATA----------TTCAAACCTTA-----  2878
1b-FO393392      2837 ATACGCAGAGAGCAATGGCTTTCT-TTGA--ATA----------TTCAAATCTTA-----  2878
2-AAJO01000077   2759 A------TAGATATATTTTATTTT-TTCATGATAACAGTTATTCTTCAACACTTGC----  2807
3-AL766849       2789 GAAT---AAGACTTGCGGCATATT-TTGA--ATA----------TGCTACACTAA-----  2827
4-AF355776       3138 GA----TAATAGCTACTCTATGTTATTGAGTATG-----TATGGTGTAGTACTTACCATG  3188
5-AE009948       3177 GA----TAATAGCTACTCTATGTTATTGAGTATG-----TATGGTGTAGTACTTACCATG  3227
6-HF952106       2789 AAGT---TAGACTTACTGCATATT-TTGA--GTA----------TTCAACACT-------  2825
7-AY376403       3138 GA----TAATAGCTACTCTATGTTATTGAGTATG-----TATGGTGTAGTCCTTACCCTG  3188
8-AY375363       2778 TTA----AACTCTTAAGAAATCCC-TTAACCATA--------------ATATTTA-----  2813
9-CGBY01000002   3176 GA----TAATAGCTACTCTATGTTATTGAGTATG-----TATGGTGTAGTACTTACAATG  3226
                              *           *    ** *   *                   *
```

```
1a-CP000114       2879 -----TAATTC---CTATTACAGTG-----------------------GTAACTAACTTA  2907
1b-FO393392       2879 -----TAATACCCTTAACTATCAT--------------------------AACTAATATA  2907
2-AAJO01000077    2808 ---ACTAAGTGGAATTATTATTAC---------------------------ATTTGGGTA  2837
3-AL766849        2828 -----TTGGTCAGTTTATTTTATT-------------------------TTCTTATCCCA  2857
4-AF355776        3189 TTTTGTATGATAATCTATTATATCTATAGTAAAAAAGTCAATGTAGTTGAGCTCCAGATA  3248
5-AE009948        3228 TTTTGTATGATAATCTATTATATCTATAGTAAAAAGATAATCATAATTGAACTTCAACTA  3287
6-HF952106        2826 ---ATTAGGTCAGTTTATTTT------------------------------ATTCACTTA  2852
7-AY376403        3189 TTTTGTATGATAATCTATTATGTTTACAGTAAAGAAATAAGAATAGTTGAACTTCAATTA  3248
8-AY375363        2814 ----GTAGATTGGTCTATTTTAT---------------------------ATGTATTTCA  2842
9-CGBY01000002    3227 TTTTGTATGATAATCTATTATATCTATAGTAAAAAGATAATCATAATTGAACTTCAACTA  3286
                            *          * *                                        *

1a-CP000114       2908 TATATATATAT-ATATAT-GAA----GTTA---AGAAACT---ATTCAA--TTATGAC--  2951
1b-FO393392       2908 TATATATATAT-ATATAT-TAA----GCAA---AGATATA---GCTCAG--GGATGAT--  2951
2-AAJO01000077    2838 TATGTACTT---ATATTT-TAAAACCTCTC---GAAAGTT---AGTGCA--GGATCAT--  2883
3-AL766849        2858 TACTTTTTTTG-AAACCC-CAAAAACGTAT---GGAAAAT---ATTTTA--ATAT--C--  2903
4-AF355776        3249 CTTTTGTTTATAATGTCTATAGTATTATTTACAGAGAGTTTTTACCCAA--GTATAGTTA  3306
5-AE009948        3288 CTCCTATTTATAATGTCTATAATATTATTTACTGAAAGTTTTTATCCCA--GTGTGGTAA  3345
6-HF952106        2853 TCCGATATT---AT-TTT-TAAAACAGC------AGAGGT---A-TGGA--GAA------  2889
7-AY376403        3249 CTGCTATTTATAATGTCTATAGTATTATTTACAGAGAGTTTTTACCCAA--GTATAGTTA  3306
8-AY375363        2843 TTCCTTTTTATAAT-----TGGAATCTATTTGCAAGATCTACCATTACATAGGATAAT--  2895
9-CGBY01000002    3287 CTCCTATTTATAATGTCTATAATATTATTTACTGAAAGTTTTTATCCCA--GTGTGGTAA  3344
                               *   *

1a-CP000114       2952 --CATAGGTGTTGTATTAT-TA-------------TTTACCTTTATTTTAC---------  2986
1b-FO393392       2952 --GATACTCGGTGCTCT-TCTC-------------TCCACTATTATACTAC---------  2986
2-AAJO01000077    2884 --AATAT-TGCTGCTTTATATTTATTTGTTTCAAATTCCTTGTTATTTTACTCCGAAGAT  2940
3-AL766849        2904 --CTTAC-TGTTG-ACTATATG-------------TTCATACTT-TTCTGG---------  2936
4-AF355776        3307 TGAATATTAGTTGGATGGTTTTTGGGAAAAT--ATTTTGTGGGGGTGTAGATG-------  3357
5-AE009948        3346 TGAATATTAGTTGGCTAGTTTTTGGTAAAAT--ATTTTGTGATGGTATCGAAC-------  3396
6-HF952106        2890 --AATAT-TTTTA--TCACACT------------ATTCCTAGTTTTTT------------  2920
7-AY376403        3307 TGAATATTAGTTGGTTAGTTTTTGGGAAAAT--ATTTTGTGATGGTATTGAAC-------  3357
8-AY375363        2896 ---ATATCTTTCATTTTCTATG-------------ATCATAAT--TTCTGG---------  2928
9-CGBY01000002    3345 TGAATATTAGTTGGCTAGTTTTTGGTAAAAT--ATTTTGTGATGGTATCGAAC-------  3395
                           **                                       *

1a-CP000114       2987 ---------------CTATTGGATCGGGCTCCA-------------GGGCTGGAATAGTA  3018
1b-FO393392       2987 ---------------CCATCGGGTCTGGATCTA-------------GAGCTGGTATTATA  3018
2-AAJO01000077    2941 TATATACGCGAGCCATTTTTAAATATTACTTTATTTTT--------TATAGGTAAATATA  2992
3-AL766849        2937 ---------------CGCTAGAAT---ACTATT-------------GGTCTGTA-TGTTG  2964
4-AF355776        3358 ---------------ATTTACAACGAGAGTTCACTTGGACGGCAAATAAAAATTAGTGTA  3402
5-AE009948        3397 ---------------CTATAAAAAAGGAATTTACTATTGTGAATAATATATGACATATTT  3441
6-HF952106        2921 -------GTG---CATATTTGA------------------------CAGGGGCAAGAATT  2946
```

```
7-AY376403        3358 ---------------CAATAAAAAAAGAATTTACTATTATAGACGATATATGATATATTT  3402
8-AY375363        2929 ----------------TTTGGCA----ACTTTA----------------ACGGTATCTTT  2952
9-CGBY01000002    3396 ---------------CTATAAAAAAGGAATTTACTATTGTGAATGATATATGACATATTT  3440
                                         *                                       *

1a-CP000114       3019 GCTATATTGGCGCAGAT-------------GTTTATTCTTCTTCTAAATACA-G--TT--  3060
1b-FO393392       3019 GTTGTGCTACTACAGGTT-------------ATAATTTTATTGTTGAATACA-A--TT--  3060
2-AAJO01000077    2993 TATTTAATGAATTAGGAGAGATAAATGAGTGATAAATTTTCAAATAAAATCA-GTGTTAT  3051
3-AL766849        2965 GTTTTATTAGCTTCGCTTC------TTTTAGATTATATCCTTTTTAAAACTA-A--TTTG  3015
4-AF355776        3403 ATTGTACCAGTATATAATT--------CGAAACAATATT-----TAATAGCTTGCGTTG-  3448
5-AE009948        3442 GCTCTGATATGGCAGGAGGTA-----AGGAAGGAAAATGATACCTAAAGTTATACATTAT  3496
6-HF952106        2947 TTTCTAATT----------------TGTATGATAATTTT---------ATTA-G-GTTAT  2979
7-AY376403        3403 ATTCTGATATAGTAGGATGTG-----AGGAAACAAA-TGATACCTAAAGTTATACATTAT  3456
8-AY375363        2953 ATT-TGATAATGTAAAAGATT-----TAAGAAGAGTATCTT-------ATTATATATT--  2997
9-CGBY01000002    3441 GCTCTGATATGGCAGGAGGTA-----AGGAAGGAAAATGATACCTAAAGTTATACATTAT  3495
                         * *                                                   **

1a-CP000114       3061 --GTCG-TAA----------AGAAGAAAAC-TATAAA-----------------------  3083
1b-FO393392       3061 ---GTAATAA----------AAAGACAAAC-GATAAG-----------------------  3083
2-AAJO01000077    3052 AGTTCCTTTATATAATGTAGAGAAATATAT-TATAAATTGTATTTCTTCACTTACGAATC  3110
3-AL766849        3016 AAATTGACCA----------AGAAAAACAC-TTTTAT-----------------------  3041
4-AF355776        3449 -ATTCAATTA----------GAAAACAAACATATAAG-----------------------  3474
5-AE009948        3497 TGTTGGTTTGGA--------GGAAATCCCT-TACCAGAT---------------------  3526
6-HF952106        2980 --TTACTCTT----------AGAAATA-AT-CATTAAT----------------------  3003
7-AY376403        3457 TGTTGGTTTGGA--------GGAAATCCCT-TACCTGAT---------------------  3486
8-AY375363        2998 -------TTAT---------GGAGTCTTAT-TATCAACT---------------------  3019
9-CGBY01000002    3496 TGTTGGTTTGGA--------GGAAATCCCT-TACCAGAT---------------------  3525
                                             *

1a-CP000114       3084 --ATTTTTA---TTGTACATACTTCCGT------------TTCTACTA-GTAATAGTAAT  3125
1b-FO393392       3084 --ATTTTTC---CTGTATTTAGTTCCGA------------TACTAATA-TTACTATTAGT  3125
2-AAJO01000077    3111 AAACTTATCAGAATATAGAAATTATTTTGATAGATGATGGTTCTACTG-ATAATAGTGGT  3169
3-AL766849        3042 --ACTTG-------GTATG-ACTTTCTT------------ATTTATCA-CCGCTTGT---  3075
4-AF355776        3475 -AATTTGGAA-ATTATTCTTGTTAATGATGGATCAACAGATGGTAGTAAAGAGTTATGTG  3532
5-AE009948        3527 -AATTTAAAGAAATATATAAAAACTTGGAGAGAACAATGTCCGGATTATGAAATTATTGA  3585
6-HF952106        3004 AAATTTA-----ACATAAAAATTACTAAAAAAGCTG----TCTTTTTG-ATAATTATAG-  3052
7-AY376403        3487 -AATTTAAAAAAGTACATAAAAACCTGGAGAGAACAATGTCCAGATTACGAAATTATTGA  3545
8-AY375363        3020 ATACTTGGAATTTTCTATCAAGTTCCCATG-----------TTTACTG-----TTGCAG-  3062
9-CGBY01000002    3526 -AATTTAAAGAAATATATAAAAACTTGGAGAGAACAATGTCCGGATTATGAAATTATTGA  3584
                         * **                                               *

1a-CP000114       3126 -----------GATGTTA-----------------------TAT----TTTGATAAC---  3144
1b-FO393392       3126 -----------GATATTA-----------------------CGT----TTTGATAAT---  3144
2-AAJO01000077    3170 AGGATATGCAAAAAATTAGCTCAAGAGGATCATCGTATTATTTA----TTTAAGGAAAGA  3225
```

```
3-AL766849       3076 -----------------------------------------TTT----TCTTATAAC---  3087
4-AF355776       3533 AGGAGATAAG-AAAATCAG------------ATGAAAGAATTAAGACATTTCACAAAACA  3579
5-AE009948       3586 ATGGAATGAGCATAATTAT--------GATGTTAGTAAAAATGT----TTTTATGAGA--  3631
6-HF952106       3053 -GGATA------ATATTA-----------TTATTGGTATGTTTT----TCTTACAAA---  3087
7-AY376403       3546 ATGGAACGAACGTAATTAT--------GATGTTACCAAAAATAT----TTTTATGAGA--  3591
8-AY375363       3063 -------------------------------------------------TTGAGGGGG--  3071
9-CGBY01000002   3585 ATGGAATGAGCATAATTAT--------GATGTTAGTAAAAATGT----TTTTATGAGA--  3630
                                                                          * *

1a-CP000114      3145 --------------TTACTA-TCTATATATTATCGTATAATTAAT----TTGCG-ATCCG  3184
1b-FO393392      3145 --------------TTGGTG-AGCATATATAATAGAATAATCAAT----TTGCG-GTCGG  3184
2-AAJO01000077   3226 AAATGGTGGGGTTTCTAGTGCTCGAAATTTAGGGCTACAATATGC----AACAGGAAGTT  3281
3-AL766849       3088 --------------ATATGG-TCAATAATTGAAAAAATAATTATG----TACAG-AAACC  3127
4-AF355776       3580 AA-TGGAGGACAATCAAGCGCAAGGAATTTAGGTATTTTATACTC----TACAGGAGATT  3634
5-AE009948       3632 -------GAAGCATATACTA-AGAAGAATTTTGCTTATGTTTCTGACTATGCAAGATTGG  3683
6-HF952106       3088 -----GTGGAGT--CTATTA-TCAA---TTA----TATAATACAC----TATAG-ATTTC  3127
7-AY376403       3592 -------GAAGCATATACTA-AGAAAAATTTTGCCTATGTTTCTGATTATGCAAGGTTGG  3643
8-AY375363       3072 --------------TTGGTG-CCACTTCTTTAGGTTTTAATGGGG--------GAATGTT  3108
9-CGBY01000002   3631 -------GAAGCATATACTA-AGAAGAATTTTGCTTATGTTTCTGACTATGCAAGATTGG  3682
                                                   *          *

1a-CP000114      3185 GGAGTAGTGAATCC------AGAT----TTTCTTTATATAAA-----------GATAC--  3221
1b-FO393392      3185 GAAGTAGTGAATCT------AGAT----TTTCTTTGTACAAG-----------GATAC--  3221
2-AAJO01000077   3282 ACATTGGTTTTGTA------GACTCAGATGACTATATATCAAAAACAATGTATGAAAACT  3335
3-AL766849       3128 AAAGTACTATCACT------AGGA----TGATAGTTTATCAA-----------GAAAG--  3164
4-AF355776       3635 TGATTGGTTTTGTTGACAG-CGACGATACAATTGACCCTAAAA-----TGTATGAAACGT  3688
5-AE009948       3684 ATATTATTTATACTTATGGGGGGTTC--TATCTAGATACTGA--------TGTGGAGC--  3731
6-HF952106       3128 AAAGTAGTAGTACA------AGAT----TGACAGTCTAT-----------TACGAAAG--  3164
7-AY376403       3644 ATATTATTTACACTTATGGCGGATTC--TATCTAGACACTGA--------TGTGGAAC--  3691
8-AY375363       3109 ACACAAAAATTTCT--------ATGCGATAACTATTCT-----------------AACTT  3143
9-CGBY01000002   3683 ATATTATTTATACTTATGGGGGGTTC--TATCTAGATACTGA--------TGTGGAGC--  3730
                        *

1a-CP000114      3222 AGTAAACATCGTTA-----TAAAT---------AATTCTT----------------TATT  3251
1b-FO393392      3222 CGTACACTCAGTAA-----TTACT---------GACTCAC----------------TATT  3251
2-AAJO01000077   3336 TATTAAAGAGATTA-----TTAGAAACTAATGCGGATATCGCCGAAACTGACTTTGCCTT  3390
3-AL766849       3165 TATTATTGAAGTTC-----TAAAA---------GGAAATA----------------TTTT  3194
4-AF355776       3689 TACTAAATATATATGAAGATGAACAAGTAGACTGGGTGCA---------------ATGTA  3733
5-AE009948       3732 TTTTAAAAAGTTTAGATCCTTTGA---------GGATTCA------------TGAGTGTT  3770
6-HF952106       3165 TATAAGAGCGATT------TTAGA------TGGGAATTTC-----------------CTT  3195
7-AY376403       3692 TTTTAAAAAGTTTAGACCCTTTGA---------GAGTTCA------------TAACTGTT  3730
8-AY375363       3144 CATATATACTATTA----------------------TTTA------------------TA  3163
9-CGBY01000002   3731 TTTTAAAAAGTTTAGATCCTTTGA---------GGATTCA------------TGAGTGTT  3769
                                 *                                              *
```

```
1a-CP000114       3252 ATTTGGAGA--------------------------AGGAGTTAAAGAGTTATGGT-TAAA  3284
1b-FO393392       3252 TCTGGGAAA--------------------------AGGTGTAAAAGAATTGTGGT-TAAA  3284
2-AAJO01000077    3391 AATTGATAATAGATTTACGAAAAAAAAGAGGAAAAAGATAC-AGAAAGTTCTGAA-TAAA  3448
3-AL766849        3195 ATTTGGACA--------------------------GGGTAT--AAGGATTCCA-T-CAAG  3224
4-AF355776        3734 ATCACAAAA--------------------------AAATTT------ACTCTAAC---GG  3758
5-AE009948        3771 TTCTAGCAA-------------------------GGGAGAT----TAGTTGTGATGTGAA  3801
6-HF952106        3196 ATTGGGCAA---------------------------GGTAT-A-AGAGTTCCCTC-CAGT  3225
7-AY376403        3731 TTTTAGCTA-------------------------GGGAGTT----AAGTTATGATGTGAA  3761
8-AY375363        3164 AGTAGGAAAT------------------------ATGGAGT----AAGGCATAATATAGA  3195
9-CGBY01000002    3770 TTCTAGCAA-------------------------GGGAGAT----TAGTTGTGATGTTGC  3800
                               *

1a-CP000114       3285 TAGTGATCT--------ACCTTTG-----------GGGTCGCA-----TTCAACGTATA-  3319
1b-FO393392       3285 TAGTGATTT--------ACCATTA-----------GGATCGCA-----TTCGACCTACA-  3319
2-AAJO01000077    3449 GAAGAAGCTATTAGGGAGTTTTTATCAGGAAATGTGGTTGAAAATAATCTCGTCATAAAG  3508
3-AL766849        3225 TGAAGGAAT--------ATTCCTA-----------GGATCGCA-----TTCTACGTATA-  3259
4-AF355776        3759 TGTTAACTTAT------ATTATAAT--GGACCTGAATACTATAATGTGCTTAATAAACAA  3810
5-AE009948        3802 TACAGGATT-------AATAATTGGCGCTGTTAAAGGACATCACT--TTTTAAAATCAAA  3852
6-HF952106        3226 GTGGGAAT---------ATTTTTA-----------GGTTCACA-----TTCATCATACA-  3259
7-AY376403        3762 TACAGGACT-------AATTATTGGTGCAATTAGAGGACAAAAAT--TTATAAAAGACAA  3812
8-AY375363        3196 TAGTTATGT--------ACTTT-------------GGTTTGAA----TTCTTCCTTATA-  3229
9-CGBY01000002    3801 CACTGGATT-------AATTATTGGTGCAGTTAGAGGACAAAAAT--TTATAAAAGATAA  3851
                                                                 *               *

1a-CP000114       3320 ----TAGG--------------------CTATTTCT---------ACAAAA---------  3337
1b-FO393392       3320 ----TAGG--------------------TTATTTCT---------ATAAAA---------  3337
2-AAJO01000077    3509 TTATTTAAAAAAACAGTTATTGCTAATCTAAAATTT---------AAAGAAGATGTGATA  3559
3-AL766849        3260 ----TTAG--------------------TGTCTTTT---------ACAGGA---------  3277
4-AF355776        3811 GATTTCCTA-----------------TACGAATTTCTGAGTACAAATAAGA---------  3844
5-AE009948        3853 TATGTCTATATATGACAAAAGTGA---TTTAACTTCTC----TTAATAAGA---------  3896
6-HF952106        3260 ----TTAG--------------------TATATTTT---------ATAGAA---------  3277
7-AY376403        3813 TATGTCGGTATACGAAGAATCTAAATTTTTAAGTT-------TTGATAAAA---------  3856
8-AY375363        3230 ----TTGA------------------TTTCAAATTCAC-------GTACAG---------  3251
9-CGBY01000002    3852 TATGATGGTATATGAAAAATATAAATTTTTAAATT-------TTGATAAAA---------  3895
                                                        *             *

1a-CP000114       3338 ------------------GTGGCCTGCTGGGATTAATGAA--------------------  3359
1b-FO393392       3338 ------------------CTGGCCTATTTGGACTAATAAA--------------------  3359
2-AAJO01000077    3560 GTTGGTGAAGATATGCTATTTTCTTTGCAGGCCTTACAAAACTCAGATAGAGTTACAGTG  3619
3-AL766849        3278 ------------------CTTCTTTATTAGGA----------------------------  3291
4-AF355776        3845 --------------------TTTTTAGTTCAGTCTGCGAG--------------------  3864
5-AE009948        3897 ---------------------CATGTGTAGAGGTTACAAC--------------------  3915
6-HF952106        3278 ------------------CTTCTTTTACGGGGCT--------------------------  3293
```

```
7-AY376403       3857 ---------------------CATGTGTAGAAATCACGAC--------------------  3875
8-AY375363       3252 ---------------------TGTATTTAATTCTAATCAT--------------------  3270
9-CGBY01000002   3896 ---------------------CTTGTGTGGAAATTACGAC--------------------  3914


1a-CP000114      3360 ---------------TATAGTT--CCAGGTTTGC---TTTTAATTTT--------TACTA  3391
1b-FO393392      3360 ---------------TGTGATT--TTAGGTTTGT---TTCTAATTTT--------TATTA  3391
2-AAJO01000077   3620 GACACTACGAATGCGGATTATT--TTTATGTCGTACGTTCAAATTCAACTATGAATACTA  3677
3-AL766849       3292 ----------------ATTGTT--CTTTATTT-----TTCTGCCTTTA-------TACTT  3321
4-AF355776       3865 --------------GGGTTGTTATCTAGAGATTTAGCTTTAAAAATAA--------AATT  3902
5-AE009948       3916 -------------TAATTTATT--GATAAACAGAGGGCTTAAGAATAAG-------AATA  3953
6-HF952106       3294 ---------------GTTTCTT--TTCTT--------TTCAA----------------TA  3312
7-AY376403       3876 -------------CAGCTTACT--TTTAAAAAAAGGACTGAAAAGTAAA-------AATA  3913
8-AY375363       3271 --------------ATTTTATTG-TATAAATAA----TTGGAA-------------GTTA  3298
9-CGBY01000002   3915 -------------TGACTTACT--TTTAAAAAAAGGATTGAAGAATAAA-------GATG  3952
                                       *   *                *                   *

1a-CP000114      3392 ATATTGGTAGGAAAG------------CTAAAC--------------AATCAGCTTT---  3422
1b-FO393392      3392 GCATTATCAAGGAAG------------CTAAAA--------------AGTCAGATTT---  3422
2-AAJO01000077   3678 TTACCGAGAAGGATATCGATAATCTTTCAATACTCGAGCAGGAGTTCAAAAAAATTGATA  3737
3-AL766849       3322 TTAT--ATAAAGAAG------------CGATAT--------------CAAAAA-------  3346
4-AF355776       3903 CCGTGAAGAAAAAAA--------------ATAT--------------GAAGATACACAG-  3933
5-AE009948       3954 TTATTCAAAAGATTG---ATGATATAACAATAT-----------ATCCGAGAAATTA---  3996
6-HF952106       3313 TTAC------------------TTTTTCTATAT--------------AGAGAAGCTA---  3337
7-AY376403       3914 TCATACAAGTTATTG---ATGGTGTCACTATTT-----------ATCCAAGACAATA---  3956
8-AY375363       3299 TTATTAAGAA------------------TAAAT---------------AAAAAG------  3319
9-CGBY01000002   3953 TCATACAAGTTATAG---ATGGTGTTACTATTT-----------ATCCAAGACAATA---  3995
                                                   *                     *

1a-CP000114      3423 -----TTATTATGAGA-T--------AGTAGGAACA------------------CTTATA  3450
1b-FO393392      3423 -----CTATTATGAGA-T--------AGTAGGGTCT------------------GTCATA  3450
2-AAJO01000077   3738 TCCCCCAATTAAAAAG-TTATTTGGAAGCCAAATTAATTCGAGAAAAGGTTAAATTTGTA  3796
3-AL766849       3347 -------ATTATAAAA-T--------CTACAGATTA------------------TTTTTT  3372
4-AF355776       3934 ---TTTTATTTTGATCTC--------ATAAAAAATG------------------CTAATA  3964
5-AE009948       3997 --TTTTAATCCAAAGAATTTATTAACAGGTAAGGTT-----------------GATTGTC  4037
6-HF952106       3338 ----TCAAACAAAA---------------CAGGATAATCTA---------CAAGCTTTTT  3369
7-AY376403       3957 --CTTTAATCCTAAAAATTTGTTGACAGGAAAGCTA-----------------GATTTTC  3997
8-AY375363       3320 ---------CAAAGAATT---------------TTA-----------------GTTTTTA  3338
9-CGBY01000002   3996 --TTTTAATCCTAAAAGTATGTTGACGGGGAGGTTA-----------------GATTTTC  4036
                                                                         *  *

1a-CP000114      3451 ACTTTATTCTCA------------------TTTTTTG----CACT---------------  3473
1b-FO393392      3451 CTCCTATTTTCA------------------TTGTTTG----CACT---------------  3473
2-AAJO01000077   3797 TCTAGAGTATTAATTAGTAATAAAGCACATTTGTACAGTGATATTATTGATAGATATTTG  3856
```

```
3-AL766849        3373 TATACGTTATTA------------------TGTTACA----CGCTCTT------------  3398
4-AF355776        3965 AGTTTGTTATTATAAGCCAA-------CCTTTTTATA--ATTACTACTAC----------  4005
5-AE009948        4038 TGACTAGTGTTACCTA--------------TTCTATA-CATCATTACGAA----------  4072
6-HF952106        3370 TTTGGATTGTTA-TTA--------------TTGTATA-TGGTATT---------------  3398
7-AY376403        3998 TAACAAATGATACTTA--------------TTCTATA-CATCATTATGAA----------  4032
8-AY375363        3339 TCCCCGCTA-TATTTA-------------TTTCTTTA-----ATTA--------------  3365
9-CGBY01000002    4037 TAACAGATGATACATA--------------CTCTATA-CACCATTATGAA----------  4071
                              *   *                     *          *

1a-CP000114       3474 ----------------------TGAAGATCTTGAC-----GGAGCTAATTGGCTTATTGT  3506
1b-FO393392       3474 ----------------------TGAAGATATTGAT-----GGCGCCAATTGGCTCATTAT  3506
2-AAJO01000077    3857 CAAGAGGTTAGACACTACAGCATAAAAAATATGATAAACTTTCTTTCGTCGAAACATTGT  3916
3-AL766849        3399 ----------------------TGAGGAAATAGAT-----CCTAATCATTGGAGTATTGT  3431
4-AF355776        4006 ---------------------AGAAAAAATAGTACAACAACTTCCTCATATAGTAGCTAT  4044
5-AE009948        4073 ----------------GGAAGTTGGAAAAGTTCTTCATTTATTTCAGATTCTCTAAAGAT  4116
6-HF952106        3399 ----------------------TGAAGAATTTGAT-----CCTAATCATTGGAGTGTTGT  3431
7-AY376403        4033 ----------------GGAAGTTGGAAAAAGCCATCTATTTTTCCTGATTCGATGAAAAT  4076
8-AY375363        3366 ----------------------TAGCGATAATATT----TTTTGAATACTTAACCAATAA  3399
9-CGBY01000002    4072 ----------------GGAAGCTGGAAAAAAAGATCATTTGTTCCTGATTCCCTAAAGAT  4115
                                                  *

1a-CP000114       3507 TT---T--TATTTTTACAGTGTTA-----GGAATTTTAGAAAATAA-GGATTTTTATAGT  3555
1b-FO393392       3507 TT---T--TGTCTTTACAATGTTG-----GGAGTTTTAGAAAATAA-GGATTTCTATAGT  3555
2-AAJO01000077    3917 TTGACTATAACTATTATGAAAATA-----TCACCTAAACTATATAC-ACTTTTATATAAA  3970
3-AL766849        3432 AT---TATTATTCTCA---------------ACTTTTGG---------------TATAGT  3458
4-AF355776        4045 CAATGGGACATAATCGA-------------TATCTGTACTGAGTGTTATTATTATGCAAA  4091
5-AE009948        4117 TAGAGTAAGGCTCATAATTGATTTTTTATTTGGATATGGTACTTAT-AGAATGCTTCTAA  4175
6-HF952106        3432 ---------ATTGTTAT------------TTAC-TACATTAGGTAT-A-------GTAGG  3461
7-AY376403        4077 TAGAGTAAGACTTGTAATTGATTTTATATTTGGATATGGAACATAT-AGAAGGCTGTTGC  4135
8-AY375363        3400 TAG----CGACTCCTAT--------------ACTTATAGAGTTCAAGGGATA---ATTAA  3438
9-CGBY01000002    4116 TAGATTACGTCTTATAATCGATTTTGTGTTCGGATATGGAACATAT-AGAAGGATATTGC  4174
                                                         *

1a-CP000114       3556 CAACTTAAAAGG---TGGAAAAG-------------------------------------  3575
1b-FO393392       3556 CAACTTAAAAGG---TGGGAAAG-------------------------------------  3575
2-AAJO01000077    3971 GTTTTTCAAAAG---CAATAAATATCGGAACGGAG-------------------------  4002
3-AL766849        3459 GGG----AAGGG---CTAAAAAA-------------------------------------  3474
4-AF355776        4092 GGATTTTAATGGATTTGAAGAAGTTGC---------------------------------  4118
5-AE009948        4176 GGTTTCTAAAGT---TAAAGAAATAGTTATTATTGTAATAGAAAGGACGAAATGTTATAT  4232
6-HF952106        3462 G------AGAGG---GAATGA---------------------------------------  3473
7-AY376403        4136 GTTTTTTAAAGT---TAAAACAGTAGTCGCTATT--------------------------  4166
8-AY375363        3439 TTTTTTTAA------TTATTA---------------------------------------  3453
9-CGBY01000002    4175 GTTTTTTAAAAT---TAAAATAGTAGCAGTTATT--------------------------  4205
                              *
```

```
1a-CP000114      3576 ------------------------------------------------------------  3575
1b-FO393392      3576 ------------------------------------------------------------  3575
2-AAJO01000077   4003 ------------------------------------------------------------  4002
3-AL766849       3475 ------------------------------------------------------------  3474
4-AF355776       4119 ------------------------------------------------------------  4118
5-AE009948       4233 ATTTTTACTCCTACATTTAACAGAGGCTACCGATTATCCTATCTGTATGATTCTCTATGT  4292
6-HF952106       3474 ------------------------------------------------------------  3473
7-AY376403       4167 ------------------------------------------------------------  4166
8-AY375363       3454 ------------------------------------------------------------  3453
9-CGBY01000002   4206 ------------------------------------------------------------  4205

1a-CP000114      3576 ------------------------------------------------------------  3575
1b-FO393392      3576 ------------------------------------------------------------  3575
2-AAJO01000077   4003 ------------------------------------------------------------  4002
3-AL766849       3475 ------------------------------------------------------------  3474
4-AF355776       4119 ------------------------------------------------------------  4118
5-AE009948       4293 AATCAGACAAATAAAAATTTTATATGGTTGATTGTTGATGATGGTTCTGAGGACAGTACT  4352
6-HF952106       3474 ------------------------------------------------------------  3473
7-AY376403       4167 ---------------AACT-----------------------------------------  4170
8-AY375363       3454 ------------------------------------------------------------  3453
9-CGBY01000002   4206 ---------------AATTT----------------------------------------  4210

1a-CP000114      3576 ------------------------------------------------------------  3575
1b-FO393392      3576 ------------------------------------------------------------  3575
2-AAJO01000077   4003 ------------------------------------------------------------  4002
3-AL766849       3475 ------------------------------------------------------------  3474
4-AF355776       4119 ------------------------------------------------------------  4118
5-AE009948       4353 AAGGAAATTGTAAGTAATTATATAAAAGAGAATAAAGTTAGTATTGTATATCTGTATAAA  4412
6-HF952106       3474 ------------------------------------------------------------  3473
7-AY376403       4171 ---GAAAT----------------------------------------------------  4175
8-AY375363       3454 ------------------------------------------------------------  3453
9-CGBY01000002   4211 ---AAAAT----------------------------------------------------  4215

1a-CP000114      3576 ------------------------------------------------------------  3575
1b-FO393392      3576 ------------------------------------------------------------  3575
2-AAJO01000077   4003 ------------------------------------------------------------  4002
3-AL766849       3475 ------------------------------------------------------------  3474
4-AF355776       4119 ------------------------------------------------------------  4118
5-AE009948       4413 CGTAATGGCGGTAAGCATTCAGCCTATAATCTAGCAATGAGATATATGCAACCAAGTGAT  4472
6-HF952106       3474 ------------------------------------------------------------  3473
```

```
7-AY376403       4176 ------------------------------------------------------------  4175
8-AY375363       3454 ------------------------------------------------------------  3453
9-CGBY01000002   4216 ------------------------------------------------------------  4215

1a-CP000114      3576 ----------------------------------------------------------TT  3577
1b-FO393392      3576 ----------------------------------------------------------TT  3577
2-AAJO01000077   4003 ------------------------------------------------------GCTTTT  4008
3-AL766849       3475 ----------------------------------------------------------TG  3476
4-AF355776       4119 -------------------------------------------------------TTTTT  4123
5-AE009948       4473 TATCATGTATGTGTAGATAGTGATGATTGGTTATTAGAAGATGCAGTTGAAATTATTTTT  4532
6-HF952106       3474 -----------------------------------------------------------T  3474
7-AY376403       4176 ---------------------------------------------------ATTTTCTTT  4184
8-AY375363       3454 ----------------------------------------------------------TC  3455
9-CGBY01000002   4216 ---------------------------------------------------ATTCTCTTT  4224

1a-CP000114      3578 AATG--------------------------------------------------------  3581
1b-FO393392      3578 AATG--------------------------------------------------------  3581
2-AAJO01000077   4009 ATTG--------------------------------------------------------  4012
3-AL766849       3477 AAAG--------------------------------------------------------  3480
4-AF355776       4124 CAAG--------------------------------------------------------  4127
5-AE009948       4533 AAAGATTTAGAGAGCCTAACGTTGTCTAATAGATATGTAGGCCTTGTTTATCCTCGATAT  4592
6-HF952106       3475 AAAA--------------------------------------------------------  3478
7-AY376403       4185 AAAG--------------------------------------------------------  4188
8-AY375363       3456 AATC--------------------------------------------------------  3459
9-CGBY01000002   4225 AAA---------------------------------------------------------  4227

1a-CP000114      3582 ----------------------------------GAAA----------------------  3585
1b-FO393392      3582 ----------------------------------GAAA----------------------  3585
2-AAJO01000077   4013 ----------------------------------GAAA----------------------  4016
3-AL766849       3481 ----------------------------------AAAA----------------------  3484
4-AF355776       4128 ------------------------------------------------------------  4127
5-AE009948       4593 TCATTAAACCAAGGTAATAATTGGTTGAATCCCAAAATATTAGAAGTTAATATTCCTGAT  4652
6-HF952106       3479 ----------------------------------AAAC----------------------  3482
7-AY376403       4189 ----------------------------------AAAT----------------------  4192
8-AY375363       3460 ------------------------------------------------------------  3459
9-CGBY01000002   4228 ----------------------------------AAAT----------------------  4231

1a-CP000114      3586 ------------------------------------------------------------  3585
1b-FO393392      3586 ------------------------------------------------------------  3585
2-AAJO01000077   4017 ------------------------------------------------------------  4016
```

```
3-AL766849       3485 ------------------------------------------------------------  3484
4-AF355776       4128 ------------------------------------------------------------  4127
5-AE009948       4653 TTAAAATATAAATATCACTTAAAAATTGAAACTTGTATTGTTATTAATAATGCTTATTTA  4712
6-HF952106       3483 ------------------------------------------------------------  3482
7-AY376403       4193 ------------------------------------------------------------  4192
8-AY375363       3460 ------------------------------------------------------------  3459
9-CGBY01000002   4232 ------------------------------------------------------------  4231

1a-CP000114      3586 ------------------------------------------------AA--CGAATA--  3593
1b-FO393392      3586 ------------------------------------------------AA--CAAATA--  3593
2-AAJO01000077   4017 ------------------------------------------------AGATTAG-----  4023
3-AL766849       3485 ------------------------------------------------AG--TAA-----  3489
4-AF355776       4128 ------------------------------------------------------------  4127
5-AE009948       4713 GTAGATTTCGAATTTCCTTGTTTCGAAGGGGAGAATTTCTTATCAGAAGAAATAATGTAT  4772
6-HF952106       3483 ------------------------------------------------TAGTTAG-----  3489
7-AY376403       4193 ---------------------------------------------GGAGGAACAATG---  4204
8-AY375363       3460 ------------------------------------------------GGACTA------  3465
9-CGBY01000002   4232 ---------------------------------------------GGAGGCACAATG---  4243

1a-CP000114      3594 ------------------------------------------------------------  3593
1b-FO393392      3594 ------------------------------------------------------------  3593
2-AAJO01000077   4024 ------------------------------------------------------------  4023
3-AL766849       3490 ------------------------------------------------------------  3489
4-AF355776       4128 ------------------------------------------------------------  4127
5-AE009948       4773 ATATATTTATCAAAAAAGGGATACTTTTGTCCCCAAAATAGGAAAATCTATTGTTTTGAC  4832
6-HF952106       3490 ------------------------------------------------------------  3489
7-AY376403       4205 ----------------------------------AAAGTA--------------------  4210
8-AY375363       3466 ------------------------------------------------------------  3465
9-CGBY01000002   4244 ----------------------------------AAAGTA--------------------  4249

1a-CP000114      3594 ------------------------------------------------------------  3593
1b-FO393392      3594 ------------------------------------------------------------  3593
2-AAJO01000077   4024 ------------------------------------------------------------  4023
3-AL766849       3490 ------------------------------------------------------------  3489
4-AF355776       4128 ------------------------------------------------------------  4127
5-AE009948       4833 TACTTAGAAGATGGTTTGACTTCTAATATTTTTAAATTATGGAGAAAAAATTTCAAAGGG  4892
6-HF952106       3490 ------------------------------------------------------------  3489
7-AY376403       4211 ------------------------------------------------------------  4210
8-AY375363       3466 ------------------------------------------------------------  3465
9-CGBY01000002   4250 ------------------------------------------------------------  4249
```

```
1a-CP000114      3594 --CTTGTTTCTATCA------TTATACCTATATACAACTC--------------------  3625
1b-FO393392      3594 --CTTGTTTCTATCG------TTATACCTATATACAACTC--------------------  3625
2-AAJO01000077   4024 ----------TATTA------TTATACCGGTATACAATGT--------------------  4047
3-AL766849       3490 ---------CAGTCA------TTATACCTATATACAACTC--------------------  3514
4-AF355776       4128 -----------ATTATTTGGTGCATATTCGTTAGTAGCTA--------------------  4156
5-AE009948       4893 ACTATTTTTTCATTAGAAAATTCATATATGTATGTAATGTCTTTTCCTAATATATTTGAT  4952
6-HF952106       3490 ----------TGTGA------TTGTTCCAGTTTATAATTC--------------------  3513
7-AY376403       4211 ---------TCATTAG-----TCATACCAGTTTATAATG---------------------  4235
8-AY375363       3466 ---------TTTTCACTTGCTTTTTGGTGATGCACAGCTTGCTT----------------  3500
9-CGBY01000002   4250 ---------TCATTAG-----TTATACCAGTTTATAATG---------------------  4274
                                  *           *     *    *

1a-CP000114      3626 ------------------------------------------------------------  3625
1b-FO393392      3626 ------------------------------------------------------------  3625
2-AAJO01000077   4048 ------------------------------------------------------------  4047
3-AL766849       3515 ------------------------------------------------------------  3514
4-AF355776       4157 ------------------------------------------------------------  4156
5-AE009948       4953 CGCTGGTGGTCAGCTATAAAGATAAAAATGAACATACAGGCTTTGAAAATGACTACTTTA  5012
6-HF952106       3514 ------------------------------------------------------------  3513
7-AY376403       4236 ------------------------------------------------------------  4235
8-AY375363       3501 ------------------------------------------------------------  3500
9-CGBY01000002   4275 ------------------------------------------------------------  4274

1a-CP000114      3626 ------------------------------------------------------------  3625
1b-FO393392      3626 ------------------------------------------------------------  3625
2-AAJO01000077   4048 ------------------------------------------------------------  4047
3-AL766849       3515 ------------------------------------------------------------  3514
4-AF355776       4157 ------------------------------------------------------------  4156
5-AE009948       5013 GGGGTAATACCTACACTAAAGAGTGAAGAGGCTGGGTGGAAAATTTTGTTGGGTTTTAGC  5072
6-HF952106       3514 ------------------------------------------------------------  3513
7-AY376403       4236 ------------------------------------------------------------  4235
8-AY375363       3501 ------------------------------------------------------------  3500
9-CGBY01000002   4275 ------------------------------------------------------------  4274

1a-CP000114      3626 ---------------------------AGAAGCA--------------------------  3632
1b-FO393392      3626 ---------------------------GGAAGCA--------------------------  3632
2-AAJO01000077   4048 ---------------------------TAAGA-AG-------------------------  4054
3-AL766849       3515 ---------------------------AGAAGCA--------------------------  3521
4-AF355776       4157 -------------------------ATAAAATTGTA------------------------  4167
5-AE009948       5073 TATCTTTGGAAGGTAGCAAGGTTTAAAAAGAGCGAGTAAAGAATGGAATTAATTTCAATA  5132
6-HF952106       3514 ---------------------------GGAGTTAG-------------------------  3521
```

```
7-AY376403        4236 -----------------------------GGGCACCT-----------------------  4243
8-AY375363        3501 --------------------------TTGGAGGA--------------------------  3508
9-CGBY01000002    4275 -----------------------------GAGCAAAT-----------------------  4282

1a-CP000114       3633 ------------TACCTTAAAGAA-TGTG--------------TGCAATCCG------TA  3659
1b-FO393392       3633 ------------TATCTTAAAGAA-TGCG--------------TGCAATCCG------TC  3659
2-AAJO01000077    4055 ------------TATCTTAATGAT-TGTA--------------TTCAATCTG------TT  4081
3-AL766849        3522 ------------TACCTTAAAGAA-TGTG--------------TGCAATCCG------TA  3548
4-AF355776        4168 ------------TATAATAAAGAT-TATA--------------GAAAAACCGAAGAATTA  4200
5-AE009948        5133 ATAGTGCCTGTATATAATGGAGAAATATATATTGGGAGATGTCTAGATAGTA------TT  5186
6-HF952106        3522 ------------TGA-TTGAGAAC-TGTG--------------TAGAATCTT------TG  3547
7-AY376403        4244 ------------TACATCGAAGAAACAT---------------TAGAAAGTG------TC  4270
8-AY375363        3509 -------------ACAACACAGAATTAT-------------------ACCTA------TA  3530
9-CGBY01000002    4283 ------------CATATCAAAGAAACAT---------------TAGAAAGTA------TT  4309
                                             *                                   *

1a-CP000114       3660 CTACAACAGACTCATCCATTGATAGAAGTTAT-ACTAA------TTGATGATGGATCCAC  3712
1b-FO393392       3660 CTACAACAGACTCATTCATTGATAGAAGTTAT-ACTGA------TTAATGATGGATCCAC  3712
2-AAJO01000077    4082 ATAAACCAGACTTACAATAATTTAGAGATTAT-TTTAA------TTGATGATGGTTCTAC  4134
3-AL766849        3549 CTACAACAGACTCATCCATTGATAGAAGTTAT-ACTAA------TTGATGATGGATCCAC  3601
4-AF355776        4201 AGATAATAG-CTTATTTTTTCAAGAAAAATTT-ATTGAAAATTTTTAAAAATAGATTTAT  4258
5-AE009948        5187 CTTGAGCAAACTTATCAGAATTTAGAGATTATCA-TAA------TTGATGATGGCTCTAG  5239
6-HF952106        3548 CTTCAACAAACATACCCAGAAATAGAAATTTT-ATTAA------TAGATGATGGATCTAC  3600
7-AY376403        4271 ATGGCCACAACTTATTCAAATCTAGGAAATATTAATTG------TTG-TGATGGCTCAAC  4323
8-AY375363        3531 ATATAAGAAGTGTA---ATTGGTTGGAATGG--AACAG------TTGA-GATGCCTTTAT  3578
9-CGBY01000002    4310 ATAGCGCAGACCTACCCCAATTT-GGAAATATTAATCA------TTGATGATGGGTCAAC  4362
                                    *                              *     **   *  *

1a-CP000114       3713 TGATAATAGTGGAGAAATTT---GTGATAATTTATCTCAAGAAGATAATCGCAT-ACTTG  3768
1b-FO393392       3713 TGATAATAGTGGAGAAATTT---GTGATAATTTATCTCAAAAAGACGATCGCAT-ACTTG  3768
2-AAJO01000077    4135 CGATGGTTCAGGAGACTATT---GTGATGAAATTGCCAAAAAAGATAGTCGAAT-TTTTG  4190
3-AL766849        3602 TGATAATAGTGGAGGAATTT---GTGATAATTTATCTCAGGAAGATAATCGCAT-ACTTG  3657
4-AF355776        4259 TGGTCATAGTCGAAAACTTTC-TGTGATAGTTTTCTTATTTTTTCCATTTCTAT--ACAA  4315
5-AE009948        5240 TGATCGCACAGGGGATATTT---GCGAAAAGTATTTTTTGGAAGACAGGCGAATAAAATA  5296
6-HF952106        3601 AGATAAAAGTAGTCATATTT---GTAATAATTTTTTAAAAAGGGATAGTCGCGT-AAAAG  3656
7-AY376403        4324 TGATTACACATCAGTTATAT---GCCAAAAATACACTTCTAAAGATAAACGCGTGAAATA  4380
8-AY375363        3579 TAAGTATAATGATAAAAAATGGCTTCATTGGATTGTTAGGATATACAGTTGT-TCTACTC  3637
9-CGBY01000002    4363 TGATAATACATCAACTATAT---GTCAGAAGTATGTTTCTAAAGATAAGCGCATGAAATA  4419
                                          *      *                          *

1a-CP000114       3769 TATTTCATAAAAAAAAT---GGAGGGGTCTCTTCGGCAAGGAACCTAGGTCTAGATAAAT  3825
1b-FO393392       3769 TATTTCATAAAAAAAAT---GGAGGGGTATCTTCGGCAAGGAACCTAGGTCTTGATAAAT  3825
2-AAJO01000077    4191 TGTATCATAAGACAAAC---GGCGGTCTATCAGAAGCTCGAAACGTTGGAATAAAAATAA  4247
```

```
3-AL766849       3658 TATTTCATAAAAAAAAT---GGAGGGGTCTCTTCGGCAAGGAACCTAGGTCTAGATAAAT  3714
4-AF355776       4316 AATTTTATTAGAAAAATATAGAGGGAGAGTACAATGATAGAAA-----AAACTATGATAC  4370
5-AE009948       5297 TTTCT-ATCAAGAAAAC---AGAGGCCAATCAGTTGCACGTAACAACGGAGT--ACTTAG  5350
6-HF952106       3657 TCTATCATAAATACAAT---GGAGGTGTATCATCAGCAAGAAATGTGGGACTTGAGATGG  3713
7-AY376403       4381 TTTAT-ATAAAGAAAAT---GGCGGTCAATCCTCTGCTAGAAATTTAGGAGTCAAACTAG  4436
8-AY375363       3638 TCTTTTTTTAAAAATAT----AAAGAAAAT-AAAAAATATAAACATAAAAACTG-TAGGA  3691
9-CGBY01000002   4420 TATAT-ATAAAAAAAAT---GGAGGTCAATCTTCTGCTAGAAATTTAGGCGTTAAACTAT  4475
                        * *  * *     *        *    *           **

1a-CP000114      3826 CCA--CAGGAGAATTCATAACATTTGTGGATAGTGATGATTTTGTAGCAC-CGAA-TATG  3881
1b-FO393392      3826 CCA--CAGGCGAATTCATAACGTTTGTAGATAGTGATGATTTTGTAGCAC-CGAA-TATA  3881
2-AAJO01000077   4248 GTA--CCGGGAAATATATTACATTTATAGATTCAGACGATTACATCGAAAATTTA-TAT-  4303
3-AL766849       3715 CCA--CAGGAGAATTCATAACATTTGTGGATAGTGATGATTTTGTAGCAC-CGAA-TATG  3770
4-AF355776       4371 CTA----AAAAAATACATTA---TTGTTGGTT-TGGTGGTAATCCTAAGAGTGAACTGTT  4422
5-AE009948       5351 GTGTACAGGAGATTGGATTGCTTTTTTAGACAGTGATGATGTTTATCTTC-CTTA-TTCA  5408
6-HF952106       3714 CAG--AAGGTGAATTTATAACTTTTGTAGATAGCGATGATGTTGTCGCAC-TAAA-TATG  3769
7-AY376403       4437 CTG--AAGGAGATTGGATTGCTTTCTTGGATTCCGATGACCTCTGCTCAC-C--A-TTCT  4490
8-AY375363       3692 TTA--CTAGTGATTATCCCA---TTATTGATGTCGGCAACTGTTGAGAAC------TAT-  3739
9-CGBY01000002   4476 CTA--CAAGTAATTGGATAGCTTTCTTAGATTCTGATGATCTTTGTTCGC-C--A-TTTT  4529
                                 * *         *  * *     *                     *

1a-CP000114      3882 ATTGAAATAATGTTA-AAAAATTTAAT-CACTGAGAATGCTGATAT--------------  3925
1b-FO393392      3882 ATTGAAATAATGTTA-AAAAATTTAAT-CACTGAGGATGCTGATAT--------------  3925
2-AAJO01000077   4304 ATTGAAACTTTATAC-AACAGTTTAAT-TGAGTATAAGGCTGATATTGCTATTGTTAATA  4361
3-AL766849       3771 ATTGAAATAATGTTA-AAAAATTTAAT-CACTGAGAATGCTGATAT--------------  3814
4-AF355776       4423 ATTAAAA---TGTAT---CAAATCA---TGGGAAAAATATTGTCCAG-------------  4460
5-AE009948       5409 ATTGAGGTTATGTAC--AACATACAGAAAGCTACCAATGCCGATATT-------------  5453
6-HF952106       3770 ATTGAAATTATGCTG-AATAATTTGTT-AACGGAGAACGCAGATAT--------------  3813
7-AY376403       4491 ATATAGAATATTTAGTAAGAGTGCAGAGTGAAACTGGTGCTGATTTG-------------  4537
8-AY375363       3740 ATCGTAA--ATGTAA-----ATTTTAT-----ATTTATGCCAATTT--------------  3773
9-CGBY01000002   4530 ATATAGAATGTCTAGTGAGAATTCAACATGAAACTGGTGCTGACCTT-------------  4576
                      **        *

1a-CP000114      3926 ---AGCA----------------GAAGTAG---ATTTTG-ATATTTCGAATGA------G  3956
1b-FO393392      3926 ---AGCA----------------GAAGTAG---ATTTTG-ATATTTCGAATGA------G  3956
2-AAJO01000077   4362 AATATCATTTAAAGGATAACGAGAAAATTGTTCATTTGGCAAATTCCAAAGAACTTTTTA  4421
3-AL766849       3815 ---AGCA----------------GAAGTAG---ATTTTG-ATATTTCGAATGA------G  3845
4-AF355776       4461 ---ATTAC---------------GAAATTATTGAATGGA-ATGAACAAAAT---------  4492
5-AE009948       5454 ---GTTTT----------------AACTAGT--ATTGGTAACTTCAACAATA--------  5484
6-HF952106       3814 ---ATCA----------------GAAATTG---ATTTCG-AAGTTTCAGATGATTTTT--  3848
7-AY376403       4538 ---GTGAT----------------AACTGGA--ATCGAAGACCGCCCCAAC---------  4567
8-AY375363       3774 ---GTTT-------------------TTGT--GTTCTAAACTCTCTTAAAAG-------  3801
9-CGBY01000002   4577 ---GTAAT----------------AACTGGA--ATTGAGGATCACCCTTGT---------  4606
                                                 *            *
```

```
1a-CP000114       3957 AGAGATT--ATAGAAAGAAGA----AAAGACGAAAC-TTTTATAAAG------TCTTTAA  4003
1b-FO393392       3957 AGAGATT--ATAGAAAGAAAA----AAAGACGAAAC-TTTTATAAGG------TCTTTAA  4003
2-AAJO01000077    4422 GGGTATTTGATACTAAAATGATTTTAGAGAGTATGTATAGTAGAGAGAATAATTTTATTA  4481
3-AL766849        3846 AGAGATT--ATAGAAAGAAGA----AAAGACGAAAC-TTTTATAAAG------TTTTTAA  3892
4-AF355776        4493 ----------TATGATGTCAAT----AAAATTACATATACTAGACAAG-----CTTATAA  4533
5-AE009948        5485 --------CTTACAATACTAGT----ATTAATTCACAGTATTTAAAA------GAGATTA  5526
6-HF952106        3849 ---------ATAAAAGAAAAA----AAAGAAAAGGT-TACTATAGAG------TTTTTCA  3888
7-AY376403        4568 --------CATAGAATGGAAA--------AACTTACATGTGATAAGT------CAACTTA  4605
8-AY375363        3802 ---------ATTAGAACACAAT--------TTAAACGTATAAGGATATA--ACTTTATTA  3842
9-CGBY01000002    4607 --------TACAGAACGGCAA--------AACTTTCATTTGATAAAC------TATCTTA  4644
                                     *                                          * *

1a-CP000114       4004 A------AACAAT-----------AACTC---TTTAAAAGAATTTTTATC----------  4033
1b-FO393392       4004 A------AACAAT-----------AATTC---TTTAAAAGAATTTTTATC----------  4033
2-AAJO01000077    4482 GTACCCTGACAATGGCGCAGC-TAAAATTATATCGTAAGGAACTTTTCTCTGATATAGAA  4540
3-AL766849        3893 G------AATAAT-----------AACTC---TTTGAAAGAATTTTTATC----------  3922
4-AF355776        4534 AGAAGGAAAGTATGCTTTCGTGTCTGATTATGCTAGATTGGATATTATTT----------  4583
5-AE009948        5527 AA-----CTTTAT------------ACTT----TAGAGGTGGCGTTGGA-----------  5554
6-HF952106        3889 A------AACAAT-----------AAGTC---TCTCAAAGAATTTTTTTC----------  3918
7-AY376403        4606 -------CTCAAC------------ACTCAGTGTGGAAGCGACATTAAC-----------  4635
8-AY375363        3843 T-------GCAA-------------AATC----TAGTAAGTATTATTCTACCT-------  3871
9-CGBY01000002    4645 -------CTCAGT------------ACTCAGTGTGGAAGAGACACTAAT-----------  4674
                                                  *                 *

1a-CP000114       4034 ---------AGGCA-ATAGAGTGGAAA--------ATATTGT------------------  4057
1b-FO393392       4034 ---------AGGTA-ATAGAGTGGAAA--------ATATTGT------------------  4057
2-AAJO01000077    4541 TTTCCCATTGGGAA-AAAATATGAAGA--------TTCTTTTACAACTTATAAGACATAC  4591
3-AL766849        3923 ---------AGGTA-ATAGAGTGGAAA--------ATATTGT------------------  3946
4-AF355776        4584 -----------ATCAATATGGTGGGAT--------ATATTTAGA----------------  4608
5-AE009948        5555 ---------AGAAATGTATTATGGGAAAACTTATGGAGTTTCCC---------------C  5590
6-HF952106        3919 ---------AGGAA-ATAAAGTAGAAA--------ATGTTGT------------------  3942
7-AY376403        4636 ---------AGAAATGTACTATGATAA---CTACGGTATTTCTC---------------C  4668
8-AY375363        3872 -----------GTTTATAATGTAGAAA----------ATTAT------------------  3892
9-CGBY01000002    4675 ---------AGAAATGTATTATGATAA---CTATGGTATTTCTC---------------C  4707
                                        *   *                **

1a-CP000114       4058 -TTGTACAAAAT---------TATATAAAAAAAGTATAATT-----GGCAACTTGAGGTT  4102
1b-FO393392       4058 -TTGTACAAAAT---------TATATAAAAAAAGTATAATT-----GGTAACTTGAGGTT  4102
2-AAJO01000077    4592 ATTGAAGCTAATAGGATTGTTTTTGTTAATAGACCATTAT-------ATGCCTATCGATT  4644
3-AL766849        3947 -TTGTACAAAAT---------TATATAAAAAAAGTATAATT-----GGTAACTTGAGGTT  3991
4-AF355776        4609 --TACAGATGTTGA--------ACTTATAAAACCTATTGAC-----GACCTTTTAAATTG  4653
5-AE009948        5591 GTTAGCAAAATTA--------TATCCGAGAAGTAATTTATT-----GAGTAATCCATATC  5637
6-HF952106        3943 -TTGGGGGAAAT---------TATATAAAAAAAGCATTATT-----GGGGATTTACGATT  3987
```

```
7-AY376403       4669 ATTGGGAAAGTTA--------TACTCTAAAGCATTATTATT-----AAGACATCCATACC  4715
8-AY375363       3893 ---ATTGAAAAT-----------TGTTTAAACTCCATTATTTCTCAGAGTTATTTTCATT  3938
9-CGBY01000002   4708 ATTAGGGAAGTTA--------TATTCCAAAGGGTTATTATT-----AAAATATCCATACC  4754
                                 *                        *               *

1a-CP000114      4103 T---GATGAGA--ACT--TAAA-AAT-TGGTGAGGATTTACTT----TTTA---ATTGTA  4146
1b-FO393392      4103 T---GATGAGA--ATT--TAAA-AAT-TGGTGAGGATTTACTT----TTTA---ATTGTA  4146
2-AAJO01000077   4645 TCGAAGTGGGAGCATTA-CAAG-AAG-TGGTTATCAATTATCAAA--TTTAGACGTTTTA  4699
3-AL766849       3992 T---GATGAGA--ACT--TAAA-AAT-TGGTGAGGATTTACTT----TTTA---ATTGCA  4035
4-AF355776       4654 TGAAAGTTATTTTGCTTGTGAGCTACCTGGAGAGG--TTAATACAGGTTTG--GGTTTTG  4709
5-AE009948       5638 CA--GAAGGAAAAATTCATGAA-GATATGGATACAACTTTTAAG---CTTA----TATCT  5687
6-HF952106       3988 T---AATGAAA--AATA-CAA--AAT-TGGTGAAGACTTGCTA----TTTA-AC-TTTCA  4032
7-AY376403       4716 CT--ATTGGTAAAATTCATGAA-GATACAGATACAACTTACAAA---CTGG----TTGCA  4765
8-AY375363       3939 TT--GAAGTAATTATTG-TGAATGAT---GGATCGACTGATAA------TA----GTATG  3982
9-CGBY01000002   4755 CT--ATTGGTAAAATTCATGAA-GATACAGACACAACTTATAAA---CTGA----TTGCA  4804
                                     *    *   *    *       *

1a-CP000114      4147 AAC-----------------------TCTTATGTCAAGAG--CACCGTATAGTCGTAGAT  4181
1b-FO393392      4147 AAA-----------------------TTTTATGTCAAGAG--CACTGCATAGTCGTAGAT  4181
2-AAJO01000077   4700 GAAATGTACGAAGAGCGGTTGGAATATTTAGAGAAAGAGGATATGATTTACTT---AGT  4756
3-AL766849       4036 AAC-----------------------TCTTATGTCAAGAG--CACCGTATAGTCGTAGAT  4070
4-AF355776       4710 GA-------------------------TCAATTTCAAGAAATCCTTTTATAAAAGAAAAT  4744
5-AE009948       5688 TG------------------------CGCTTCAAAAATAGCAGTTTGTGATATTGTCACT  5723
6-HF952106       4033 GA------------------------TTTTAAATAAAGA--ACATCGTATAGTTGTAGAT  4066
7-AY376403       4766 AG------------------------TAGTACACGAATTGCGTTTAGCAAA---GAGAAA  4798
8-AY375363       3983 AGC-----------------------TACTGTGAAAAAA-----TAGTATG--CGAAGAT  4012
9-CGBY01000002   4805 AG------------------------TAGTACACGAATTGCATTGAGCAAA---GAGAAA  4837
                                                         **

1a-CP000114      4182 AC--GACTTCTTCCTTATATAC----TTATCGA---------------ATTGTAAAAACT  4220
1b-FO393392      4182 AC--GACTTCTTCCCTGTACAC----CTATCGC---------------ATCGTAAAGACT  4220
2-AAJO01000077   4757 ACTAAATATCATTATTGTATAG----CTATTGCAAATCAAAAAGAGTTATTAAAAAAACA  4812
3-AL766849       4071 AC--GACTTCTTCCTTATATAC----TTATCGA---------------ATTGTAAAAACT  4109
4-AF355776       4745 ATGAAAATCTACGAAGATACAGAATACTATAGTT---------------TAAAAAAAACG  4789
5-AE009948       5724 GC---AGTGGTGTATTTTAG------CGA---------------------TAATAGTACT  5753
6-HF952106       4067 ACTAGAAG--ATCACTCTATAC----TTATCG---------------TATTGAAGAAAAA  4105
7-AY376403       4799 AC---AGTA-TATATAAAAGGT----CAACC-------------------TAATAGTACG  4831
8-AY375363       4013 TT-AAGGTTTAGAATTATAAAC-------------------------------AAAGAT-  4039
9-CGBY01000002   4838 AC---AGTA-TATGTCAAAGGG----CAACC-------------------TAATAGTACT  4870
                                        *                                      *

1a-CP000114      4221 TCCGC--AATGAATCAGAAATTCAACGAAAACTCA------TTAGAT---TTTATAAC--  4267
1b-FO393392      4221 TCTGC--AATGAATCAGGAGTTCAACGAAAATTCA------TTAGAT---TTTATAAC--  4267
2-AAJO01000077   4813 TTTCCCTAATAATCCTAAAATTTATAAATTGGACAGCCAGTTTAGAGAAATATATTGTGA  4872
```

```
3-AL766849       4110 TCCGC--AATGAATCAGAAATTCAACGAAAACTCA------TTAGAT---TTTATAAC--  4156
4-AF355776       4790 TGTGTGGCAATTACCACAGATTTACTAACAGAGCAAG-GGCTTAAGT----ACATAAATA  4844
5-AE009948       5754 ACT--------AGGACAAAATTCAATGAGAGAATG------TTG--------TATTT---  5788
6-HF952106       4106 TCTA--TAATGAATCAACAATTTAATAAAAATACA------TTAGAC---TTCATTG--A  4152
7-AY376403       4832 ACT--------AGGCAGTCATATAGGGAGAACATG------TTA--------TATTA---  4866
8-AY375363       4040 ------------AATGGTGGTCTATCTGATGCGCG-------TAA-------TGTAGG--  4071
9-CGBY01000002   4871 ACC--------AGACAGTCATACAAAGAGAATATG------TTA--------TATTA---  4905
                                          *  *                  *           *

1a-CP000114      4268 AATTTTTAATGAAGTAAGTAGTTTGGTTCCTGCCAAATT--GGCTAATTATGTTGAAGCG  4325
1b-FO393392      4268 AATTTTTAATGAAATAAGCAGTATTGTTCCTGCAAAATT--AGCTAATTATGTTGAAGCG  4325
2-AAJO01000077   4873 TATTTTAAATGAACTTCCCTTTAAAAAGCGAATAAAATTAGGAATTATTAGGTTCTTACC  4932
3-AL766849       4157 AATTTTTAATGAAGTAAGTAGTTTGGTTCCTGCCAAATT--GGCTAATTATGTTGAAGCG  4214
4-AF355776       4845 AAATTCAAGTAATAAACGGTATTTTAATTTATCC---TCCAAGTTATTTTTGTCCAGTAA  4901
5-AE009948       5789 ---TTTTGAGGCAATT-------------CAAAATAATATTGTAT--TTATAAATCTAAA  5830
6-HF952106       4153 TATTTTTAATGAGATTCATCAGGATAGTCCGACAGAATT--GTTTAATTATGTGGAAGCG  4210
7-AY376403       4867 ---TTTTGATGCAATG-------------CAACATAATAGAGACT--TTATTCTTGAACG  4908
8-AY375363       4072 ---TTTAGAAG------------------CAGCAAAAGGGGACTATATTATTTTTGTAGA  4110
9-CGBY01000002   4906 ---TTTTGAAGCAATG-------------CAACATAATAGAGATT--TTATCCTTGAACA  4947
                         **                                           **

1a-CP000114      4326 AAATTTTTAAGAG--AAAAGATAAAGT-GTCTCCGAAAAATGTT--------TGAATTAG  4374
1b-FO393392      4326 AAATTTTTAAGAG--AAAAGGTAAAGT-GTCTCCGAAAAATGTT--------TGAATTAG  4374
2-AAJO01000077   4933 ATTTATTAAACGGTTAAAATACCAATTTATTGAAAGTAGATGATATGCAACATATATTAG  4992
3-AL766849       4215 AAATTTTTAAGAG--AAAAGATAAAGT-GTCTCCGAAAAATGTT--------TGAATTAG  4263
4-AF355776       4902 ATATGTT--------AACAAATAAAAT-AAATATAAAAGAGGAA---------ACATTAA  4943
5-AE009948       5831 TTTTCC------------ACATAATAC-ATCTTTAATCTCTGCTG-------TAATATA-  5869
6-HF952106       4211 AAGT-TTGTACGAGAAAAA-ATCAAGT-GTTTAAGGAAAATGTT--------TGAATTAG  4259
7-AY376403       4909 TTATTC------------AGACAAAGA-AC---TAATCGATGCTT-------TAAATTA-  4944
8-AY375363       4111 TAGC---------------GATGATTTCAT---------ATCTCC-------TAACTTAG  4139
9-CGBY01000002   4948 TTATTC------------AGATAAAGA-AC---TAATTGATGCTT-------TAAATTA-  4983
                                             *                                 **

1a-CP000114      4375 --------------------------GTAGTAATATTGACAATAAAATCAAAGTACAACG  4408
1b-FO393392      4375 --------------------------GTAGTAATATTGACAGTAAAATCAAATTACAACG  4408
2-AAJO01000077   4993 CAATTATTAGTTATAAAATGACACATGCATTAAAATGTACCGTAGATTTATTGAAAAATG  5052
3-AL766849       4264 --------------------------GTAGTAATATTGATAATAAAATCAAAGTACAACG  4297
4-AF355776       4944 C-------------------------GATTCACCATTATGGACGAAATTGGGGAGGAGAA  4978
5-AE009948       5870 ------------------------------TAATGAAG-TATTTGGTGGAATAGATATAT  5898
6-HF952106       4260 GAG------------AAATAGCTGATG----AAAATTTAC-----GTTTACAGA-----G  4293
7-AY376403       4945 ------------------------------TAAAACTG-TAATTGGAGGAATGTGGATCG  4973
8-AY375363       4140 ---------------------------TTTCTCATCTCATGGCATGTCTGGAAAATTATG  4172
9-CGBY01000002   4984 ------------------------------TAAAACTG-TAATTGGAGGAATGTGGATCG  5012
```

```
1a-CP000114      4409 AGA--GATTTT--------------TTTCAAA---GAC---ATTAAATCATA--CCCGTT  4444
1b-FO393392      4409 AGA--GATTTT--------------TTTCAAA---GAT---GTTAAATTATA--CCCTTT  4444
2-AAJO01000077   5053 ATATGGATATAGTTATACACGTTGATGCCAAAAGTGAT---ATAAAACAATA--TG-ATT  5106
3-AL766849       4298 AGA--GATTTT--------------TTTCAAA---GAC---ATTAAATCATA--CCCGTT  4333
4-AF355776       4979 AAAAAAATA-----------------ATTGATTTAGGTAGAATAAAGTTAGGGGTTCATT  5021
5-AE009948       5899 GTGGGAAGATG---------------ATTGATTTTAAAT-TATATGATACAG--TTGATT  5940
6-HF952106       4294 ATATAAATTT---------------TGGCAA----GAT---ATTAAATCATA--T----T  4325
7-AY376403       4974 TTAATAAGCTT---------------CTAGAAGTTGATA-AATACGATTATA--TTAATT  5015
8-AY375363       4173 AAGCGGATCTT---------------GCAATTTGTGATCCAGTTCATTTTTA------TT  4211
9-CGBY01000002   5013 TTAATAAGCTT---------------TTAGAAGGAGATA-AGTACGATTATA--TTAATT  5054
                            *                                   *                *

1a-CP000114      4445 CTATAA-------------------AGCGGTAAA--------------------------  4459
1b-FO393392      4445 CTATAA-------------------AGCGGTTAA--------------------------  4459
2-AAJO01000077   5107 CGATAAAAGAATTTGTAACTTTCACAGAAGAAAGAATAGATGTTCGTTGGGGACACTATT  5166
3-AL766849       4334 CTATAA-------------------AGCGGTAAA--------------------------  4348
4-AF355776       5022 TACTAC--------------------TAGATAAG--------------------------  5035
5-AE009948       5941 ATTACA--------------------GAAAAAAA--------------------------  5954
6-HF952106       4326 CAATA--------TGCAA-------AGCAATAAG--------------------------  4344
7-AY376403       5016 CTTTGA--------------------GAAGTGAA--------------------------  5029
8-AY375363       4212 TGG-----------------------GAGGTAATC-------------------------  4223
9-CGBY01000002   5055 CTTTGA--------------------GAAGTGAA--------------------------  5068
                                                      *

1a-CP000114      4460 --------------------ATACTT--------------ATC--ATTAAAGGGATTATT  4483
1b-FO393392      4460 --------------------GTACTT--------------ATC--ATTAAAGGGATTATT  4483
2-AAJO01000077   5167 CTCAGATTTTAGCTATGTTAGTTCTTTTAAAATATATGAAGTCCAAAGAAAAATATCATT  5226
3-AL766849       4349 --------------------ATACTT--------------ATC--ATTAAAGGGATTATT  4372
4-AF355776       5036 --------------------ATATTTGGAT---------------ATGGAACGTACAGAA  5060
5-AE009948       5955 ------------------------T--------------------ATAGAAAATATTTTA  5970
6-HF952106       4345 --------------------GTTCTT--------------ATCTAAA-AAACATATCTGT  4369
7-AY376403       5030 ------------------------T--------------------ATAGAAAATATTACA  5045
8-AY375363       4224 ---------------------------------------------AAGAGACACACGAGA  4238
9-CGBY01000002   5069 ------------------------T--------------------ATAGAAAATATTATA  5084
                                                                   *    *   *

1a-CP000114      4484 AAGCTTTTAT--TTAATGAAAT---GTTCACCTAAACTATATGTTATGGCATATAGAAGA  4538
1b-FO393392      4484 GAGTATTTAC--TTAATGAAAT---GTTCACCCATCTTGTATATAAAATTATATGACAGG  4538
2-AAJO01000077   5227 ATATTTCTATTATTAGTGAAAGCGACATCCCATTAAAAACAGGTAAAGAAAT-TAATACT  5285
3-AL766849       4373 AAGCTTTTAT--TTAATGAAAT---GTTCACCTAAACTATATGTTATGGCATATAGAAGA  4427
4-AF355776       5061 ATATT-------TTAAAGGCAG-----------TAAAAAAATAGTAAGGTAT-CAAAGGT  5101
5-AE009948       5971 AGACTATTCTCTTTAATAATAG--GATTAGTGTGAAGGAGAAGGTTAAATATATATTATT  6028
6-HF952106       4370 ACGTT---ATATTTGATGAAAT--ATTTTCCGT-ACGTATATATAAAGATGTATAATAAA  4423
```

```
7-AY376403       5046 AGAGTATATTATTTAATAAACG--TGTGACAATAAAGAAAAAATAAAATATCTGTCATT  5103
8-AY375363       4239 AAAAT---ATTTTTAAAAAACA-----TTCAAACATTCATTTATTAACAAATGAAGAAGC  4290
9-CGBY01000002   5085 AGAGTATATTATTTAATAAGCG--AGTTACAATTAAAGAAAAAATAAAATATTTTTCATT  5142
                                  **                                     *

1a-CP000114      4539 TTTCAAAAACAGTA-GCTGG--AGAAATTGGGAAAGAGAATTTATAACT-TCATGA-T--  4591
1b-FO393392      4539 TTTCAAAAACAGTA-AGTAATCAAAAATT---AAATTAACTCAATTACC-TTTTAAAT--  4591
2-AAJO01000077   5286 TTTTTAACCCTTCATAAAGGTAAAGAATTCATAGGTATGGTTTCTAACG--AGCAAAT--  5341
3-AL766849       4428 TTTCAAAAACAGTA-GCTGG--AGAAATTGGGAAAGAGAATTTATAACT-TCATGA-T--  4480
4-AF355776       5102 AACCTGATTATTCATTTAAAT-AGTAATCTT----GTCAACTAATAATT--TATAGGA--  5152
5-AE009948       6029 TATTTCCTCTATAAGATATTT-CACTATTGT-AAGAAAAAATATATAATT----TGAGGTT 6082
6-HF952106       4424 TTTCAAA--------AGCAATAACTAAGTCATA-----------CAAT---ACTAGGA--  4459
7-AY376403       5104 TATTTCCTCAATGA-ACCTGT-TTTTATTCTTAAGAAAGATTTACAACTATAGTGAGG--  5159
8-AY375363       4291 GATTTG------TGAACTTTT------TTATCAAAAAACATTTTTAGTT-----------  4327
9-CGBY01000002   5143 TATTTCGTCAATGA-ATTTAT-TTTTATTTTTAAAAAAATTTTATAACTATAATGGGA--  5198


1a-CP000114      4592 AAAGAAAATTGAAAAGGATTTAAT-AAGTGTAATTGTACCTATTT---------------  4635
1b-FO393392      4592 TATAGGAGTTGAAAATGAATTA---TAGTATCATTATGTCGGTAT---------------  4633
2-AAJO01000077   5342 GAGTGATTTTGAGTATGATAAACTCAAGTATAACTATCCTGATTTTTGTTTTTCTAAAAG  5401
3-AL766849       4481 AAAGAAAATTGAAAAGGATTTAAT-AAGTGTAATTGTACCTATTT---------------  4524
4-AF355776       5153 -AGTAATAATGGAAAATATTTTAATTAGTATAATTATTCCGATAT---------------  5196
5-AE009948       6083 ATCAAGGAATGGATAAAGTT------AGTATCATTATCCCAGTTT---------------  6121
6-HF952106       4460 GGATAATTTGAAGTAT----------AGTGTCATCATGTCAATTT---------------  4494
7-AY376403       5160 GATAAATTATGAAAAAGATC------AGTGTTATTATCCCCGTTT---------------  5198
8-AY375363       4328 --TCGGCTTGGGGAAAAGTT--------TATAAAAAGTCTATTTTT--------------  4363
9-CGBY01000002   5199 GATAAATTATGAAGATGATT------AGTATCATTATCCCAGTTT---------------  5237
                                    *             * * *         * *

1a-CP000114      4636 ----ATAATG---------TTGAAGATTAT-----TTAGTTGAATGTATAG-------AA  4670
1b-FO393392      4634 ----ATAATGAG------CCTTTAAATTAT-----GTGAGAGATTCAGTAG-------AA  4671
2-AAJO01000077   5402 AGGAATATTTAAAAAAATTTATAGAGGTACCAAAGTTTATAGGTTCTTTAA-AAATGCAA  5460
3-AL766849       4525 ----ATAATG---------TTGAAGATTAT-----TTAGTTGAATGTATAG-------AA  4559
4-AF355776       5197 ----ATAATG---------TAGAAAAATTC-----TTAGAGGAATGTATTG-------AT  5231
5-AE009948       6122 ----ATAACG---------TTCAATCTTTC-----CTAAACGAGTGTATAG-------AA  6156
6-HF952106       4495 ----ATAATGAA------CCATTGAAGTAC--------ATTAAATCTTCAATAGATTCAA  4536
7-AY376403       5199 ----ACAATG---------TTCAATCTTTT-----CTAAATGAATGTATAG-------AT  5233
8-AY375363       4364 ---GATAAT----------------------------ATTAGGTTTCCAA-------AA  4384
9-CGBY01000002   5238 ----ATAATG---------TTCAATCTTTC-----CTAAACGAGTGCATAG-------AA  5272
                          * *                                     *             *

1a-CP000114      4671 AGCCTTATTGT-T------------CAAA-----------------CATATCGAAA----  4696
1b-FO393392      4672 T-CTATATTAAAT------------CAAA-----------------CGCTTACTGA----  4697
2-AAJO01000077   5461 GGTATTATTCCCTACCTCCTCTTCGTAAGGGAAGTCAGTGGTTTACTATTTCAAGAGAGT  5520
```

```
3-AL766849        4560 AGCCTTATTGT-T------------CAAA-----------------CATATCGAAA----  4585
4-AF355776        5232 AGTGTTTTAAA-------------TCAAA-----------------CTTATAAAAA----  5257
5-AE009948        6157 AGTGTTTTAGC-------------TCAAA-----------------CTTATTCAAA----  6182
6-HF952106        4537 --TTTTAT---------------CACAA-------------------ACATTAAAAAACT  4560
7-AY376403        5234 AGTGTTTTGGC-------------TCAAA-----------------CGTATTCAAA----  5259
8-AY375363        4385 GGT-----------------------AAG----------------TTATTTGAAGA----  4401
9-CGBY01000002    5273 AGTATTTTAGC-------------TCAAA-----------------CTTATTCAAA----  5298
                                                 **                      *    *

1a-CP000114       4697 -TATTGAAAT-AT-TATTGATTAAT--GATGG----TTCGAC-------GGATAATTGTG  4740
1b-FO393392       4698 -TTTTGAGTT-CA-TAATTGTCATT--GATAA----TCCAAGTA----GAGGTGATT-TA  4743
2-AAJO01000077    5521 TTACTGACTGGAT-TATTGGATATTTAGATAAACACCCAGATTATCAG-GAGGCTTTTTG  5578
3-AL766849        4586 -TATTGAAAT-AT-TATTGATTAAT--GATGG----TTCGAC-------GGATAATTGTG  4629
4-AF355776        5258 -TATAGAAAT-AC-TGTTAGTAGAT--GATGG----TTCAAC-------GGATAATTCAG  5301
5-AE009948        6183 -TTTAGAAATTAT-T-CTAGTTAAC--GATGG----CTCTAC-------AGATAATTCCG  6226
6-HF952106        4561 TT---GAATTTAT-TATTGTG-ATT--GATAA---CCCTGA----CAGAGAAGATGTATA  4606
7-AY376403        5260 -TTTAGAAATTAT-T-TTAGTTGAT--GATGG----CTCTAC-------GGATACTTCTG  5303
8-AY375363        4402 -TAGCGCAGTAATGCATTTATTATT----TGA-----------------GAAAAGCAATA  4439
9-CGBY01000002    5299 -TTTAGAAATTAT-T-CTAGTTAAC--GATGG----CTCTAC-------AGATAATTCCG  5342
                        *   *           *           *

1a-CP000114       4741 CGACA-------------------ATTGCCAA-AGAATTTTCTGAAA-------------  4767
1b-FO393392       4744 AAGCA-------------------ATTCTTAACAGAATATTCAGTTG-------------  4771
2-AAJO01000077    5579 TCATAGCTATTGTGGAGATGAATTATTTTTTCAAACATTACTAGCTATCTCTCCATTTCA  5638
3-AL766849        4630 CGACA-------------------ATTGCCAA-AGAATTTTCTGAAA-------------  4656
4-AF355776        5302 GAAT---------------------TATCTGTGATAATTATAGCCTG-------------  5327
5-AE009948        6227 GGG---------------------ATATCTGTGATTATTATTCAGAA-------------  6252
6-HF952106        4607 TGATA---------------------TTTTGCAAACATATCTAGCAA-------------  4632
7-AY376403        5304 GGG---------------------ATATCTGTGACTATTATTCAAAA-------------  5329
8-AY375363        4440 AGAT---------------------TGTTTATTCGAAT-----GCAA-------------  4460
9-CGBY01000002    5343 GGG---------------------ATATCTGCGATTATTATTCGGAA-------------  5368
                                                           **

1a-CP000114       4768 -------GAGATTGTAGAGTTA-----TCTATATAGAAA-AGTCGAATGG------TGGG  4808
1b-FO393392       4772 -------TAGATAATAGAATAAAAAT-CTTGCTTAATGA-AGA-AAATAT------TGGT  4815
2-AAJO01000077    5639 AGAAAGGGTGATG-AATAATAAAGATGACTGTCTCATGGGAGGGAGATATATTATTTGGG  5697
3-AL766849        4657 -------GAGATTGTAGAGTTA-----TCTATATAGAAA-AGTCGAATGG------TGGG  4697
4-AF355776        5328 ------AAAGATAAACGAATCA----GGGTATTTCATAAGAAT--AATGGA------GGT  5369
5-AE009948        6253 ------ATAGATGGGCGTATA----TTCGTTTTTCACAAAAAT--AATGG------TGGT  6294
6-HF952106        4633 -------GCGATGCAAGAGTAAAAGT-ATTATCAAATGAGAG--AAATAT-------GGG  4675
7-AY376403        5330 ------AAAGATAGACGTATA----TTAGTTTTTCATAAGAAT--AATGG------TGGC  5371
8-AY375363        4461 --------AACTGTACGCATA--------TGT-TCATAGGAAA--GATAG------TATT  4495
9-CGBY01000002    5369 ------ATAGATGGGCGTATA----TTCGTTTTTCACAAAAAA--AATGG------TGGT  5410
                                  *       *         *          *     **
```

```
1a-CP000114       4809 --------------TTGTCAGAAGCTAGA------AACTATGGAATTTATCATTCAAAGG  4848
1b-FO393392       4816 --------------TTAGCATCAAGTTTG------AACAAAGCGGTGAAAATTTCTGAGG  4855
2-AAJO01000077    5698 AGGCTGATAAGACATCACCTTGCGATTTATTTGCTGAAAAAGTGTTAGAATATAGGAAAG  5757
3-AL766849        4698 --------------TTGTCAGAAGCTAGA------AACTATGGAATTTATCATTCAAAGG  4737
4-AF355776        5370 --------------TTATCCGATGCTCGG------AATTTTGGTGTAGTAAATGCTGAAG  5409
5-AE009948        6295 --------------TTATCAGATGCTCGT------AATTATGGAATTAGTAGAGCTACTG  6334
6-HF952106        4676 A-------------TTAGCTTATAGTTTA------AACAAAGCTATAAAATATTC-----  4711
7-AY376403        5372 --------------TTATCTGATGCCAGA------AAAAAAGAAAATAGTAGAGCTACTG  5411
8-AY375363        4496 A-------------CTACTAGTCAATTTT----CTGAACGTGATTTAGACATTTTAGATA  4538
9-CGBY01000002    5411 --------------TTATCAGATGCTCGT------AATTATGGAATTAGTAGAGCTACTG  5450
                                                           *    *

1a-CP000114       4849 -----GAAAATAC-TTAACT--TTTG--TTG-ATTCAGA-----TGATAAAGTTTCCT--  4890
1b-FO393392       4856 -----GAGAATA---TATTT--TTAGAATGG-ATGCTGA-----TGATA---TTTCATAT  4896
2-AAJO01000077    5758 AAGCTGAGAGTGC-TTTATTCTTTAGAAAAGTATCTGAAGAAAGCGAT----TTTGATGT  5812
3-AL766849        4738 -----GAAAATAC-TTAACT--TTTG--TTG-ATTCAGA-----TGATAAAGTTTCCT--  4779
4-AF355776        5410 -----GGCGGTATATTTCTTTTATTG------ATTCAGA-----CGATTAT-ATTGATA-  5451
5-AE009948        6335 -----GCGATTATATTTATTTACTTG------ATTCGGA-----TGATTA---TCTATA-  6374
6-HF952106        4712 -AGTTGGAAAT---TATATT-TTTAGAATGG-ATGCGGA-----CGATA---TTTCATAT  4757
7-AY376403        5412 -----GAGATTATATTTATTTACTTG------ACTCTGA-----TGATTA---TTTATA-  5451
8-AY375363        4539 ---------------TAACTGATCAA------ATTTTGA---AGCGATACAAGTCTTTCC  4574
9-CGBY01000002    5451 -----GCGATTATATTTATTTACTTG------ATTCGGA-----TGATTA---TCTATA-  5490
                                          *            *     *      ***     *   *

1a-CP000114       4891 CTGATTACAT-------AGCGAA--TTTGTATAATGCTATTCAAAAACATGA-----TTC  4936
1b-FO393392       4897 CCAAGTAGATTTGAT-AAGCAAA--TTCGTTTTATG--------GAGGAAAA-----TTC  4940
2-AAJO01000077    5813 TTATATGCATCTATT-AAATAAACTTTAAATTAAGGA-----GAAAATATTGAAACTTTT  5866
3-AL766849        4780 CTGATTACAT-------AGCGAA--TTTGTATAATGCTATTCAAAAACATGA-----TTC  4825
4-AF355776        5452 AAAATTATATTAGAA-AAATGTATTATTGTTTAAGTA--------ATCATAAAG-----T  5497
5-AE009948        6375 TAAAGAAGATGCAATCGAGAGAA---TGGTTGAATTT--------AGCGAGAAA------  6417
6-HF952106        4758 CCAGAAAGATTTGAA-AAGCAAA--TTAACTTTATGG-----AAAAGTATC---------  4800
7-AY376403        5452 TAAAGAAGATGCAATTGAGGAAA---TGGTTGGATGT--------AGTAAGGGA------  5494
8-AY375363        4575 CTAAA---ATTTATC-AGGCAG----CTATTTCATAT--------AAAATT--------T  4610
9-CGBY01000002    5491 TAAAGAAGATGCGTTCGAGAGAA---TGGTTGAATTT--------AGCGAGAAA------  5533
                               **                       *           *

1a-CP000114       4937 GTCTATAGCTA------TCGGTGGCTATT-TAGA-------ATTTTATGAAAG--ACATA  4980
1b-FO393392       4941 AT-TGGATTTC------TCAGCAACTCTAATAGA-------AT----TGATAG--ACCAA  4980
2-AAJO01000077    5867 ATTTATTGTTCCATATTTTGGAAAATTTAATAGTTATTTTCAGTTATTTTTAA--ACTCA  5924
3-AL766849        4826 GTCTATAGCTA------TCGGTGGCTATT-TAGA-------ATTTTATGAAAG--ACATA  4869
4-AF355776        5498 AGATATGGTTA------TCTGCAATTATTTGAGT--------GTTTATAATAATGACTTG  5543
5-AE009948        6418 ---TATAATTC--------AGAAATTGTTTTAGG-------GTGTTATGTTGA--A-AAA  6456
6-HF952106        4801 ATTTAGACTTC------TCAGCAACTTTAATAGA-------AGTTATGGATGA--AGATG  4845
```

```
7-AY376403       5495 ---TATGATTC--------AGATATTGTTTTAGC-------ATGTTATGTTGA--A-AGA  5533
8-AY375363       4611 CTGCGTGTTTT---------AGAGTTATCTTGAA-----------TGCTCCAA--ACATA  4648
9-CGBY01000002   5534 ---TATAATTC--------AGAAATTGTTTTAGG-------GTGTTATGTTGA--A-AAA  5572
                                *                *  *                         *

1a-CP000114      4981 ATAGC----ATAAGAAATTATGAATAT----TTAGACAA-----AGTGATACCAGTTGAA  5027
1b-FO393392      4981 AAAG---------GAAATTT--AGTAT----ATAAACAA-----CGAGAAAGTAATAAAA  5020
2-AAJO01000077   5925 ATAGCCA-CATCAGATCTTTGTGATTT----ATTGATTGTTACCGATGATAAAAGTAAAT  5979
3-AL766849       4870 ATAGC----ATAAGAAATTATGAATAT----TTAGACAA-----AGTGATACCAGTTGAA  4916
4-AF355776       5544 AAACCGATCGTTAGTCGTTTACAAGATTGTATTATATATTCTAGGGTGGAATTATTTAGT  5603
5-AE009948       6457 AGAGA----ACAACATATTATAAATATCGTTTTAGAAGATGA--AATGATAGAAACTATT  6510
6-HF952106       4846 A-AGAAA-CATTA-------------T----ATAAACAG-----AAAAATATAAATAAAA  4881
7-AY376403       5534 ATAGG----ACAACGAATTATAAATATTGTTTTAGAGAATGA--AACAATAGAAACAATT  5587
8-AY375363       4649 GAAAA-------ATATACTCCCATTGT------AGCTGA------ATGTAAAAAATATGT  4689
9-CGBY01000002   5573 AGAGA----ACAACATATTATAAATATCGTCTTAGAAGATGA--AACGATAGAAACTATG  5626
                        *                       *                       *  *

1a-CP000114      5028 GAAGCACTACTAAACATGT-----------------------------------------  5046
1b-FO393392      5021 TATACTTAACTAATGATAT-----------------------------------------  5039
2-AAJO01000077   5980 ACGATTACCCTCAGAATGTTACTATTCGCTATACTAGTTTTGAAGAGTTCCGAAATGAAG  6039
3-AL766849       4917 GAAGCACTACTAAACATGT-----------------------------------------  4935
4-AF355776       5604 AAT-TTATATGGTAAATAT-----------------------------------------  5621
5-AE009948       6511 AGTCCAGTACAAGCAATAC-----------------------------------------  6529
6-HF952106       4882 ATTATT--TTTCAGAAGATATTTAT-----------------------------------  4904
7-AY376403       5588 AGTCCGCTACAAGCAATAC-----------------------------------------  5606
8-AY375363       4690 ATATTATCACTGGAAAGAT-----------------------------------------  4708
9-CGBY01000002   5627 AGTCCAGTACAAGCAATAC-----------------------------------------  5645
                                     *

1a-CP000114      5047 -----------ATG----ACATTA----------AAAC--------------TTATGGTT  5067
1b-FO393392      5040 -----------ACGGAAGATGTTATTGAA----TAGAT--------------CTATACTT  5070
2-AAJO01000077   6040 TCCAAAGAAAAATG---GATTTTAAAATTACTTTAGATACTCCATACAAACTCTGTGATT  6096
3-AL766849       4936 -----------ATG----ACATTA----------AAAC--------------TTATGGTT  4956
4-AF355776       5622 ----------AAGG------------------------------------------ATCCTT  5631
5-AE009948       6530 --------AAAATA------TTTA---------TAATTA-------------TGATGCCT  6553
6-HF952106       4905 --------AGAATG---CTCTCTAA--------TAGATG--------------TGTCCTT  4931
7-AY376403       5607 --------GAAATA------TTTA---------TAATTA-------------TGATGACT  5630
8-AY375363       4709 ------------------------------------------------------------  4708
9-CGBY01000002   5646 --------AAAATA------TTTA---------TAATTA-------------CGATTCCT  5669

1a-CP000114      5068 --CAATT-------------TTTATTACTG------CATGGGGAA--AATTATTCCA---  5101
1b-FO393392      5071 GCCCACC-------------CAACGTGGTG------CGTAAAAAGAAAGTTTTCGA---  5108
2-AAJO01000077   6097 TTAAACCAACATACGGTTATCTATTTGAAGATGAATTGTTAAATTATCAATATTGGGGTT  6156
```

```
3-AL766849       4957 --CAATT-------------TTTATTACTG------CATGGGGAA--AATTATTCCA---  4990
4-AF355776       5632 TTACAAC--------------AGCCTGGGG-----------------AAGTTTAA-----  5655
5-AE009948       6554 ATAGAGC------------TATTTTTACTG------TAGCACATAATAAATTATA-----  6590
6-HF952106       4932 TCACATC-------------CCACTTGGTG------TGTTAAAAGAGCAGTATTT-----  4967
7-AY376403       5631 ATAAAGC------------TATTTTTACTG------TAGCACATAATAAATTATA-----  5667
8-AY375363       4709 ----ATT-------------TTATTTAATA----------------AAAAAACTCGAA--  4733
9-CGBY01000002   5670 ATAGAGC------------TATTTTTACTG------TAGCACATAATAAATTATA-----  5706
                          *                    *                      *

1a-CP000114      5102 --TAAATCT---AT---AT--TCAATGATTTAGAA------------TTTGCATTAAATA  5139
1b-FO393392      5109 --TAAGTTA---ATGGGAT--ATAGAGATTTAGTA------------CCTGT------TG  5143
2-AAJO01000077   6157 ATTGTGACTTAGATATTATCTTTGGTGATTTAGATGGATTTTTACATCCCATACTAAATA  6216
3-AL766849       4991 --TAAATCT---AT---AT--TCAATGATTTAGAA------------TTTGCATTAAATA  5028
4-AF355776       5656 --TAAGAAC-AGAT---ATCGCTAAGAAAGTAACA------------TTTCCAATTGGTA  5697
5-AE009948       6591 --TAAGAGA-GAATT--ATTTTCTACGCTGTGCTA-----------TCCAGTA---GGGA  6631
6-HF952106       4968 -TTAAGGCT-AGATG-----------GATATAGAAACATTGTT----CCTGCA------G  5004
7-AY376403       5668 --TAAGAGA-GAATT--ATTTTCTACAATGTGTTA-----------CCCAGTA---GGAA  5708
8-AY375363       4734 --TAAAAAC----------------TAAATTAGCA-------------------------  4750
9-CGBY01000002   5707 --TAAGAGA-GAATT--ATTTTCTACGCTGTGCTA-----------TCCAGTA---GGGA  5747
                        *                           *

1a-CP000114      5140 AGTATCATGAGGATGAATTCTTTAACTATAA-AGCATAC-----TTAAAAGCTAATTCTA  5193
1b-FO393392      5144 AAGATTATGA----------TTTTGCAATAA-GAGGAGC-----TCT--GGCTGATTTCA  5185
2-AAJO01000077   6217 AAGGCTTTGATAA--AATTTTTGAGCTAGGTCATTGTACGATTATTAAGAATGAATCACG  6274
3-AL766849       5029 AGTATCATGAGGATGAATTCTTTAACTATAA-AGCATAC-----TTAAAAGCTAATTCTA  5082
4-AF355776       5698 AACTACACGAGGATGAATTTACAACTTATAA-ATATTAC-----TTATATTCAGATAAAA  5751
5-AE009948       6632 AGTTACACGAAGATGAATTTTTAACCTATAA-ATTATAT-----TTGAAAGCCAAAAATA  6685
6-HF952106       5005 AGGATTATGAT-------TTTTTACTAAGGTTAGCGT-----TGTCAGGAATTAAATGTG  5052
7-AY376403       5709 AACTACACGAAGATGAATTTCTAACTTATAA-ATTATAT-----TTGAAAGCAAAAAATA  5762
8-AY375363       4751 --------------------TTAATTTCAATTGTTTTAT-----TTA--AACCGTTTATA  4783
9-CGBY01000002   5748 AATTACACGAAGATGAATTTTTAACCTATAA-ATTATAT-----TTGAAAGCCAAAAATA  5801
                                                                *

1a-CP000114      5194 TAA----CATACATAGACAAGCCTCTCTATCATTATCG--TATACGAGTA----------  5237
1b-FO393392      5186 AAATCGGCTTACTCAATAAAGTACTTTTA-CAGTATAGATTAAACGAGAA----------  5234
2-AAJO01000077   6275 AAT------AAATCGAATGTTCCTTTCTACAGTTAATG---GGGTGAAAATAGGACAAAA  6325
3-AL766849       5083 TAA----CATACATAGACAAGCCTCTCTATCATTATCG--TATACGAGTA----------  5126
4-AF355776       5752 TAG------------------CCTTTGTACCAGAACCAC-TATATTTTTA----------  5782
5-AE009948       6686 TAA-------------------TTTTCTTTAGATATAATACTTATGCATA----------  6716
6-HF952106       5053 GTT------TAATCG----------------------------GTGATA-----------  5067
7-AY376403       5763 TAA-------------------TTTTCTTTCGATATAACACTTATGTATA----------  5793
8-AY375363       4784 AAA-------------------------------------TTTATTTATA----------  4796
9-CGBY01000002   5802 TAA-------------------TTTTCTTTAGATATAATACTTATGCATA----------  5832
```

```
1a-CP000114      5238 ------------------------------GGTAGTATCATGAAT-AATAG--TGATAA-  5263
1b-FO393392      5235 ------------------------------TGGAATATCACAAACCAATAA--GTTTAAG  5262
2-AAJO01000077   6326 AGCTCTAAGTTCTTCCAAAATCGAAGTTTTGATGAGGCATATGTTAATAG--TATTAA-  6382
3-AL766849       5127 ------------------------------GGTAGTATCATGAAT-AATAG--TGATAA-  5152
4-AF355776       5783 --------------------------------TAGAAGGAGAGAG-GCTAGTATAATGAG  5809
5-AE009948       6717 --------------------------------TCGTATTAGAGAA-AATAGCATAATGAC  6743
6-HF952106       5068 ---------------------------TATTGCTTAGATATAGG--AGTAA--TGCTAA-  5095
7-AY376403       5794 --------------------------------TCGTATTAGAGAA-GACAGTATAATGAC  5820
8-AY375363       4797 --------------------------------------CACACGTCGATAG-ATGGAAAT  4817
9-CGBY01000002   5833 --------------------------------TCGTATTAGAAAA-AATAGCATAATGAC  5859
                                                             *         *        *

1a-CP000114      5264 TGTTATAATTGCTAGAAAGAAACTTGATGTTTTATCA-GCATTAGACGAGCGAATA-AAA  5321
1b-FO393392      5263 CAATATATTTACTCA----------GCTATTTTACAA-GATTTTTATAAA-GAA-A-AAT  5308
2-AAJO01000077   6383 TAATATATTTATTGAAAATAATTGTAATAACTTTTTGTACAGTTTAGGAGCAGACATAGA  6442
3-AL766849       5153 TGTTATAATTGCTAGAAAGAAACTTGATGTTTTATCA-GCATTAGACGAGCGAATA-AAA  5210
4-AF355776       5810 TTCTTCTTACAGTAAAAGAAATTTAGATTCTTTAGAA-GCTTATGAAGAACGGATTAAAA  5868
5-AE009948       6744 TGGTTCCTACAATATTAAAAGGCTACATGCTGTAGAA-GCACTTAAGGAAAGAATTTA--  6800
6-HF952106       5096 TGGTAT-TTCAC--AAACTAATCAATATAA--------ACAGTATA----TATACGCAGC  5140
7-AY376403       5821 TGGTTCTTACAATGTTAAAAGATTACATGCTATAGAA-GCACTCCAGGAAAGAATTCA--  5877
8-AY375363       4818 AGGTTTATAAAATAAAAGAAAG---GTTGGTTTATAAAGCGTTT---------------A  4859
9-CGBY01000002   5860 TGGTTCTTACAATATTAAAAGGCTACATGCTGTAGAA-GCACTTAAGGAAAGAATTTA--  5916
                         *                       *

1a-CP000114      5322 CTTATAACAAGTTTACGTAAGTATTCCGTTTTTTTACAAAAA------ACAGAAATATTT  5375
1b-FO393392      5309 CTTATATTGATATCACAAAAAT-TACTAATTACTTTCAAGAGTATGTGATAAAGAAACGC  5367
2-AAJO01000077   6443 CATTTGGAAGAATAATTTTTATATT---ACTTCTTTCAAGAA-------TAAAAAAGATT  6492
3-AL766849       5211 CTTATAACAAGTTTACGTAAGTATTCTGTTTTTTTACAAAAA------ACAGAAATATTT  5264
4-AF355776       5869 TATTGGAAAAAAATA-----ATATATCTATATC----AGAAACTGT--GTACATATACCT  5917
5-AE009948       6801 TTTATTAGAAAAGTATCCAGAC-TTAGTATTTC-----AAAG-------TGAGAGAGCTT  6847
6-HF952106       5141 TAT-----------ACTTCAGGATT---GGTTC----AAAAA-------TGGACAAA---  5172
7-AY376403       5878 TTTATTAAAAGAGTACCCAGAG-TTAGTATTTC-----AAAG-------TGAAAAAGCTT  5924
8-AY375363       4860 TTTCTGA-------------GTATT----CTAT-----AAAG-------TAGATGAGGT-  4889
9-CGBY01000002   5917 TTTATTAGAAAAGTATCCAGAC-TTAGTATTTC-----AAAG-------TGAGAGAGCTT  5963
                        *                    *                *

1a-CP000114      5376 T----ATGTCAATCA--------ATATTTTA----GGACTAAAAAATTT---TTAA----  5412
1b-FO393392      5368 T----ATACTCAGCA--------AGAGCTC-------TCTAAATATTTTGAGCTAA----  5404
2-AAJO01000077   6493 TTGTAGTATCAGACGCTCCCAGCATATTTTACTATGAACAAGGACACTTATATGAGTTTC  6552
3-AL766849       5265 T----ATGTCAATCA--------ATATTTTA----GGACTAAAAAATTT---TTAA----  5301
4-AF355776       5918 T----AGTTTAATTCT-------ATATCATA------TATACAGAATTCGT---------  5951
5-AE009948       6848 T---------AATTA--------AAACTATG-------GAAGTAAATTT-----------  6872
6-HF952106       5173 ------TGTTAAATGT----AGACTATATTCCTATTTATCTTGAAAGCTATATAAAT---  5219
```

```
7-AY376403        5925 T---------AATTA--------AAACTATG-------GAAGTAAACTT-----------  5949
8-AY375363        4890 ----------AACTA--------GGAATATG-------------AATTT-----------  4907
9-CGBY01000002    5964 T---------AATTA--------AAACTATG-------GAAGTAAATTT-----------  5988
                                                * 

1a-CP000114       5413 ---AACA----ACAATCTGTAATGTTTAA-AGAAGATAACTATATCGATGCTT-------  5457
1b-FO393392       5405 ---AAT------CTACCCCTAGTATTACT-ATTAGAAAACTATATATTTGTTT-------  5447
2-AAJO01000077    6553 ACCAATA----TAAGTCTGTAGTAAGAAGGAGTGAGTATTTATATATTCATCTTCAACAA  6608
3-AL766849        5302 ---AACA----ACAATCTGTAATATTTAA-AGAAGATAATTATATCGATGCTT-------  5346
4-AF355776        5952 --AAATATCCTGAGGTATGCAATAAACAA-------GAATTATT----------------  5986
5-AE009948        6873 ---GATA----GAGTTATATAAAAATAA--------TTTTTATA----------------  6901
6-HF952106        5220 AGCAATA-----AATTTTGT--CAAAAA--------CACTTA------------------  5246
7-AY376403        5950 ---GACA----GAGTTATACAAAAATGA--------TTTTTATA----------------  5978
8-AY375363        4908 ---AATA-------ATGTGTAGAACTCCC-------------------------------  4926
9-CGBY01000002    5989 ---GATA----GAGTTATATAAAAATAA--------TTTTTATA----------------  6017
                           *

1a-CP000114       5458 ATAGA-AT--------GTATGGACGGTTA--CTTAGAAAAGTGAAGTTAGTTGATAAATT  5506
1b-FO393392       5448 ATATTTAT--------ACTTTAAGTCTCC--CTTGGTTA---GAAGGTTATTAATAAAT-  5493
2-AAJO01000077    6609 AGAAAAATGAAAGTTGATTTAGATGCTAG--GTCTACAACTTATCTGATATTACCAAAT-  6665
3-AL766849        5347 ATAGA-AT--------GTATGGACGGTTA--CTTAGAAAAGTGAAGTTAGTTGATAAATT  5395
4-AF355776        5987 GGATAAGT-----------TTAATAACTATTATTTAAAGTATAAAAA-TATTTTAGGAT-  6033
5-AE009948        6902 --AGGAAT---------TTCATACTCTGAAAACTGAATATAAAAAAACTATTTTTGATT-  6949
6-HF952106        5247 -AAAAAAT--------ACCTAGACTTTAA--ATTTAGTAGTAGTCT--TATTGCTAAAA-  5292
7-AY376403        5979 --AGGAAT---------TTTATACTCTAAGGAATGAATATCAGAGAACTATTCTTGATT-  6026
8-AY375363        4927 ----------------CTTCAGACTATAATTGCTGAACG------GATTATTGAGATGT-  4963
9-CGBY01000002    6018 --AGGAAT---------TTCATACTCTGAAAACTGAATATAAAAAAACTATTTTTGATT-  6065
                                             *                           **

1a-CP000114       5507 AAAACTTATAAAAAA-TAGGTTTTTCTAGAAATGGCTGTTTAATAGTTG--TAATGTGAG  5563
1b-FO393392       5494 GATATTAATA-------------TTTTAGTACTGA------AATTGTTT--GGAGGAGAG  5532
2-AAJO01000077    6666 -AAGTTCGTAGAATA-TA-GAATAGCTACAAATTGCTTGTTAACACTTAAAGAATTTAAA  6722
3-AL766849        5396 AAAACTTATAAAAAA-TAGGTTTTTCTAGAAATGGCTGTTTAATAGTTG--TAATGTGAG  5452
4-AF355776        6034 --------TAAAAAA------TAGACTAAA-----------AATTATTAC----ATTAAA  6064
5-AE009948        6950 -----TTATAAAAAAACAACGTATGCTACTAAAAA----TAAAGTATTA-----TTTAAA  6995
6-HF952106        5293 -AAGTCTTTTGGGGA----GGATTTTTAT------TTTTCAAATATCCT-----TTTGCA  5336
7-AY376403        6027 -----TTATAGAAAAACAAGGTGTGCTACTAAAAG----TAAAATATTC-----TTTAAA  6072
8-AY375363        4964 ----TTCCCAATGAA------TCATTTGAAG--------TTATTTGTTAT----TTTGAG  5001
9-CGBY01000002    6066 -----TTATAAAAAAACAACGTATGCTACTAAAAA----TAAAGTATTA-----TTTAAA  6111
                                                 *              *

1a-CP000114       5564 TGACCACGAATTGTTGG-----ATAACTATCAAAA-----------AGACTAATTTAGGG  5607
1b-FO393392       5533 AAA---------------------------CAAAG-----------TGACTAAT-----A  5549
2-AAJO01000077    6723 CGGTTTATTAGTTTTGGAAAATATAGCTTTCAAAAATACCGCCTATATACTAAATTACAA  6782
```

```
3-AL766849       5453 TGACCACGAATTGTTGG-----ATAACTATCAAAA-----------AGACTAATTTAGGG  5496
4-AF355776       6065 GAATTTAGGCATACC-----------TTATCAAAGATA-----TAAACATAAACTTTGAA  6108
5-AE009948       6996 GTATTGTGTAGTGC------------ATTTTAAAA------------TATTAAATTGTAA  7031
6-HF952106       5337 CG-TCGATTAAT------AATTCAAGATTTTAA----------TATTTACAAAATAAAAA  5379
7-AY376403       6073 ATATTGCATATTGT------------ATTTTAAAA------------TATTAAATTATAA  6108
8-AY375363       5002 TCAAGAAATAAAAAA---------------CAAGC-----------ATATTATTCAACAA  5035
9-CGBY01000002   6112 GTATTGTGTAGTGC------------ATTTTAAAA------------TATTAAATTGTAA  6147
                                                     **               *  *

1a-CP000114      5608 ----GAAAAATAAAATGACTAA-----------------------------TTTTGTTTG  5634
1b-FO393392      5550 ----GAAAAAT----------------------------------------TTATGTATG  5565
2-AAJO01000077   6783 AAGCAAAAAATAAAGAAGTTATTATGAGATTTTAATGAAAATGAAGAATATTTATATATG  6842
3-AL766849       5497 ----GAAAAATAAAATGACTAA-----------------------------TTTTGTTTG  5523
4-AF355776       6109 TACTGAAGAAAGTTAAGAATGAA----------------------GAAAATATATATTTG  6146
5-AE009948       7032 AAGAAAGAAAAAGATAAATAAAT----------------------GAATAATTTTGTGTG  7069
6-HF952106       5380 TATTGAACGGTAAGAGGACAAC----AGAT---------GATGAGTAATATATATGTGTG  5426
7-AY376403       6109 AAGAAAGAAAAATATGAATAAAT----------------------GAATTATTTTGTGTG  6146
8-AY375363       5036 GATTAAAAAAAAAAA---------------------------------------GTGAG  5055
9-CGBY01000002   6148 ACGAAAGAAAAAGATAAATAAAT----------------------GAATAATTTTGTGTG  6185
                           *                                                  *  *

1a-CP000114      5635 TCATACTTTATACCATCTACTGATTACTATA-ATAAAGTT----AAAAGATAAAGAAAAT  5689
1b-FO393392      5566 TCATACTCTTTATCATTTATTGATTTGTTTATATAAAG------AAGAGATATATTCAAA  5619
2-AAJO01000077   6843 TTACACTTTATATCACCTATTGATATCTTTATGTAAG--------AGAGAT---TTATAT  6891
3-AL766849       5524 TCATACTTTATACCATCTACTGATTACTATA-ATAAAGTT----AAAAGATAAAGAAAAT  5578
4-AF355776       6147 CCATACAGTATATCATCTCCTTATTACAATTATCAAAATATCTATAATGAGGGAACAGAG  6206
5-AE009948       7070 TCATACTTTATACCATTTACTGATTACTATA-ATAAAGTT----AAAAGATAAAGAAAAT  7124
6-HF952106       5427 TCATACTTTATACCATTTATTAATTTGTTTATATAA---------AGAAACAGATTACAC  5477
7-AY376403       6147 TCATACTTTATATCATCTACTGATTACTATA-ATAAAGCT----AAAAGATAAAGAAAAC  6201
8-AY375363       5056 CTATA-----TACCGA--AATAGTATTTAAAAATAGGGCTC---AGGAGATAT-------  5098
9-CGBY01000002   6186 TCATACTTTATACCATCTACTGATTACTATA-ATAAAGTT----AAAAGATAAAGAAAAT  6240
                        * *     ** *      *  *          *              *

1a-CP000114      5690 ACTAGAA-TATTTATTTGTGACACTATCAC------GGATTATGA-AACTTGGGTAAAGA  5741
1b-FO393392      5620 TTTAGAAATCATTCTCTCTTCTTCTATTCC------TGATGTTGA-TAATTTAGAGAAAA  5672
2-AAJO01000077   6892 GGAAAA-ATAATTATCTTAACTACTCGAAT---AAATAATTATGC-AATATATAAAAGAA  6946
3-AL766849       5579 ACTAGAA-TATTTATTTGTGACACTATCAC------GGATTATGA-AACTTGGGTAAAGA  5630
4-AF355776       6207 GAGATCAATAATTATATTGGCTAATAAGAT---AGTTAATATTGATGATTTATGTAAACG  6263
5-AE009948       7125 ACTAGAA-TATTTATTTGTGACACTATCAC------GAATTATGA-AACTTGGGTAAAAA  7176
6-HF952106       5478 GAATACTGTAATTGTATTATCTTCTTCCATTTTGAATGCTAGTGA-ATTACAGAAAA---  5533
7-AY376403       6202 ACTAGAA-TATTTATTTGTGATACTATCCC------TGATTATGG-AGCTTGGATAAAAA  6253
8-AY375363       5099 --------TAACTA--TAATATACTTTCGT--------TTTAT----ATTTCTGAGGAAA  5136
9-CGBY01000002   6241 ACTAGAA-TATTTATTTGTGACACTATCAC------GAATTATGA-AACTTGGGTAAAAA  6292
                              *   *   *       *              *  *
```

```
1a-CP000114       5742 TATTA-AATGATCAGGGAATAAGAACTGAATCATTTAAAGAGTTTGCTTATCGGGAGCAG  5800
1b-FO393392       5673 AATTA-AA--ATCAAA-AACAATAA-------ATATACATATTTTA-------GAAGAAT  5714
2-AAJO01000077    6947 GAATA-GAAAAGTTGTTTCCAGAAAT------ACCTGTATTGATTG-TAGATGATAGTGA  6998
3-AL766849        5631 TATTA-AATGATCAGGGAATAAGAACTGAATCATTTAAAGAGTTTGCTTATCGGGAGCAG  5689
4-AF355776        6264 TACCA-ATAAAGAAGGTA----------------TTCTATGTTTTA------------AA  6294
5-AE009948        7177 CATTA-AATGAACAGGGAATAAGGACGGAAGCAGTTAAAGAGTTTGCTTATCGGGAGCAG  7235
6-HF952106        5534 -AGTT-AAAAAGT-GTAGGGGCTAAC------GTTTATATTT---------TGACAGAGT  5575
7-AY376403        6254 TATTA-AATAATCAGGGAATAAAAACTGAATTATTTAAAGAGCTAGATTATCGGGAACAG  6312
8-AY375363        5137 TATTACGATAAA--------------------ATCTATATTG------------------  5158
9-CGBY01000002    6293 CATTA-AATGAACAGGGAATAAGGACGGAAGCATTTAAAGAGTTTGCTTATCGGGAGCAG  6351
                        *        *                        *  *

1a-CP000114       5801 TTAC-AAAATAAAAATATTGAAGAAGTGATGGAGCTTGTAGATTCAGACTTAAATCACTA  5859
1b-FO393392       5715 CTA--GTGGTGAAAGTGAAGAGTTATTATCAGTACTTAAAGATGCTGGTCTAAGTTATAG  5772
2-AAJO01000077    6999 TTACAGTAAGAAATAT-AAATTGTTTTATCCGAAGCGAATAAAACAAGAATTGTTTAGTA  7057
3-AL766849        5690 TTAC-AAAATAAAAATATTGAAGAAGTGATGGAGCTTGTAGATTCAGACTTAAATCACTA  5748
4-AF355776        6295 TTA----GATGAAACTTCGAGTACTCCAACTA---CTGTAGATACAATAACAATTGACTT  6347
5-AE009948        7236 TTAC-AAAGTAAAAATATTGAAGAAGTGATGGAGCTTGTAGATTCAGACTTAAATCACTA  7294
6-HF952106        5576 CAGC--TAAGGAAAATGAAGGAGTTATATTAAAACTTAAAAAAATTGGT-TTAAGGAACA  5632
7-AY376403        6313 TTAC-AAAATAAAAACGTTGAAGAAGTGATGAACCTTGTAGATTCATATTTAAAACACTA  6371
8-AY375363        5159 CTAACATAACAAATGTTTTG---------------TTTCATACAATATTATCAGCTTCTA  5203
9-CGBY01000002    6352 TTAC-AAAGTAAAAATATTGAAGAAGTGATGGAGCTTGTAGATTCAGACTTAAATCACTA  6410
                                  **                            *

1a-CP000114       5860 C---TTTGAGAGAGTAGATACACAAGTCTATCTATTTAATGATG--ATACTT--TAATAG  5912
1b-FO393392       5773 CAAGTTTGATAG--TAATTGT-----TTTATT--TTTAATGATGCAACGCCTATTGGGAG  5823
2-AAJO01000077    7058 TAA-CCAACGGCAATAAAATA-----CATATT--TACAATGACTATACGCA-ATTAGGAT  7108
3-AL766849        5749 C---TTTGAGAGAGTAGATACACAAGTCTATCTATTTAATGATG--ATACTT--TAATAG  5801
4-AF355776        6348 AA--TTATAGAAACTAATGGAACTA-TATATTTATTTAATGATG--ATACTAG--TGTAG  6400
5-AE009948        7295 C---TTTGAGAGAGTAGATACACAAGTCTATCTATTTAATGATG--ATACTT--TAATAG  7347
6-HF952106        5633 CAA-ACGGCAGGAGTAATTGT-----TTTATT--TTTAATGATGCAACGCCTATTGGGAG  5684
7-AY376403        6372 C---TTTGAGAGAGTAAATACACAAGTCTACCTATTTAATGATG--ATACCT--TAATGG  6424
8-AY375363        5204 CT--TTTAATAAAATT----------TTTACT--TTTGATGATGG---ACTTGCTAATAT  5246
9-CGBY01000002    6411 C---TTTGAGAGAGTAGATACACAAGTCTATCTATTTAATGATG--ATACTT--TAATAG  6463
                                     *             **    *   ****       *

1a-CP000114       5913 GTCGCTATATGGTGTATTTAGGTAAAAATTATCATCTAATTGAAGATGGTTATAACT---  5969
1b-FO393392       5824 GACACTA-ATAAAGCAT---GGTATTTATTATAATCTAATTGAAGATGGTTTAAATT---  5876
2-AAJO01000077    7109 ATTTTTTTCATAGGAAT---GCTATTCCCTACCATTTAATGGAAGATGGATATAATA---  7162
3-AL766849        5802 GTCGCTATATGGTGTATTTAGGTAAAAATTATCATCTAATTGAAGATGGTTATAACT---  5858
4-AF355776        6401 CTCGGCAAATTGTACGCGCTCAGAAGAATTATCATCTAATTGAAGATGGTTATAACT---  6457
5-AE009948        7348 GTCGCTATATGGTGTATTTGGGTAAAAATTATCATCTAATTGAAGATGGTTATAACT---  7404
6-HF952106        5685 GACTCTAATA-AAGTAT---GGTATTTATTATAATCTAATTGAAGATGGTTTAAATT---  5737
```

```
7-AY376403         6425 GGCGTTATATGGCATATTTAGGTAAAAACTATCATCTAATCGAAGATGGATATAACT---  6481
8-AY375363         5247 A--ATTAAATCTAGTTT----------CTTATATGCTAGTAGGACATCTCTAAAAGCTAA  5294
9-CGBY01000002     6464 GTCGCTATATGGTGTATTTGGGTAAAAATTATCATCTAATTGAAGATGGTTATAACT---  6520
                                                     **     ** * * * **   *  **

1a-CP000114        5970 GTTTTCA--AGCCAAGTTATTCTTGGGTGGTT--CTGTCGTAA--AACGAGTAATTAAAA  6023
1b-FO393392        5877 GCTTT----ACTTACTCTATATTTAGTCAAAA--ACTTTGGAAGTATTATGTAAA-AAAA  5929
2-AAJO01000077     7163 GTTTAAAACATCCAACGAGTATCGCTCCAGATGGTCTTTGTGA---CAAGATAA---AAG  7216
3-AL766849         5859 GTTTTCA--AGCCAAGTTATTCTTGGGTGGTT--CTGTCGTAA--AACGAGTAATTAAAA  5912
4-AF355776         6458 GTTTTCA--AGCCAAGTTATTCTTGGGTGGTT--CTGTCGTAA--AACGAGTAATTAAAA  6511
5-AE009948         7405 GTTTTCA--AGCCAAGTTATTCTTGGGTGGTT--CTGTCGTAA--AACGAGTAATTAAAA  7458
6-HF952106         5738 GCTTTACTTACTCTA----TATTTAGGCAAA--GACTTTGGAAGTACTATGTAA---AAA  5788
7-AY376403         6482 GTTTTCA--AGCTAAGTTATTCTTTGGGGGTT--CCGTTGTAA--AACGAATAATTAAAA  6535
8-AY375363         5295 GTTTT------TTAAATT-TGTTTTTTGATTAAGTATGATGCGCAAAGGATAA--AAAA  5345
9-CGBY01000002     6521 GTTTTCA--AGCCAAGTTATTCTTGGGTGGTT--CTGTCGTAA--AACGAGTAATTAAAA  6574
                        * **               *                 *             ***   **

1a-CP000114        6024 CTTA-CTTATTTAAAAAGTATGTTCCGTATGGTTTTTCAAAATATTGTCTCTCAATTGAG  6082
1b-FO393392        5930 TATA-TTC-TTCACAGAAT-TCAGCCACATGGATTTTCCCGATATTGTTTAGGGATTGAA  5986
2-AAJO01000077     7217 GGAA-GATAACGAATACACCCCGATATCATGGCTTCAGTAAGTATTGTAAGACGATAGAA  7275
3-AL766849         5913 CTTA-CTTATTTAAAAAGTATGTTCCGTATGGTTTTTCAAAATATTGTCTCTCAATTGAG  5971
4-AF355776         6512 CTTA-CTTATTTAAAAAGTATGTTCCGTATGGTTTTTCAAAATATTGTCTCTCAATTGAG  6570
5-AE009948         7459 CTTA-CTTATTTAAAAAGTATGTTCCGTATGGTTTTTCAAAATATTGTCTCTCAATTGAG  7517
6-HF952106         5789 AATA-TATTCTTCGCAAAGTTCAGCCATATGGATTTTCACGATACTGTTTAGGGATTGAA  5847
7-AY376403         6536 CCTA-CTTTTTGAAAAAATATGTTCCATATGGTTTTTCAAAATATTGTCTTTCAATTGAG  6594
8-AY375363         5346 TGAGTCATCTTTACATTATACCTTATATAAAAACAATGACAATATTATAAAAAACACAAA  5405
9-CGBY01000002     6575 CTTA-CTTATTTAAAAAGTATGTTCCGTATGGTTTTTCAAAATATTGTCTCTCAATTGAG  6633
                                                    *             ** * *          *

1a-CP000114        6083 GTTAATTC--TTTAGTGGGGTTACCTCATGACATAAGGA-----GTAAGAAATATAAAGA  6135
1b-FO393392        5987 GTTAATTC--ATTAGTTAATTTGCCAAAGGATCCGCGTT-----ATAAAAAATTTATTGA  6039
2-AAJO01000077     7276 GTAAGTAG--TTTGAAAGATTTACCAACAGATGAGAGAA-----TGTCTAAGTTTATTGA  7328
3-AL766849         5972 GTTAATTC--TTTAGTGGGGTTACCTCATGACATAAGGA-----GTAAGAAATATAAAGA  6024
4-AF355776         6571 GTTAATTC--TTTAGTGGGGTTACCTCATGACATAAGGA-----GTAAGAAATATAAAGA  6623
5-AE009948         7518 GTTAATTC--TTTAGTGGGGCTACCTCATGACATAAGGA-----GTAAGAAATATAAAGA  7570
6-HF952106         5848 GTTAATTC--ATTAGTTGGTTTGCCAAAAGATCCACGTT-----ATAAAAAATTTATTGA  5900
7-AY376403         6595 GTAAATTC--ATTAGAGGGCTTACCTCACGACAGGAGAA-----GTAAAAAATATAAAGA  6647
8-AY375363         5406 GTTTATTTCTATTTTAAGAAATATTACTAAACATAAGGATGTTAGTAATAATTCTATATC  5465
9-CGBY01000002     6634 GTTAATTC--TTTAGTGGGACTACCTCATGACATAAGGA-----GTAAGAAATATAAAGA  6686
                        **   *     **        *        *     *            ** * **

1a-CP000114        6136 ACTACCGAGAAAAAAATTATTTGATAGTCTTAACAAAGAACAAAAATCACTG-ATTTTCA  6194
1b-FO393392        6040 AGTTCCTAGAAGAACACTTTTTGATAATTCAAATTCATATCAAAATGATATC-GTGATAG  6098
2-AAJO01000077     7329 GAAGCCCAAGGATGGATTATTTTCTGATTTAG-ATAATAAAACTAAGGAATCTATTTTAA  7387
```

```
3-AL766849       6025 ACTACCGAGAAAAAAATTATTTGATAGTCTTAACAAAGAACAAAAATCACTG-ATTTTCA  6083
4-AF355776       6624 ACTACCGAGAAAAAAATTATTTGATAGTCTTAACAAAGAACAAAAATCACTG-ATTTTCA  6682
5-AE009948       7571 ACTACCGAGAAAAAAATTATTTGATAGTCTTAACAAAGAGCAAAAATCACTG-ATTTTCA  7629
6-HF952106       5901 AGTCCCTAGGAAGGAACTTTTTGATAATGCAACAGAATATCAAAAAGAAATG-GCAATAA  5959
7-AY376403       6648 ACTACCGAGAAAAGAATTATTTGATAGTCTTACCCAAGAACAAAAGTCTCTG-ATTTTCA  6706
8-AY375363       5466 AATTTGTATAGGTCAACCACTATATAGCGATGATT--TAATGAATATACATT-ATTTCAA  5522
9-CGBY01000002   6687 ACTACCGAGAAAAAAATTATTTGATAGTCTTAACAAAGAGCAAAAATCACTG-ATTTTCA  6745
                             *       *    *   *            *           *

1a-CP000114      6195 AAATATTTAAAACAAAACCATTAACTAT------AACTCCAAAGTCAGTATTATTGTTGA  6248
1b-FO393392      6099 ATATTTTTGGAGTTAACCGAGTGATTGTT-----AAATCTCCAG-CAGTTTTAATATTGA  6152
2-AAJO01000077   7388 AGATTTTTGACATAGCCTCAATAGATATTGAAATAGCTTCTCCATCTGCTCTAATATTAA  7447
3-AL766849       6084 AAATATTTAAAACAAAACCATTAACTAT------AACTCCAAAGTCAGTATTATTGTTGA  6137
4-AF355776       6683 AAATATTTAAAACAAAACCATTAACTAT------AACTCCAAAGTCAGTATTATTGTTGA  6736
5-AE009948       7630 AAATATTTAAAACAAAACCATTAACTAT------AACTCCAAAGTCAGTATTATTGTTGA  7683
6-HF952106       5960 ATCTTTTTGG---AGC---AGTAAAAGTTAGTATTAATTCACCTTCAGTACTGATATTAA  6013
7-AY376403       6707 AACTTTTTAAGATGAAACCGATAACTAT------AGCTCCAAAGTCAGTATTGTTATTAA  6760
8-AY375363       5523 TAATATT---------ATTCATAAATATG-----------ATATTGAGTATTATT--TCC  5560
9-CGBY01000002   6746 AAATATTTAAAACAAAACCATTAACTAT------AACTCCAAAGTCAGTATTATTGTTGA  6799
                         * **              *     *                  *   *  *  *

1a-CP000114      6249 CACAGCCACTTGCACAAGATAAATGGTATAAAACACCTA-CAGAGAGGTTTCAAAGTATT  6307
1b-FO393392      6153 CACAACCATTAGCGTATGATAATTGGTATAAAACACCGA-CAGAGAGGTTTCAAAGTATT  6211
2-AAJO01000077   7448 CTCAACCTTTA------GAGGAACATTTTAAAAGTCA--------AGA---GGAAATATA  7490
3-AL766849       6138 CACAGCCACTTGCACAAGATAAATGGTATAAAACACCTA-CAGAGAGGTTTCAAAGTATT  6196
4-AF355776       6737 CACAGCCACTTGCACAAGATAAATGTTATAAAACACCTA-CAGAGAGGTTTCAAAGTATT  6795
5-AE009948       7684 CACAGCCACTTGCACAAGATAAATGTTATAAAACACCTA-CAGAGAGGTTTCAAAGTATT  7742
6-HF952106       6014 CGCAGCCTTTATCTAAAGATAAAGAATTTATGAGTTATAACAATAAGAT--AGAAACGTC  6071
7-AY376403       6761 CGCAGCCACTTGCACAGGATAAATGGTATAAAACAGCTA-CAGAAAGGTTTCAAAGTATT  6819
8-AY375363       5561 CACATCCAC--------GGGAAACCTTT-----------------AGGATTGACAATAT-  5594
9-CGBY01000002   6800 CACAGCCACTTGCACAAGATAAATGTTATAAAACACCTA-CAGAGAGGTTTCAAAGTATT  6858
                      * ** **          *   *    *                 **       *   *

1a-CP000114      6308 C-AAGAGCAATACGATTATTTTGACGATATTGTCCAGGAATATAGAACGTTAGGGTACAA  6366
1b-FO393392      6212 C-AAGAGCAATACGATTATTTTGACGATATTGTCCAGGAATATAGAACATTAGGGTACAA  6270
2-AAJO01000077   7491 CCAA------------TTTTACAAAAGCATAGTGGAAGAATATATTGATAAAGGTTACCA  7538
3-AL766849       6197 C-AAGAGCAATACGATTATTTTGACGATATTGTCCAGGAATATAGAACGTTAGGGTACAA  6255
4-AF355776       6796 C-AAGAGCAATACGATTATTTTGACGATATTGTCCAGGAATATAGAACGTTAGGGTATAA  6854
5-AE009948       7743 C-AAGAGCAATACGATTATTTTGACGATATTGTCCAGGAATATAGAACGTTAGGGTACAA  7801
6-HF952106       6072 CGAAGAACAATTTAATTTTTATAAATCAATAGTCGACGAATATATAAATAAAGGATACAA  6131
7-AY376403       6820 C-AGGAGCAATACGATTATTTTGAGGGGATTGTCCACGACTATAGAGAACGAGGGTACAA  6878
8-AY375363       5595 ----------------TATTT-----ATATTGAGACACAATGTATTTTTGAAGAATATGT  5633
9-CGBY01000002   6859 C-AAGAGCAATACGATTATTTTGACGATATTGTCCAGGAATATAGAACGTTAGGGTACAA  6917
                                      * **        ** *     * * **       **  **
```

```
1a-CP000114       6367 TGTTTATTTAAAAGTTCATCCTAGAGATGTAGT-AGATTATTCCAAATTGCCGGTAGAGC  6425
1b-FO393392       6271 TGTTTATTTAAAAGTTCATCCTAGAGATATAGT-GGATTATTCCAAATTGCCGGTAGAGT  6329
2-AAJO01000077    7539 CATATATATTAAAGTTCACCCTAGAGATAACAT-TGACTACTCTAATCTCAATGCAACAG  7597
3-AL766849        6256 TGTTTATTTAAAAGTTCATCCTAGAGATGTAGT-AGATTATTCCAAATTGCCGGTAGAGC  6314
4-AF355776        6855 CGTTTATTTAAAAGTTCATCCTAGAGATGTAGT-AGATTATTCCAAATTGCGGGTAGAGC  6913
5-AE009948        7802 TGTTTATTTAAAAGTTCATCCTAGAGATGTAGT-AGATTATTCCAAATTGCCGGTAGAGC  7860
6-HF952106        6132 TGTTTATTTAAAAGTTCATCCTAGAGATGTAGT-AGATTATTCCAAATTGCCGGTAGAGC  6190
7-AY376403        6879 TATTTATTTAAAAGTTCACCCTAGAGATGCAGT-AGATTATTCCAAATTGCCAGTGGAGT  6937
8-AY375363        5634 TGTC--------AATTTACTTAAGGAATTTTCTGAGATTAATCTTTATACTTGTTTCAGT  5685
9-CGBY01000002    6918 TGTTTATTTAAAAGTTCATCCTAGAGATGTAGT-AGATTATTCCAAATTGCCGGTAGAGC  6976
                         *         * ** *    **  **    *  ** ** **

1a-CP000114       6426 TAT-TACCATCAAATGTTCCTATGGAAATT-ATAGAGTTGATGT-----CAACAG-GTC-  6476
1b-FO393392       6330 TAT-TACCATCAAATATCCCTATGGAAATT-ATAGAGTTGATGT-----TAATAG-GTC-  6380
2-AAJO01000077    7598 TAA-TACAAAGGAACATCCCTATAGAA-TT-AT----TTGATTTCC---TAGAGA-ATAT  7646
3-AL766849        6315 TAT-TACCATCAAATGTTCCTATGGAAATT-ATAGAGTTGATGT-----CAACAG-GTC-  6365
4-AF355776        6914 TAT-TACCATCAAATATCCCTATGGAAATT-ATAGAGTTGATGT-----TAACAG-GTC-  6964
5-AE009948        7861 TAT-TACCATCAAATATCCCTATGGAAATT-ATAGAGTTGATGT-----TAACAG-GAC-  7911
6-HF952106        6191 TAT-TACCATCAAATGTTCCTATGGAAATT-ATAGAGTTGATGT-----TAACAG-GTC-  6241
7-AY376403        6938 TAC-TACCATCAAATATCCCTATGGAAATT-ATAGAGTTGATGT-----TGACAG-GTC-  6988
8-AY375363        5686 TCCGCAGCGTTGAATGTC---ATAGATATTGATGGAATCAACGTTTTTATAATAAAGTC-  5741
9-CGBY01000002    6977 TAT-TACTATCAAATATCCCTATGGAAATT-ATAGAGTTGATGT-----TAACAG-GTC-  7027
                       *    *      **  *    ** **  ** **    *  *  *

1a-CP000114       6477 --GGTTCGAATGTGGGATAACACATTCGTCCACTGCGCTGGATTCTTTAACTTGTGTTGA  6534
1b-FO393392       6381 --GGTTCGAATTTGGGATAACACATTCGTCCACTGTGCTGGATTTTTTAACTTGTGTTGA  6438
2-AAJO01000077    7647 AAAATTTGATGTTGGGATTACATATTTTTCCACGGCATTAGATTTTTTAAATTGTGTTAA  7706
3-AL766849        6366 --GGTTCGAATGTGGGATAACACATTCGTCCACTGCGCTGGATTCTTTAACTTGTGTTGA  6423
4-AF355776        6965 --GGTTCGAATGTGGGATAACACATTCGTCCACCGCGCTGGATTTTTTAACTTGTGTTGA  7022
5-AE009948        7912 --GGTTCGAATTTGGGATAACACATTCGTCCACCGCGCTGGATTTTTTAACTTGTGTTGA  7969
6-HF952106        6242 --GGTTCGAATGTGGGATAACACATTCGTCTACTGCGCTGGACTTTTTAACTTGTGTTGA  6299
7-AY376403        6989 --GTTTCGAGGTTGGGATAACGCACTCATCCACTGCACTGGATTTTTTAACTTGTGTTGA  7046
8-AY375363        5742 ---AGCGGAATTTGAAGAAGAGCAATCGATT--TTTAATGAGTT--TAAACTTACTTTTG  5794
9-CGBY01000002    7028 --GGTTCGAATTTGGGATAACACATTCGTCCACCGCGCTGGATTTTTTAACTTGTGTTGA  7085
                              **   **         * *            *    *  * ** **   **

1a-CP000114       6535 TAAAAAAATAACTTTAGTAGATCTGAAGGATATTAAATGAATACTAAGAAATTACTTCAA  6594
1b-FO393392       6439 TAAAAAAATAACTTTAGTAGATCTGAAGGATATTAAATGAATACTAAGAAATTACTTCAA  6498
2-AAJO01000077    7707 TAAAAAAATTTTCTTATACAATATTAAGGATATTAAATGAATACTAAGAAATTACTTCAA  7766
3-AL766849        6424 TAAAAAAATAACTTTAGTAGATCTGAAGGATATTAAATGA                      6463
4-AF355776        7023 TAAAAAAATAACTTTAGTGGATCTGAAGGATATTAAATGAATACTAAAAAATTACTTCAA  7082
5-AE009948        7970 TAAAAAAATAACTTTAGTAGATCTGAAGGATATTAAATGAATACTAAGAAATTACTTCAA  8029
6-HF952106        6300 TAAAAAAATAACTTTAGTAGATCTGAAGGATATTAAATGAATACTAAGAAATTACTTCAA  6359
```

```
7-AY376403       7047 TAGAAAAAATAATTTTAGTGAATCTGAAGGATATTAAATGAATACTAAGAAATTACTTCAA  7106
8-AY375363       5795 T---ATGATATGTTCAAAAG----GAGGGA----GGATGAATACTAAGAAATTACTTCAA  5843
9-CGBY01000002   7086 TGAAAAAATAACTTTAGTAGATCTGAAGGATATTAAATGAATACTAAGAAATTACTTCAA  7145
                      *   *  **    * *         * ***      ****

1a-CP000114      6595 TCTGGTTTTATTTATACACTCGGGAATTTATTGGTGCAAGGATTAGCCTTTATCACACTT  6654
1b-FO393392      6499 TCTGGTTTTATTTATACAATCGGGAATTTGTTGGTGCAAGGATTAGCCTTTATCACACTT  6558
2-AAJO01000077   7767 TCTGGTTTTATTTATACACTCGGGAATTTATTGGTGCAAGGATTAGCCTTTATCACACTT  7826
3-AL766849       6464                                                               6463
4-AF355776       7083 TCTGGTTTTATTTATACACTCGGGAATTTATTGGTGCAAGGATTAGCCTTTATCACACTT  7142
5-AE009948       8030 TCTGGTTTTATTTATACACTCGGGAATTTATTGGTGCAAGGATTAGCCTTTATCACACTT  8089
6-HF952106       6360 TCTGGTTTTATTTATACAATCGGGAATTTGTTGGTGCAAGGATTAGCCTTTATCACACTT  6419
7-AY376403       7107 TCTGGTTTTATTTATACAATTGGAAATTTATTGGTGCAAGGGCTAGCCTTTATCACACTT  7166
8-AY375363       5844 TCTGGTTTTATTTATACAATCGGGAATTTGTTGGTGCAAGGATTAGCCTTTATCACACTT  5903
9-CGBY01000002   7146 TCTGGTTTTATTTATACACTCGGGAATTTATTGGTGCAAGGATTAGCCTTTATCACACTT  7205


1a-CP000114      6655 CCCATCTATACTCGGGTTATCTCAACAGAAGTTTATGGCCAATATAGTTTATATGTCGCT  6714
1b-FO393392      6559 CCTATCTATACTCGGGTTATCTCAACAGAAGTTTATGGCCAATATAGTTTATATGTTGCT  6618
2-AAJO01000077   7827 CCCATCTACACTCGGGTCATCTCAACAGAAGTTTATGGCCAATATAGTTTATATGTCGCT  7886
3-AL766849       6464                                                               6463
4-AF355776       7143 CCCATCTACACTCGAGTCATCTCAACAGAAGTTTATGGCCAATATAGTTTATATGTTGCT  7202
5-AE009948       8090 CCCATCTACACTCGAGTCATCTCAACAGAAGTTTATGGCCAATATAGTTTATATGTTGCT  8149
6-HF952106       6420 CCCATCTACACTCGGGTCATCTCAACAGAAGTTTATGGCCAATATAGTTTATATGTCGCT  6479
7-AY376403       7167 CCTATTTACACTCGGGTCATCTCAACAGAAGTTTATGGTCAATATAGTTTATATGTGGCT  7226
8-AY375363       5904 CCCATCTACACTCGGGTCATCTCAACAGAAGTTTATGGCCAATATAGTTTATATATCGCT  5963
9-CGBY01000002   7206 CCCATCTACACTCGAGTCATCTCAACAGAAGTTTATGGCCAATATAGTTTATATGTTGCT  7265


1a-CP000114      6715 TGGATGAATATTATTATGCTTTTTATTGGCCTTCAGACGAGCGGTTCTTTAAGTTCAGCC  6774
1b-FO393392      6619 TGGATGAATATTATTATGCTTTTTATTGGACTTCAGACGAGCGGTTCTTTAAGTTCAGCT  6678
2-AAJO01000077   7887 TGGATGAATATTATTATGCTTTTTATTGGCCTTCAGACGAGCGGTTCTTTAAGTTCAGCC  7946
3-AL766849       6464                                                               6463
4-AF355776       7203 TGGATGGGAATTATTACTCTGTTTATTGGTCTTCAGACGAGCGGTTCTTTAAGTTCGGCT  7262
5-AE009948       8150 TGGATGGGAATTATTACTCTGTTTATTGGTCTTCAGACGAGCGGTTCTTTAAGTTCGGCT  8209
6-HF952106       6480 TGGATGAATATTATTATGCTTTTTATTGGCCTTCAGACGAGCGGTTCTTTAAGTTCAGCC  6539
7-AY376403       7227 TGGATGGGGATCATTGCACTTTTTATTGGTCTTCAGACGAGCGGTTCTTTAAGTTCAGCC  7286
8-AY375363       5964 TGGATGAATATTATTATGCTTTTTATTGGCCTTCAGACGAGCGGTTCTTTAAGTTCAGCC  6023
9-CGBY01000002   7266 TGGATGGGAATTATTACTCTGTTTATTGGTCTTCAGACAAGCGGTTCTTTAAGTTCGGCT  7325


1a-CP000114      6775 CGGGTCAAATACGGAGAAGAATTTAAAAGCTATTCAGGGAGTGCCTTTTCTGTAGGTAAT  6834
1b-FO393392      6679 CGGGTCAAATATGGAGAAGAATTTAAAAGCTATTCTGGGAGTGCCTTTTCTGTAGGTAAT  6738
2-AAJO01000077   7947 CGGGTCAAATACGGAGAAGAATTTAAAAGCTATTCTGGGAGTGCCTTTTCTGTAGGTAAT  8006
```

```
3-AL766849       6464                                                               6463
4-AF355776       7263 CGAGTCAAATATGGAGAAGAGTTTAAAAGCTACTCTGGGAGTGCCTTTTCTGTAGGTAAT  7322
5-AE009948       8210 CGAGTCAAATATGGAGAAGAGTTTAAAAGCTACTCTGGGAGTGCCTTTTCTGTAGGTAAT  8269
6-HF952106       6540 CGGGTCAAATACGGAGAAGAATTTAAAAGCTATTCTGGGAGTGCCTTTTCTGTAGGTAAT  6599
7-AY376403       7287 CGGGTCAAATATGGAGAAGAGTTTAAAAGCTATTCTGGAAGTGCGTTTTCCGTAGGTAAT  7346
8-AY375363       6024 CGGGTCAAATACGGAGAAGAATTTAAAAGCTATTCTGGGAGTGCCTTTTCTGTAGGTAAT  6083
9-CGBY01000002   7326 CGAGTCAAATATGGAGAAGAGTTTAAAAGCTACTCTGGGAGTGCCTTTTCTGTAGGTAAT  7385


1a-CP000114      6835 ATATGGTTTCTTATTATACTTTTGATAGCTTTTCTATTTAGAAGTTTTCTTGCACCATTA  6894
1b-FO393392      6739 ATATGGTTTCTTATTGTACTTTTGATAGCTTTTCTATTTAGAAGTTTTCTTGCACCATTA  6798
2-AAJO01000077   8007 ATATGGTTTCTTATTATACTTTTGATAGCTTTTCTATTTAGAAGTTTTCTTGCACCATTA  8066
3-AL766849       6464                                                               6463
4-AF355776       7323 ATATGGTTTCTTATTGTACTTTTGATAGCTTTTCTATTTAGAAGTTTTCTTGCACCATTA  7382
5-AE009948       8270 ATATGGTTTCTTATTGTACTTTTGATAGCTTTTCTATTTAGAAGTTTTCTTGCACCATTA  8329
6-HF952106       6600 ATATGGTTTCTTATTGTACTTTTGATAGCTTTTCTATTTAGAAGTTTTCTTGCACCATTA  6659
7-AY376403       7347 ATATGGTTTCTTATTGTACTTTTGGTAGCTTTTCTATTTAGAAGTTTTTTGGCACCATTA  7406
8-AY375363       6084 ATATGGTTTCTTATTGTACTTTTGATAGCTTTTCTATTTAGAAGTTTTCTTGCACCATTA  6143
9-CGBY01000002   7386 GTATGGTTTCTTATTGTACTTTTGATAGCTTTTCTATTTAGAAGTTTTCTTGCACCATTA  7445


1a-CP000114      6895 GTTGGTTTTTCTGAATCTATTTTTTTATTAATGGTGTGTCAAAGTTACGCTAGCTATGTG  6954
1b-FO393392      6799 GTTGGTTTTTCTGAATCTATTTTTTTATTAATGGTGTGTCAAAGTTACGCTAGCTATGTG  6858
2-AAJO01000077   8067 GTTGGTTTTTCTGAATCTATTTTTTTATTAATGGTGTGTCAAAGTTACGCTAGCTATGTG  8126
3-AL766849       6464                                                               6463
4-AF355776       7383 GTTGGTTTTTCTGAATCTATTTTTTTATTAATGGTGTGTCAAAGTTACGCTAACTATGTG  7442
5-AE009948       8330 GTTGGTTTTTCTGAATCTATTTTTTTATTAATGGTGTGTCAAAGTTACGCTAACTATGTG  8389
6-HF952106       6660 GTTGGTTTTTCTGAATCTATTTTTTTATTAATGGTGTGTCAAAGTTACGCTAACTATGTG  6719
7-AY376403       7407 GTTGGTTTTTCTGAATCTATTTTTTTATTAATGATATGTCAGAGTTATGCTAACTATGTT  7466
8-AY375363       6144 GTTGGTTTTTCTGAATCTATTTTTTTATTAATGGTGTGTCAAAGTTACGCTAACTATGTG  6203
9-CGBY01000002   7446 GTTGGTTTTTCTGAATCTATTTTTTTATTAATGGTGTGTCAAAGTTACGCTAACTATGTG  7505


1a-CP000114      6955 GTGACTTTCTTTGGTCAGTATTTTATACAACAACAGAGGAGTTTGGCTAATTTAATATTA  7014
1b-FO393392      6859 GTGACTTTCTTTGGTCAGTATTTTATACAACAACAGAGGAGTTTGGCTAATTTAATATTA  6918
2-AAJO01000077   8127 GTGACTTTCTTTGGTCAGTATTTTATACAACAACAGAGGAGTTTGGCTAATTTAATATTA  8186
3-AL766849       6464                                                               6463
4-AF355776       7443 GTGACTTTCTTTGGTCAGTATTTTATACAACAACAGAGGAGCTTGGCTAATTTAATACTA  7502
5-AE009948       8390 GTGACTTTCTTTGGTCAGTATTTTATACAACAACAGAGGAGCTTGGCTAATTTAATACTA  8449
6-HF952106       6720 GTGACTTTCTTTGGTCAGTATTTTATACAACAACAGAGGAGCTTGGCTAATTTAATACTA  6779
7-AY376403       7467 GTAACTTTCTTTGGCCAATATTTTATACAGCAACAGAGGAGTTTTGCAAATTTAATACTA  7526
8-AY375363       6204 GTGACTTTCTTTGGTCAGTATTTTATACAACAACAGAGGAGCTTGGCTAATTTAATACTA  6263
9-CGBY01000002   7506 GTGACTTTCTTTGGTCAGTATTTTATACAACAACAGAGGAGCTTGGCTAATTTAATACTA  7565
```

```
1a-CP000114      7015 TCCTTAGCCAATGCAGTTTCATCTGTTGCACTATCTCTATTTTTAATTTTTCATTGGTCC  7074
1b-FO393392      6919 TCCTTAGCCAATGCAATTTCATCTGTTGCACTATCTCTATTTTTAATTTTTCATTGGTCC  6978
2-AAJO01000077   8187 TCCTTAGCCAATGCAGTTTCATCTGTTGCACTATCTCTATTTTTAATTTTTCATTGGTCC  8246
3-AL766849       6464                                                               6463
4-AF355776       7503 TCCTTAGCCAATGCAGTTTCATCTGTTGCACTATCTCTATTTTTAATTTTTCATTGGTCC  7562
5-AE009948       8450 TCCTTAGCCAATGCAGTTTCATCTGTTGCACTATCTCTATTTTTAATTTTTCATTGGTCC  8509
6-HF952106       6780 TCCTTAGCCAATGCAGTTTCATCTGTTGCACTATCTCTATTTTTAATTTTTCATTGGTCC  6839
7-AY376403       7527 TCCTTAACTAATGCTGTTTCATCTGTTGCATTATCACTATTTTTGATTTTTCATTGGTCT  7586
8-AY375363       6264 TCCTTAGCCAATGCAGTTTCATCTGTTGCACTATCTCTATTTTTAATTTTTCATTGGTCC  6323
9-CGBY01000002   7566 TCCTTAGCCAATGCAGTTTCATCTGTTGCACTATCTCTATTTTTAATTTTTCATTGGTCC  7625


1a-CP000114      7075 GATGACTTTTTATCTAGGGTTTTTGGAGCTTTTGTTCCTACTATAATAACTGGAATAGTT  7134
1b-FO393392      6979 GATGACTTTTTATCTAGGGTTTTTGGAGCTTTTGTTCCTACTATAATAACTGGAATAGTT  7038
2-AAJO01000077   8247 GATGACTTTTTATCTAGGGTTTTGGGAGCTTTTGTTCCTACTATAATAACTGGAATAGTT  8306
3-AL766849       6464                                                               6463
4-AF355776       7563 GATGACTTTTTATCTAGGGTTTTGGGAGCTTTTGTTCCTACTATAATAACTGGAATAGTT  7622
5-AE009948       8510 GATGACTTTTTATCTAGGGTTTTTGGAGCTTTTGTTCCTACTATAATAACTGGAATAGTT  8569
6-HF952106       6840 GATGACTTTTTATCTAGGGTTTTGGGAGCTTTTGTTCCTACTATAATAACTGGAATAGTT  6899
7-AY376403       7587 GATGATTTTCTGTCTAGGGTTTTTGGAGCTTTTGTTCCTACTATAATAGTTGCAATAGTT  7646
8-AY375363       6324 GATGACTTTTTATCTAGGGTTTTTGGAGCTTTTGTTCCTACTATAATAACTGGAATAGTT  6383
9-CGBY01000002   7626 GATGACTTTTTATCTAGGGTTTTTGGAGCTTTTGTTCCTACTATAATAACTGGAATAGTT  7685


1a-CP000114      7135 GCCTTTGCTTATATTTATTATCATAGCAAATCTTTTTACAATCCTAAGTATTTTCGGTTC  7194
1b-FO393392      7039 GCCTTTGCTTATATTTATTATCATAGCAAATCTTTTTACAATCCTAAGTATTTTCGGTTC  7098
2-AAJO01000077   8307 GCCTTTGCTTATATTTATTATCATAGCAAATCTTTTTACAATCCTAAGTATTTTCGGTTC  8366
3-AL766849       6464                                                               6463
4-AF355776       7623 GCCTTTGCTTATATTTATTATCATAGCAAATCTTTTTACAATCCTAAGTATTTTCGGTTC  7682
5-AE009948       8570 GCCTTTGCTTATATTTATTATCATAGCAAATCTTTTTACAATCCTAAGTATTTTCGGTTC  8629
6-HF952106       6900 GCCTTTGCTTATATTTATTATCATAGCAAATCTTTTTACAATCCTAAGTATTTTCGGTTC  6959
7-AY376403       7647 GCCTTCGGTTATATTTATTATCATAGTAAATCTTTTTACAATTCTAAGTATTTTCGGTTC  7706
8-AY375363       6384 GCCTTTGCTTATATTTATTATCATAGCAAATCTTTTTACAATCCTAAGTATTTTCGGTTC  6443
9-CGBY01000002   7686 GCCTTTGCTTATATTTATTATCATAGTAAATCTTTTTACAATCCTAAGTATTTTCGGTTC  7745


1a-CP000114      7195 ATTGTCACTGTGTCTGTTCCTTTGATTTTTCATTTGTTAGGACACCAGTTGTTGGGGCAA  7254
1b-FO393392      7099 ATTGTCACTGTGTCTGTTCCTTTGATTTTTCATTTGTTAGGACACCAGTTGTTGGGGCAA  7158
2-AAJO01000077   8367 ATTGTCACTGTGTCTATTCCTTTGATTTTTCATTTGTTAGGACATCAGTTATTAAATCAA  8426
3-AL766849       6464                                                               6463
4-AF355776       7683 ATTGTCACTGTGTCTATTCCTTTGATTTTTCATTTGTTAGGACATCAGTTATTAAATCAA  7742
5-AE009948       8630 ATTGTCACTGTGTCTATTCCTTTGATTTTTCATTTGTTAGGACATCAGTTATTAAATCAA  8689
6-HF952106       6960 ATTGTCACTGTGTCTATTCCTTTGATTTTTCATTTGTTAGGACATCAGTTATTAAATCAA  7019
```

```
7-AY376403       7707 ATTATAACAGTATCTGTTCCTTTGATTTTTCATTTGTTAGGACATCAATTATTAAACCAA  7766
8-AY375363       6444 ATTGTCACTGTGTCTGTTCCTTTGATTTTCCATTTGTTAGGACACCAGTTGTTGGGGCAA  6503
9-CGBY01000002   7746 ATTGTCACTGTGTCTATTCCTTTGATTTTTCATTTATTAGGACATCAGTTATTAAATCAA  7805


1a-CP000114      7255 TTAGATAGAATTATGCTAGCTCGTTTATACAATACTAAAGAAGTTGCAATGTATAGTTTC  7314
1b-FO393392      7159 TTAGATAGGATTATGCTAGCTCGTTTATATAGTACTAAAGAAGTTGCAATGTATAGTTTC  7218
2-AAJO01000077   8427 TTAGATAGGATTATGCTAGTTCGCTTATATAGTACTAAAGAAGTTGCAATGTATAGTTTC  8486
3-AL766849       6464                                                               6463
4-AF355776       7743 TTAGATAGGATTATGCTAGCTCGCTTATATAGTACTAAAGAAGTTGCAATGTATAGTTTC  7802
5-AE009948       8690 TTAGATAGGATTATGCTAGCTCGCTTATATAGTACTAAAGAAGTTGCAATGTATAGTTTC  8749
6-HF952106       7020 TTAGATAGGATTATGCTAGCTCGCTTATATAGTACTAAAGAAGTTGCAATATATAGTTTC  7079
7-AY376403       7767 CTAGATAGAATCATGCTAGCTCGTTTATATAATACTAAAGAAGTCGCAATGTATAGTTTT  7826
8-AY375363       6504 TTAGATAGAATTATGCTAGCTCGTTTATATAATACTAAAGAAGTTGCAATGTATAGTTTC  6563
9-CGBY01000002   7806 TTAGATAGGATTATGCTAGCTCGCTTATATAGTACTAAAGAAGTTGCAATGTATAGTTTC  7865


1a-CP000114      7315 GGTTATTCGCTTGGAATGATTATTCAGATTGTTTTGAATAGTATTAATATGGCTTGGATT  7374
1b-FO393392      7219 GGTTATTCGCTTGGAATGATTATTCAGATTGTTTTGAATAGTATTAATATGGCTTGGATT  7278
2-AAJO01000077   8487 GGTTATTCGCTTGGAATGATTATTCAGATTGTTTTGAATAGTATTAATATGGCTTGGATT  8546
3-AL766849       6464                                                               6463
4-AF355776       7803 GGTTATTCGCTTGGAATGATTATTCAGATTGTTTTGAATAGTATTAATATGGCTTGGATT  7862
5-AE009948       8750 GGTTATTCGCTTGGAATGATTATTCAGATTGTTTTGAATAGTATTAATATGGCTTGGATT  8809
6-HF952106       7080 GGTTATTCGCTTGGAATGATTATTCAGATTGTTTTGAATAGTATTAATATGGCTTGGATT  7139
7-AY376403       7827 GGCTATTCACTTGGGATGATTATTCAGATTGTTTTGAATAGCATTAATATGGCTTGGATT  7886
8-AY375363       6564 GGTTATTCGCTTGGAATGATTATTCAGATTGTTTTGAATAGTATTAATATGGCTTGGATT  6623
9-CGBY01000002   7866 GGTTATTCGCTTGGAATGATTATTCAGATTGTTTTGAATAGTATTAATATGGCTTGGATT  7925


1a-CP000114      7375 CCATGGTTTTTTGATGCTAGAAAAGAAAAATTACTGCAACTTTCAACTTATATTAGCCGT  7434
1b-FO393392      7279 CCATGGTTTTTTGATGCTAGAAGAGAAAAATTACTGCAACTTTCAACTTATATTAGCCGT  7338
2-AAJO01000077   8547 CCATGGTTTTTTGATGCTAGAAAAGAAAAATTACTGCAACTTTCAACTTATATTAGCCGT  8606
3-AL766849       6464                                                               6463
4-AF355776       7863 CCATGGTTTTTTGATGCTAGAAAAGAAAAATTACTGCAACTTTCAACTTATGTTAGCCGT  7922
5-AE009948       8810 CCATGGTTTTTTGATGCTAGAAAAGAAAAATTACTGCAACTTTCAACTTATATTAGCCGT  8869
6-HF952106       7140 CCATGGTTTTTTGATGCTAGAAAAGAAAAATTACTGCAACTTTCAACTTATATTAGCCGT  7199
7-AY376403       7887 CCATGGTTTTTTGATGCTAGAAAAGCAAAATTACTACAGCTTCCAACTTATATTAGCCGT  7946
8-AY375363       6624 CCATGGTTTTTTGATGCTAGAAAAGAAAAATTACTGCAACTTTCAACTTATATTAGCCGT  6683
9-CGBY01000002   7926 CCATGGTTTTTTGATGCTAGAAAAGAAAAATTACTGCAACTTTCAACTTATATTAGCCGT  7985


1a-CP000114      7435 TATCTCTATTTAGGAGTTTTCTTAACATTAGGTTATTTGACTGTTTTTCCTGAGTTAGCT  7494
1b-FO393392      7339 TATCTCTATTTAGGAGTTTTCTTAACATTAGGTTATTTGACTGTTTTTCCTGAGTTAGCT  7398
2-AAJO01000077   8607 TATCTCTATTTAGGAGTTTTCTTAACATTAGGTTATTTGACTGTTTTTCCTGAGTTAGCT  8666
```

```
3-AL766849       6464                                                               6463
4-AF355776       7923 TATCTCTATTTAGGAGTTTTCTTAACATTAGGTTATTTGACTGTTTTTCCTGAGTTAGCT  7982
5-AE009948       8870 TATCTCTATTTAGGAGTTTTCTTAACATTAGGTTATTTGACTGTTTTTCCTGAGTTAGCT  8929
6-HF952106       7200 TATCTCTATTTAGGAGTTTTCTTAACATTAGGTTATTTGACTGTTTTTCCTGAGTTAGCT  7259
7-AY376403       7947 TATCTGTACTTAGGAGTTTTCTTAACTTTAGGCTATTTGACTATTTTCCCTGAATTAGCT  8006
8-AY375363       6684 TATCTCTATTTAGGAGTTTTCTTAACATTAGGTTATTTGACTGTTTTTCCTGAGTTAGCT  6743
9-CGBY01000002   7986 TATCTCTATTTAGGAGTTTTCTTAACATTAGGTTATTTAACTGTTTTTCCTGAGTTAGCT  8045


1a-CP000114      7495 CAAATAATGGGAGGAGACAAGTATAGTAGTAGTGTTCAATTTATTTCTTTAATTATTGTC  7554
1b-FO393392      7399 CAAATAATGGGAGGAGACAAGTATAGTAGTAGTGTTCAATTTATTTCTTTAATTATTGTC  7458
2-AAJO01000077   8667 CAAATAATGGGAGGAGACAAGTATAGTAGTAGTGTTCAATTTATTTCTTTAATTATTGTC  8726
3-AL766849       6464                                                               6463
4-AF355776       7983 CAAATAATGGGAGGAGACAAGTATAGTAGTAGTGTTCAATTTATTTCTTTAATTATTGTC  8042
5-AE009948       8930 CAAATAATGGGAGGAGACAAGTATAGTAGTAGTGTTCAATTTATTTCTTTAATTATTGTC  8989
6-HF952106       7260 CAAATAATGGGAGGAGACAAGTATAGTAGTAGTGTTCAATTTATTTCTTTAATTATTGTC  7319
7-AY376403       8007 CAAATAATGGGAGGAGACAAGTATAGTAGTAGTGTTCAATTTATCTCTTTAATTATTGTC  8066
8-AY375363       6744 CAAATAATGGGAGGAGACAAGTATAGTAGTAGTGTTCAATTTATTTCTTTAATTATTGTC  6803
9-CGBY01000002   8046 CAAATAATGGGAGGAGACAAGTATAGTAGTAGTGTTCAATTTATTTCTTTAATTATTGTC  8105


1a-CP000114      7555 AGCTACTTTTTAGTTTTTCTCTATACATTTCCTGTAAATATCCAATTCTTCTATGCTAAT  7614
1b-FO393392      7459 AGCTATTTTTTAGTCTTTCTCTATACATTTCCTGTAAATATCCAATTCTTCTATGCTAAT  7518
2-AAJO01000077   8727 AGCTACTTTTTAGTCTTTCTCTATACATTTCCTGTAAATATCCAATTCTTCTATGCTAAT  8786
3-AL766849       6464                                                               6463
4-AF355776       8043 AGTTATTTTTTAGTCTTTCTCTATACATTTCCTGTAAATATCCAATTCTTCTATGCTAAT  8102
5-AE009948       8990 AGCTATTTTTTAGTCTTTCTCTATACATTTCCTGTAAATATCCAATTCTTCTATGCTAAT  9049
6-HF952106       7320 AGCTATTTTTTAGTCTTTCTCTATACATTTCCTGTAAATATCCAATTCTTCTATGCTAAT  7379
7-AY376403       8067 AGCTATTTTTTGGTCTTTCTATATACATTCCCTGTAAATATCCAATTTTTCTATGCTAAT  8126
8-AY375363       6804 AGCTACTTTTTAGTTTTTCTCTATACATTTCCTGTAAATATCCAATTCTTCTATGCTAAT  6863
9-CGBY01000002   8106 AGCTACTTTTTAGTCTTTCTCTATACATTTCCTGTAAATATCCAATTCTTCTATGCCAAT  8165


1a-CP000114      7615 ACGACATGGATTCCTATTGGGACATTATTAGCTGCTGGTGTAAATTGGTTACTAAACCTT  7674
1b-FO393392      7519 ACGACATGGATTCCTATTGGGACATTATTAGCTGCTGGTGTAAATTGGTTACTAAACTTT  7578
2-AAJO01000077   8787 ACGACATGGATTCCTATTGGGACATTATTAGCTGCTGGTGTAAATTGGTTACTAAACCTT  8846
3-AL766849       6464                                                               6463
4-AF355776       8103 ACGACATGGATTCCTATTGGGACATTATTAGCTGCTGGTGTAAATTGGTTACTAAACCTT  8162
5-AE009948       9050 ACGACATGGATTCCTATTGGGACATTATTAGCTGCTGGTGTAAATTGGTTACTAAACCTT  9109
6-HF952106       7380 ACGACATGGATTCCTATTGGGACATTATTAGCTGCTGGTGTAAATTGGTTACTAAACCTT  7439
7-AY376403       8127 ACGACATGGATTCCTATTGGAACATTATTAGCTGCTGGTGTAAATTGGTTACTAAATCTT  8186
8-AY375363       6864 ACGACATGGATTCCTATTGGGACATTATTAGCTGCTGGTGTAAATTGGTTACTAAACCTT  6923
9-CGBY01000002   8166 ACGACATGGATTCCTATTGGGACCTTATTAGCTGCTGGTGTAAATTGGTTACTAAACCTT  8225
```

```
1a-CP000114      7675 GTATTAATTCCTCATTATGCAGCCTATGGCGCTGCTATGGCTACAATAATATCTTATTTA  7734
1b-FO393392      7579 GTATTAATTCCTCATTATGCAGCCTATGGCGCTGCTATGGCTACAATAATATCTTATTTA  7638
2-AAJO01000077   8847 GTATTAATTCCTCATTATGCAGCCTATGGCGCTGCTATGGCTACAATAATATCTTATTTA  8906
3-AL766849       6464                                                               6463
4-AF355776       8163 GTATTAATTCCTCATTATGCAGCCTATGGCGCTGCTATGGCTACAATAATATCTTATTTA  8222
5-AE009948       9110 GTATTAATTCCTCATTATGCAGCCTATGGCGCTGCTATGGCTACAATAATATCTTATTTA  9169
6-HF952106       7440 GTATTAATTCCTCATTATGCAGCCTATGGCGCTGCTATGGCTACAATAATATCTTATTTA  7499
7-AY376403       8187 GTATTAATTCCTAACTATGCAGCCTATGGCGCTGCTATGGCTACAGTAATATCTTATTTA  8246
8-AY375363       6924 GTATTAATTCCTCATTATGCAGCCTATGGCGCTGCTATGGCTACAATAATATCTTATTTA  6983
9-CGBY01000002   8226 GTATTAATTCCTCGTTATGCATCCTATGGCGCTGCTATGGCTACTATAATATCTTATTTA  8285


1a-CP000114      7735 GCCTTGTTGATTTTTCATCATATTGTTTCGAAAGTTAAATACCATTATTCAGATGTCTCA  7794
1b-FO393392      7639 GCCTTGTTGATTTTTCATCATATTGTTTCGAAAGTTAAATACCATTATTCAGATGTTTCA  7698
2-AAJO01000077   8907 GCCTTGTTGATTTTTCATCATATTGTTTCGAAAGTTAAATACCATTATTCAGATGTCTCA  8966
3-AL766849       6464                                                               6463
4-AF355776       8223 GCCTTGTTGATTTTTCATCATATTGTTTCGAAAGTTAAATACCATTATTCAGATGTCTCA  8282
5-AE009948       9170 GCCTTGTTGATTTTTCATCATATTGTTTCGAAAGTTAAATACCATTATTCAGATGTCTCA  9229
6-HF952106       7500 GCCTTGTTGATTTTTCATCATATTGTTTCGAAAGTTAGATACCATTATTCAGATGTCTCA  7559
7-AY376403       8247 GCTTTGTTGATTTTTCATCATATTGTTTCGAAGGTTAAATACCATTATTTAGATGTCTCA  8306
8-AY375363       6984 GCCTTGTTGATTTTTCATCATATTGTTTCGAAAGTTAAATACCATTATTCAGATGTCTCA  7043
9-CGBY01000002   8286 GCCTTGTTGATTTTTCATCATATTGTTTCGAAAGTTAAATACCATTATTCAGATGTCTCA  8345


1a-CP000114      7795 GTTAGGCAGTACATCATTTTAAGTGGTATAGTATTTAGTTATGCTATGTTAATGAACATG  7854
1b-FO393392      7699 GTTAGGCAGTACATCATTTTAAGTGGTACAGTATTTAGTTATGCTATGTTAATGAACATG  7758
2-AAJO01000077   8967 GTTAGGCAGTACATCATTTTAAGTGGTATAGTATTTAGTTATGCTATGTTAATGAACATG  9026
3-AL766849       6464                                                               6463
4-AF355776       8283 GTTAGGCAGTACATCATTTTAAGTGGTATAGTATTTAGTTATGCTATGTTAATGAACATG  8342
5-AE009948       9230 GTTAGGCAGTACATTATTTTAAGTGGTATAGTATTTAGTTATGCTATGTTAATGAACATG  9289
6-HF952106       7560 GTTAGGCAGTACATCATTTTAAGTGGTATAGTATTTAGTTATGCTATGTTAATGAACATG  7619
7-AY376403       8307 GTCAGGCAGTACATCATTTTAAGTGGTATAGTATTTAGTTATGCTATGTTAATGAACATG  8366
8-AY375363       7044 GTTAGGCAGTACATCATTTTAAGTGGTATAGTATTTAGTTATGCTATGTTAATGAACATG  7103
9-CGBY01000002   8346 GTTAGGCAGTACATCATTTTAAGTGGTATAGTATTTAGTTATGCTATGTTAATGAACATG  8405


1a-CP000114      7855 TTCCTAGATAATATCGTTATTAGATGGAGTTTAGGAATAATTATTCTTATAGTATATAGC  7914
1b-FO393392      7759 TTCCTAGATAATATCGTTATTAGATGGAGTTTAGGAATAATTATTCTTATAGTATATAGC  7818
2-AAJO01000077   9027 TTCCTAGATAATATCGTTATTAGATGGAGTTTAGGAATAATTATTCTTATAGTATATAGC  9086
3-AL766849       6464                                                               6463
4-AF355776       8343 TTCCTAGATAATATCGTTATTAGATGGAGTTTAGGAATAATTATTCTTATAGTATATAGC  8402
5-AE009948       9290 TTCCTAGGTAATATCGTTATTAGATGGAGTTTAGGAATAATTATTCTTATAGTATATAGC  9349
6-HF952106       7620 TTCCTAGATAATATCGTTATTAGATGGAGTTTAGGAATAATTATTCTTATAGTATATAGC  7679
```

```
7-AY376403       8367 TTCCTAGATAATATCGTGGTGAGATGGGGTTTAGGGATAATGATTCTTATAGTATACAGC  8426
8-AY375363       7104 TTCCTAGATAATATCGTTATTAGATGGAGTTTAGGAATAATTATTCTTATAGTATATAGC  7163
9-CGBY01000002   8406 TTCCTAGATAATATCGTTATTAGATGGAGTTTAGGAATAATTATTCTTATAGTATATAGC  8465


1a-CP000114      7915 ATTGTTTTTCAAAAAGTCATTTTAGACTTATTGAGTAAAAAAAGAAGGAGAAGATAA  7971
1b-FO393392      7819 ATTGTTTTTCAAAAAGTCATTTTAGACTTATTGAGTAAAAAAAGAAGGAGAAGATAA  7875
2-AAJO01000077   9087 ATTGTTTT-CAAAAAG                                           9101
3-AL766849       6464                                                            6463
4-AF355776       8403 ATTGTTTTTCAAAAAGTCATTTTAGACTTATTGAGTAAAAAAAGAAGGAGAAGATAA  8459
5-AE009948       9350 ATTGTTTTTCAAAAAGTCATTTTAGACTTATTGGGTAAAAAAAGAAGGAGAAGATAA  9406
6-HF952106       7680 ATTGTTTTTCAAAAAGTCATTTTAGACTTATTGAGTAAAAAAAGAAGGAGAAGATAA  7736
7-AY376403       8427 ATTGTTTTTCAAAAAGTCATCTTAGACTTATTGAGTAAAAAAAGAAGGAGAAGATAA  8483
8-AY375363       7164 ATTGTTTTTCAAAAAGTCATTTTAGACTTATTGAGTAAAAAAAGAAGGAGAAGATAA  7220
9-CGBY01000002   8466 ATTGTTTTTCAAAAAGTCATTTTAGGCTTATTGGGTAAAAAAAGAAGGAGAAG      8518
```
